# Supplementary material for: A non-catalytic scaffolding activity of hexokinase 2 contributes to EMT and metastasis
Source: Nat Commun. 2022 Feb 16;13:899. doi: 10.1038/s41467-022-28440-3 (PMC8850586; doi:10.1038/s41467-022-28440-3)

Supplementary Information

A non-catalytic scaffolding activity of Hexokinase 2 contributes to EMT and metastasis

Catherine S. Blaha[1*], Gopalakrishnan Ramakrishnan[1*], Sang-Min Jeon[2*], Veronique Nogueira[1*], Hyunsoo Rho[1], Soeun Kang[1], Prashanth Bhaskar[1], Alexander R. Terry[1], Alexandre F. Aissa[1], Maxim V. Frolov[1], Krushna C. Patra[1,6], R. Brooks Robey[3,4], and Nissim Hay[1,5]

[1]Department of Biochemistry and Molecular Genetics, College of Medicine, University of Illinois at Chicago, Chicago, IL 60607, USA; [2]College of Pharmacy, Ajou University Yeongtong-gu, Suwon-si, Gyeonggi-do, 443-749, Korea, [3]Veterans Affairs Medical Center, White River Junction, VT 05009, USA; [4]Geisel School of Medicine at Dartmouth, Hanover, NH 03755, USA, and [5]Research & Development Section, Jesse Brown VA Medical Center, Chicago, IL 60612, USA;

[6] Present address: Department of Cancer Biology; University of Cincinnati, Cincinnati OH 45267-0521

* Equal contributions

Supplementary Figures 1-15
Supplementary Figure 16: Uncropped WBs.
Supplementary Table 1: Antibody list, Recombinant protein list, Chemicals and Enzymes, Commercial Assay Kits
Supplementary Table 2: PRIMERS

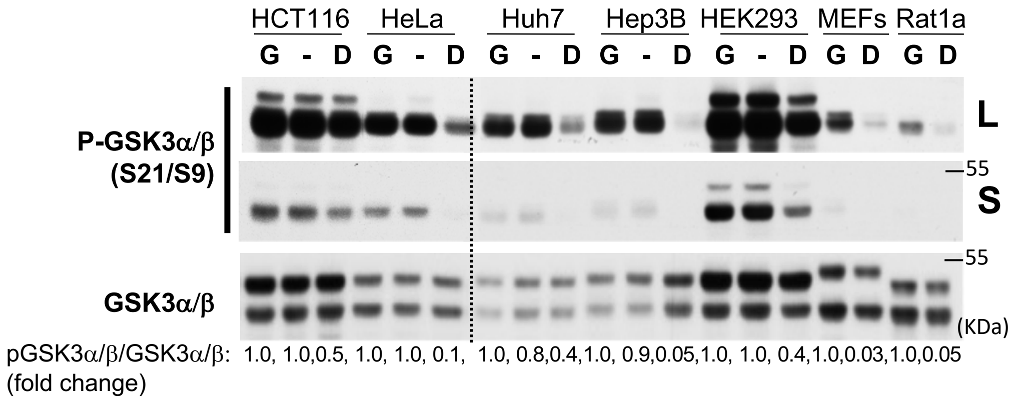

Supplementary Fig. 1: Cells were incubated in the presence of glucose (G), absence of glucose (-) or presence of 2-DG (D) for 2hr. An immunoblot image shows the phosphorylation of GSK3α and GSK3β. Similar results with individual cell lines were obtained in 2 independent experiments. Relative densitometric quantifications of pGSK3/GSK3 are shown.

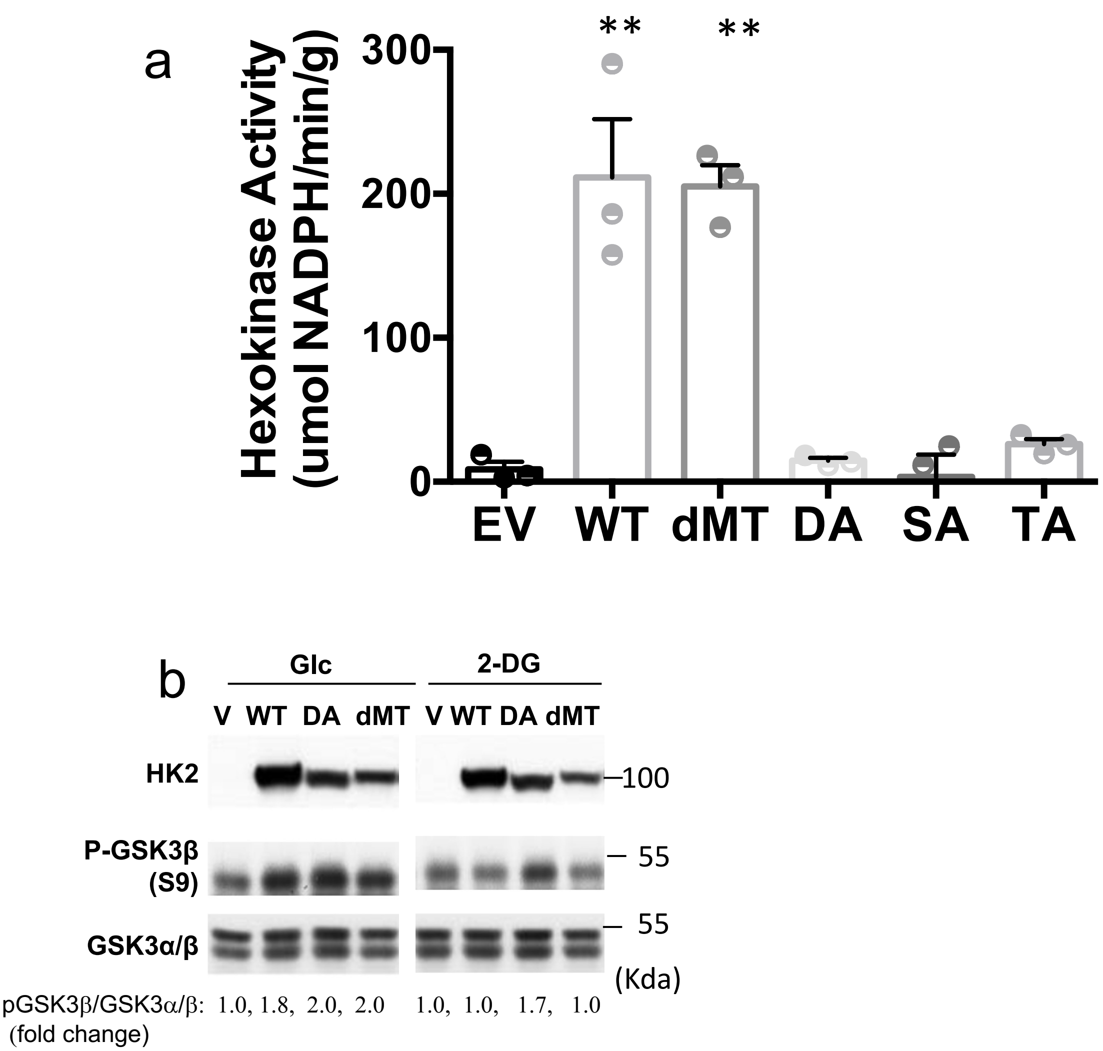

Supplementary Figure 2: a. Hexokinase activity in MI5-4 CHO cells expressing empty vector (EV), WT HK2 or HK2 mutants. Results are the mean ± SEM of 3 independent experiments in triplicate. **p<0.01 (p=0076 for WT, p=0.0002 for dMT, p=0.3461 for DA, p=0.7588 for SA, p=0.0521 for TA) all 2-sided t-test vs. EV.

b. MI5-4 CHO cells expressing either wild type (WT), kinase-dead HK2 mutant (DA), mitochondrial binding deficient mutant (dMT) or empty vector (V) were incubated in glucose free medium in the presence of 10mM glucose (G) or 2-DG (D). After 2hr, cells were harvested and analyzed by immunoblotting. Immunoblot represents 2 independent experiments

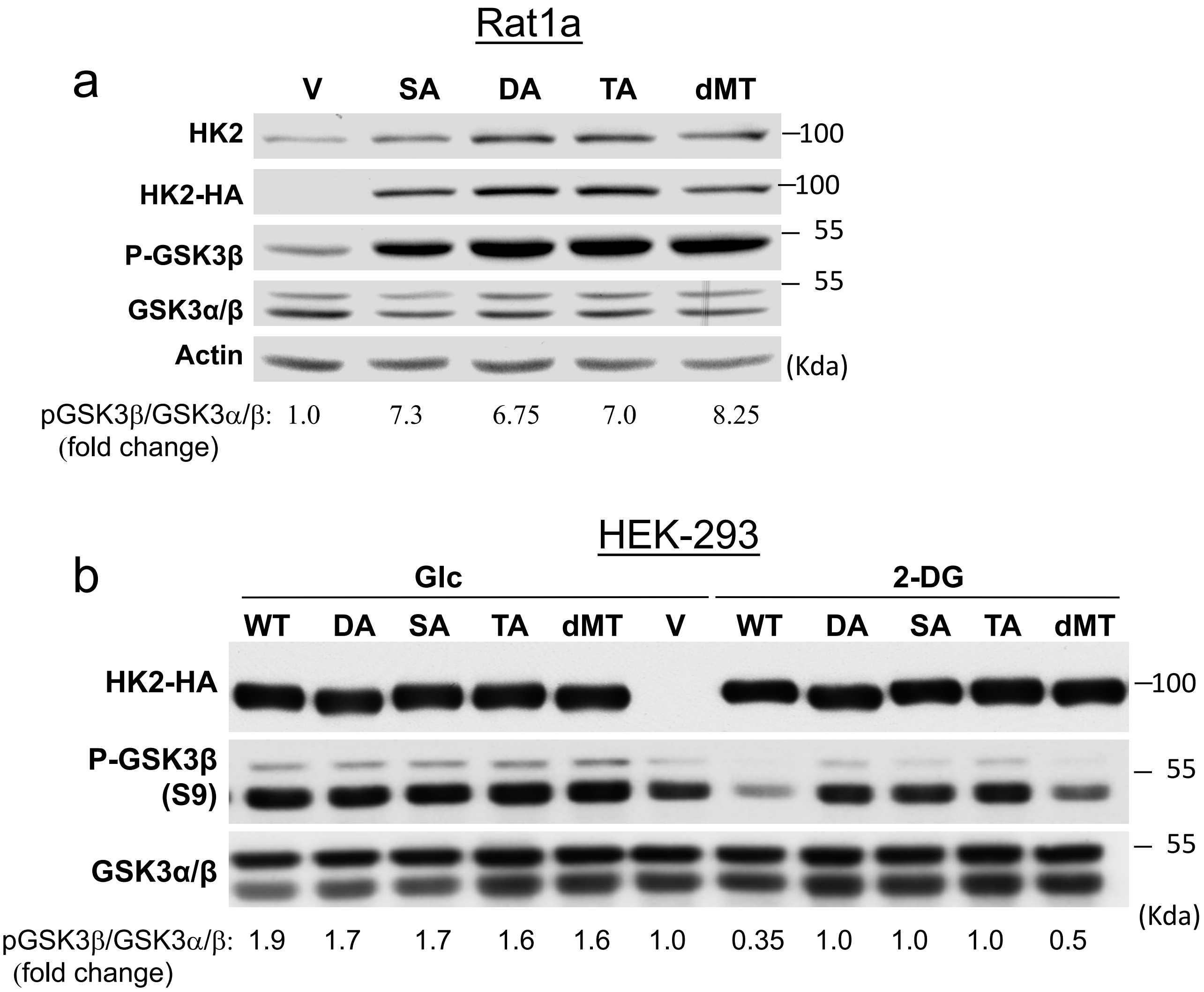

Supplementary Figure 3. Hexokinase maintains GSK3β phosphorylation independent of its activity, but its activity is required to suppress GSK3β phosphorylation by 2-DG.

a. The effect of WT HK2 and HK2 mutants overexpression on GSK3β phosphorylation in Rat1a cells (n=2).

b. The effect of 2-DG on GSK3β phosphorylation mediated by either WT HK2 or HK2 mutants in HEK293 cells (n=2).

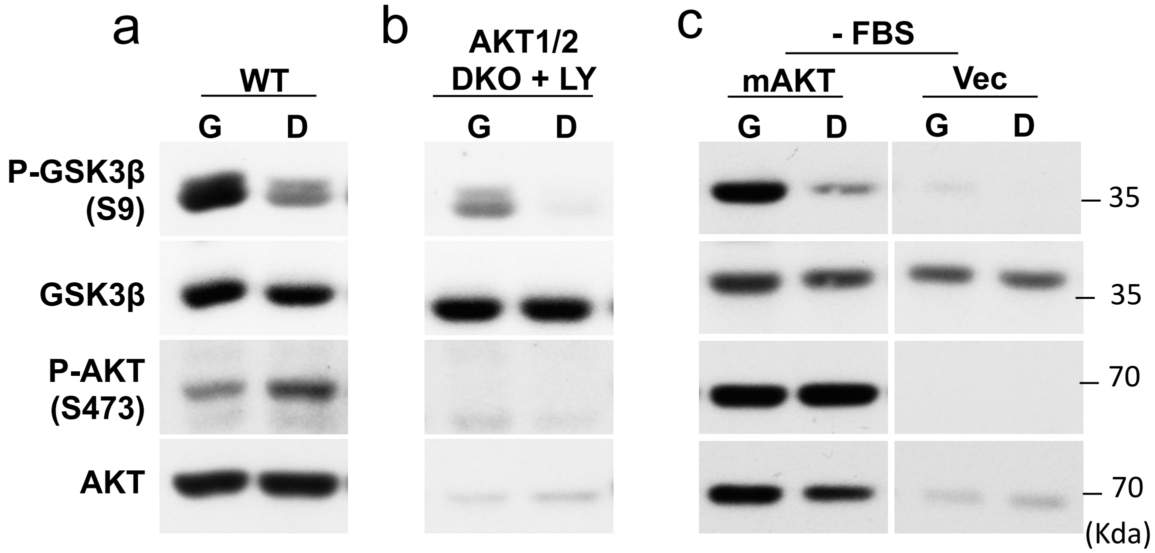

Supplementary Figure 4. The effect of HK2 on GSK3β phosphorylation is independent of Akt activity.

a. WT MEFs were incubated in glucose free medium in the presence of 10mM glucose (G) or 2-DG (D) followed by immunoblotting to determine GSK3β and Akt phosphorylation (n=3).

b. Akt1/2 DKO MEFs treated with LY294002 (LY) were incubated in glucose free medium in the presence of 10mM glucose (G) or 2-DG (D) followed by immunoblotting to determine GSK3β and Akt phosphorylation (similar results obtained in 3 different experiments).

c. MEFs expressing mAkt or vector control (Vec) were deprived of FBS and incubated in glucose free medium in the presence of 10mM glucose (G) or 2-DG (D) followed by immunoblotting to determine GSK3β and Akt phosphorylation (similar results obtained in 3 different experiments).

a

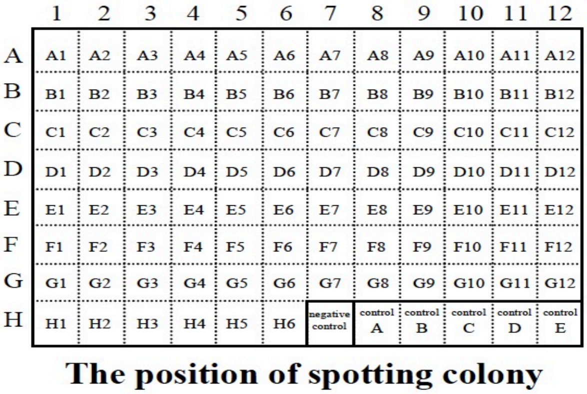

b

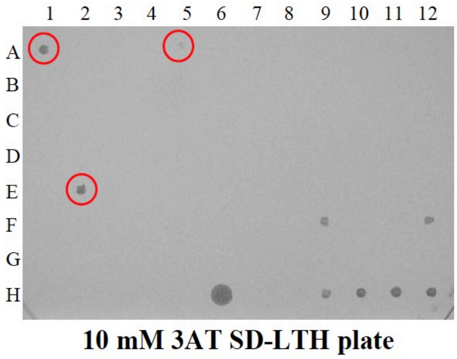

c

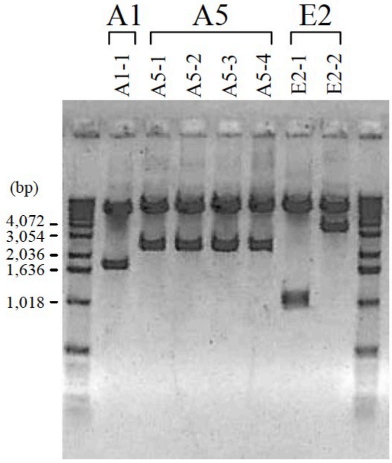

d

Clone	SD-LT	SD-LTH 10mM 3AT	β-gal assay	SD-LT-Ura	SD-LT+ 5FOA	Interaction strength in ProQuest System
A1-1	growth	-	white	-	growth	no interactor
A5-1	growth	-	white	-	growth	no interactor
E2-1	growth	-	white	-	growth	no interactor
E2-2	growth	growth	blue(weak)	growth(weak)	weak growth	Possible weak interactor

e

The insert sequence of E2-2

1 GGC GGG GCT GGG AGC AAA GCG CTG AGG GAG CTC GGT ACG CCG CCG
1 G G A G S K A L R E L G T P P

46 CCT CGC ACC CGC AGC CTC GCG CCC GCC GCC GCC CGT CCC CAG AGA
16 P R T R S L A P A A A R P Q R

91 ACC ATG GAG TCT GGC AGT ACC GCC GCC AGT GAG GAG GCA CGC AGC
31 T M E S G S T A A S E E A R S

137 CTT CGA GAA TGT GAG CTC TAC GTC CAG AAG CAT AAC ATT CAA GCG
46 L R E C E L Y V Q K H N I Q A

183 CTG CTC AAA GAT TCT ATT GTG CAG TTG TGC ACT GCT CGA CCT GAG
61 L L K D S I V Q L C T A R P E

229 AGA CCC ATG GCA TTC CTC AGG GAA TAC TTT GAG AGG TTG GAG AAG
76 R P M A F L R E Y F E R L E K

275 GAG GAG GCA AAA CAG ATT CAG AAT CTG CAG AAA GCA GGC ACT CGT
91 E E A K Q I Q N L Q K A G T R

321 ACA GAC TCA AGG GAG GAT GAG ATT TCT CCT CCT CCA CCC AAC CCA
106 T D S R E D E I S P P P P N P

367 GTG GTT AAA GGT AGG AGG CGA CGA GGT GCT ATC AGC GCT GAG GTC
121 V V K G R R R R G A I S A E V

413 TAC ACG GAG GAA GAT GCG GCA TCC TAT GTT AGA AAG GTT ATA CCA
136 Y T E E D A A S Y V R K V I P

459 AAA GAT TAC AAG ACA ATG GCC GCT TTA GCC 480
151 K D Y K T M A A L A

Supplementary Figure 5

Supplementary Figure 5: Yeast two-hybrid screen for HK2 interacting proteins (Performed by Invitrogen life technologies, Japan). Full length HK2 was cloned into the pDEST32 vector as a plasmid bait. Empty pDEST32 or pDEST32 expressing HK2 were transformed into MaV203 yeast competent cells. Large scale screening was performed under 10 mM 3AT concentration. After 4 days incubation, comparatively large 90 colonies were selected and cultured in 100 ul of SD-LTH medium with 10mM 3AT for one day using a 96-well plate, and then spotted on SD-LT plate, SD-LTH 10 mM 3AT plate, and nylon membrane on YPD plate for beta-gal assay. Prey plasmids, purified from possible positive clone, were introduced into E. coli and were estimated fragment size by colony PCR. Plasmids then were purified from the E. coli and transformed along with the bait or empty plasmid back into yeast and tested for all four phenotypes (sensitivity to 3AT, growth on medium without uracil, sensitivity to 5-FOA and detection of β-galactosidase activity). The insert of potential interactor was sequenced and then BLAST search was performed.

a. Position of spotting clones.

b. Possible positive clones (grown on 10mM 3AT plates); A1, A5, and E2 (F9, F12, and H6 were false positive.

c. Inserts in A1, A5 and E2 were amplified by PCR and cloned into plasmids. Insert size after cloning is shown.

d. Plasmids were re-validated for interaction with the HK2 bait, and one plasmid E2-2 was found as a real interactor.

e. The insert sequence of E2-2. Red labeled amino acids.

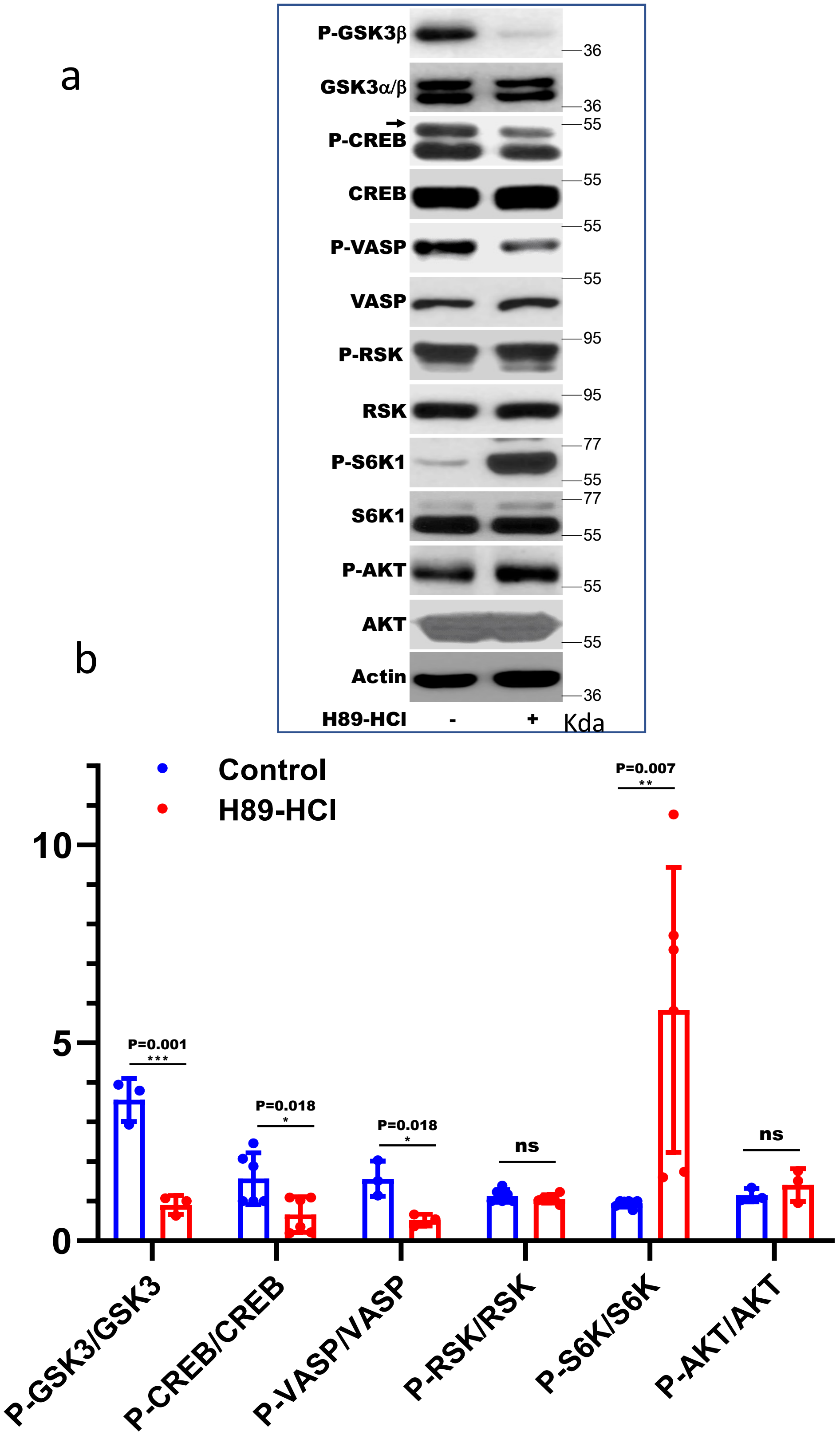

Supplementary Figure 6. The effect of H89 on PKA relative to other kinases. CHO-MI5-4 cells expressing HK2 were subjected to treatment with 10mM H89 for 2 hr, and proteins were extracted for immunoblotting. **a.** Representative immunoblot showing the phosphorylation of GSK3β, CREB and VASP as readouts for PKA activity, RSK, S6K1, and Akt. **b.** Quantification of phosphorylation: GSK3b (n=3), CREB (n=6), VASP (n=3), RSK (n=6), S6K1 (n=6), and Akt (n=3). Results are the mean ± SEM (paired t test).

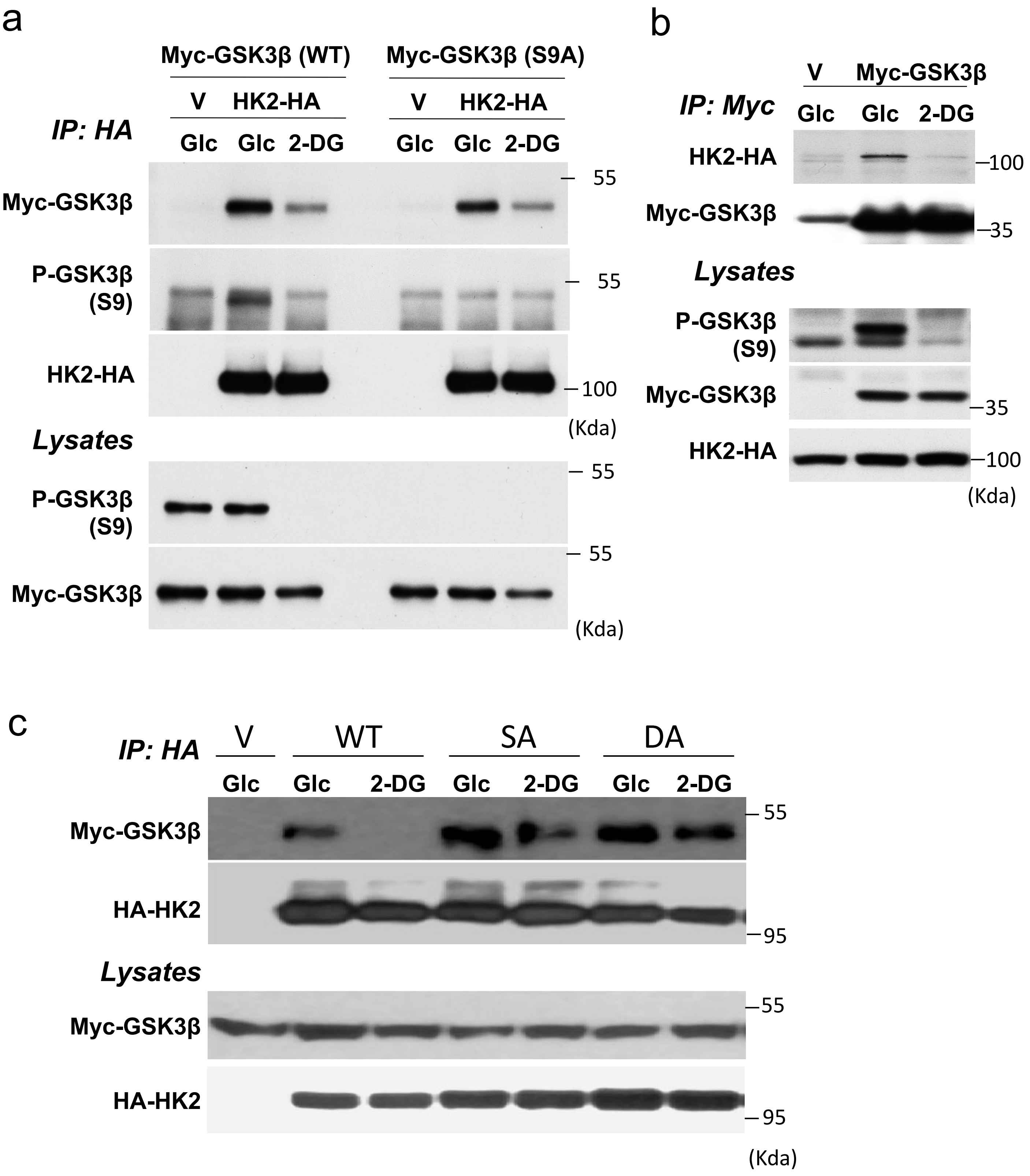
a
Myc-GSK3β (WT)
Myc-GSK3β (S9A)
V
HK2-HA
IP: HA
Glc
2-DG
Myc-GSK3β
P-GSK3β (S9)
HK2-HA
Lysates
55
100
(Kda)
b
IP: Myc
V
Myc-GSK3β
Glc
2-DG
HK2-HA
Myc-GSK3β
Lysates
P-GSK3β (S9)
100
35
(Kda)
c
IP: HA
V
WT
SA
DA
Glc
2-DG
Myc-GSK3β
HA-HK2
Lysates
55
95
(Kda)

Supplementary Figure 7. HK2 interacts with GSK3β in a 2DG-dependent manner.
a. After co-transfection of HK2-HA or HA-vector with Myc-GSK3β wild type (WT) or Myc-GSK3β nonphosphorylatable mutant (S9A) into HEK293 cells, the cells were incubated in glucose free medium in the presence of 10mM glucose (Glc) or 2-DG. After 2hr, cells were lysed for immunoprecipitation with anti-HA antibody followed by immunoblotting using anti-Myc-HRP, anti-HA and anti-P-GSK3β antibodies. Total lysates were subjected to immunoblotting using anti-P-GSK3β, and anti-Myc-HRP antibodies. Similar results obtained in 2 experiments.
b. After transfection of control Myc-vector or Myc-GSK3β plasmid into HEK293-HK2-HA expressing cells, the cells were incubated in glucose free medium in the presence of 10mM glucose (Glc) or 2-DG. After 2hr, cells were lysed for immunoprecipitation with anti-Myc antibody followed by immunoblotting using anti-HA and anti-Myc-HRP, antibodies. Total lysates were subjected to immunoblotting using anti-P-GSK3β, anti-Myc-HRP, and anti-HA antibodies. Similar results obtained in 2 experiments.
c. After transfection of Myc-GSK3β into MI5-4 CHO cells expressing either WT or mutants HK2, cells were incubated in glucose free medium in the presence of 10mM glucose (Glc) or 2-DG. After 2hr, cells were lysed for immunoprecipitation with anti-HA antibody followed by immunoblotting using anti-Myc-HRP and anti-HA and antibodies. Total lysates were subjected to immunoblotting using anti-Myc-HRP and anti-HA antibodies. Similar results obtained in 2 experiments.

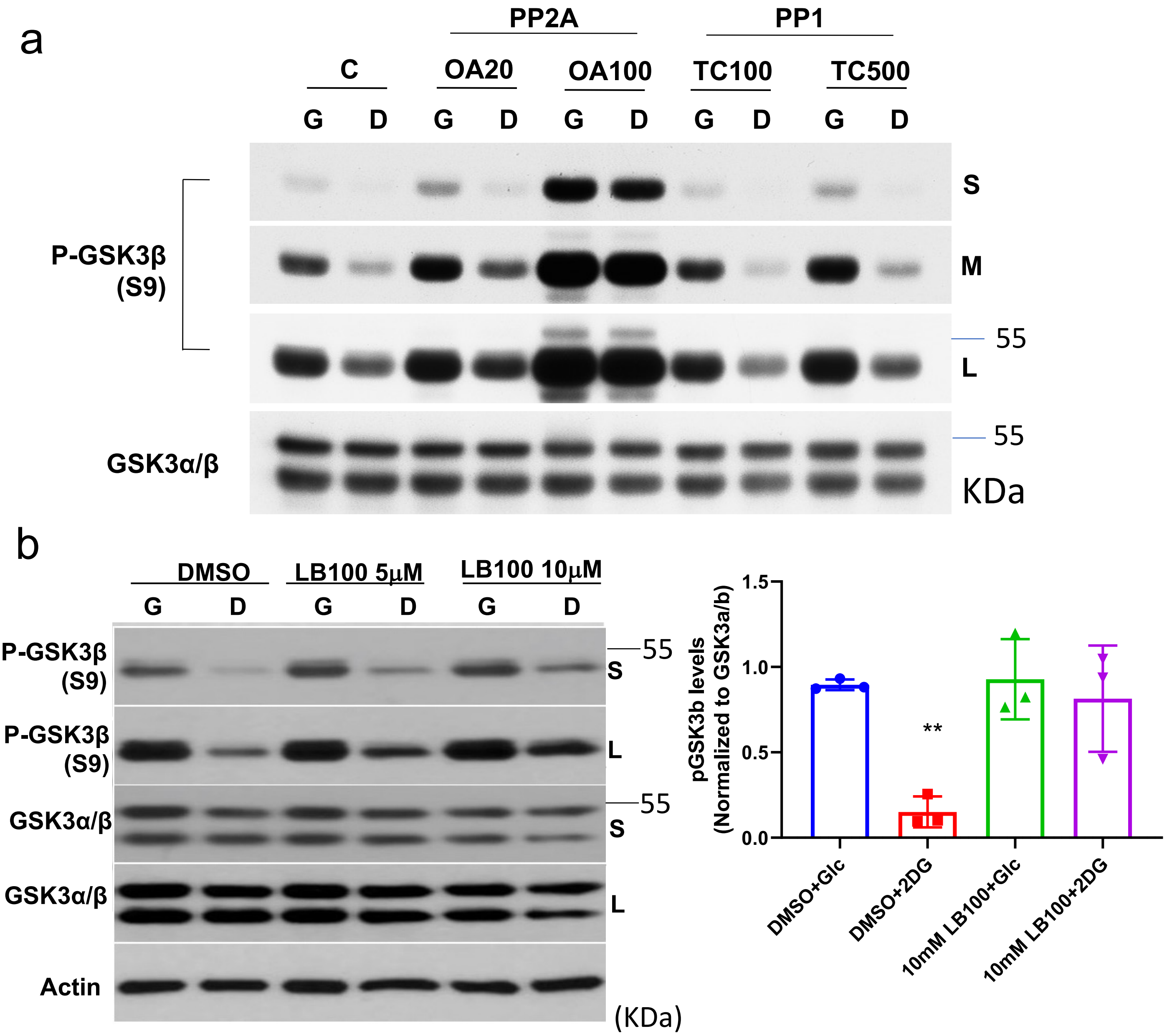

Supplementary Figure 8. The effect of protein phosphatases on GSK3β phosphorylation.

a. HeLa cells were incubated in glucose free medium in the presence of 10mM glucose (G) or 2-DG (D). DMSO (C), OA (20nM, 100nM), or TC (100nM, 500nM) were also treated with glucose or 2DG. After 2hr, cells were harvested and analyzed for immunoblotting using anti-P-GSK3β and anti-GSK3α/β (S-short exposure, M-medium exposure, L-long exposure). Similar results were obtained in 2 independent experiments.

b. Experiment was done as in a except that cells were treated with LB100. Bar graph shows densitometric quantification of pGSK3b/GSK3a/b ratio (S-short exposure, L-long exposure). Results are the mean ± SEM of 3 independent experiments; **p =0.007. One-way ANOVA test was used to calculate significant results.

a

b

c

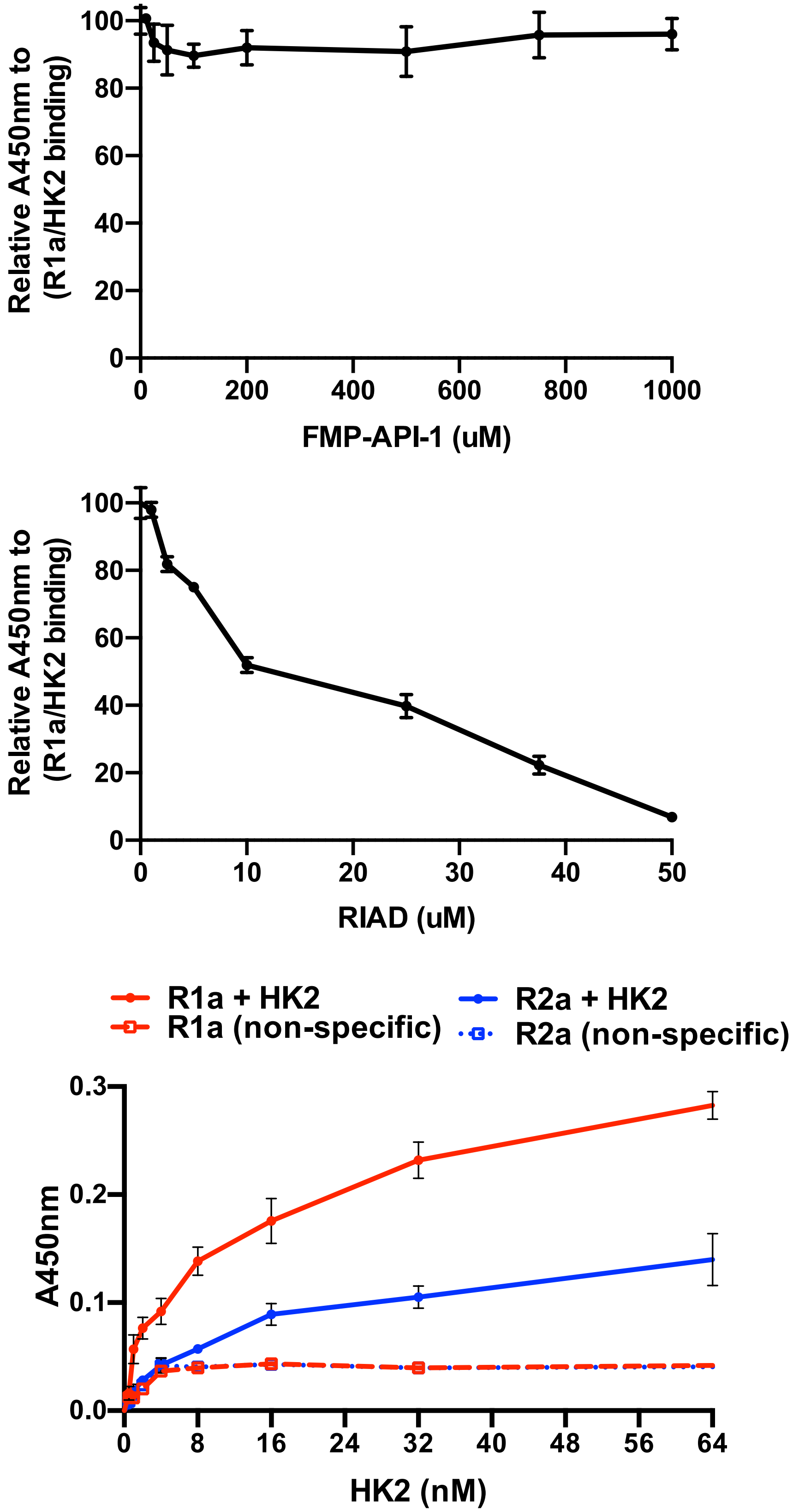

Supplementary Figure 9

Supplementary Figure 9. HK2 binds specifically R1a.

a. Nickel-coated 96-well plates were incubated overnight at 4°C with His-PRKAR1α (50 nM) and then incubated with Myc-HK2 (32 nM) for 2h. Thirty minutes before the end of the later incubation, increasing concentrations of FMP-API-1 (0 – 1000uM) are added to the wells. HK2 binding was detected with anti-Myc-HRP conjugated antibody, and an HRP-catalyzed reaction with a chromogenic substrate solution. Results are the mean ± SEM of 4 independent experiments (one way ANOVA $p<0.0001$).

b. Nickel-coated 96-well plates were incubated overnight at 4°C with His-PRKAR1α (50 nM) and then incubated with Myc-HK2 (32 nM) for 3h. Two hours before the end of the later incubation, increasing concentrations of RIAD (0 – 50uM) are added to the wells. HK2 binding was detected with anti-Myc-HRP conjugated antibody, and an HRP-catalyzed reaction with a chromogenic substrate solution. Results are the mean ± SEM of 4 independent experiments (one way ANOVA $p<0.0001$).

c. Nickel-coated 96-well plates were incubated overnight at 4°C with either His-PRKAR1a (50 nM) or His-PRKAR2a (50nM) and then incubated with Myc-HK2 (0.25– 64 nM) in blocking buffer or with blocking buffer without HK2. HK2 binding was detected with anti-Myc-HRP conjugated antibody, and an HRP-catalyzed reaction with a chromogenic substrate solution. Results are the mean ± SEM of 4 independent experiments. Curves were fitted based on a one-site-binding model in GraphPad Prism followed by a comparison of fits with $p<0.0001$

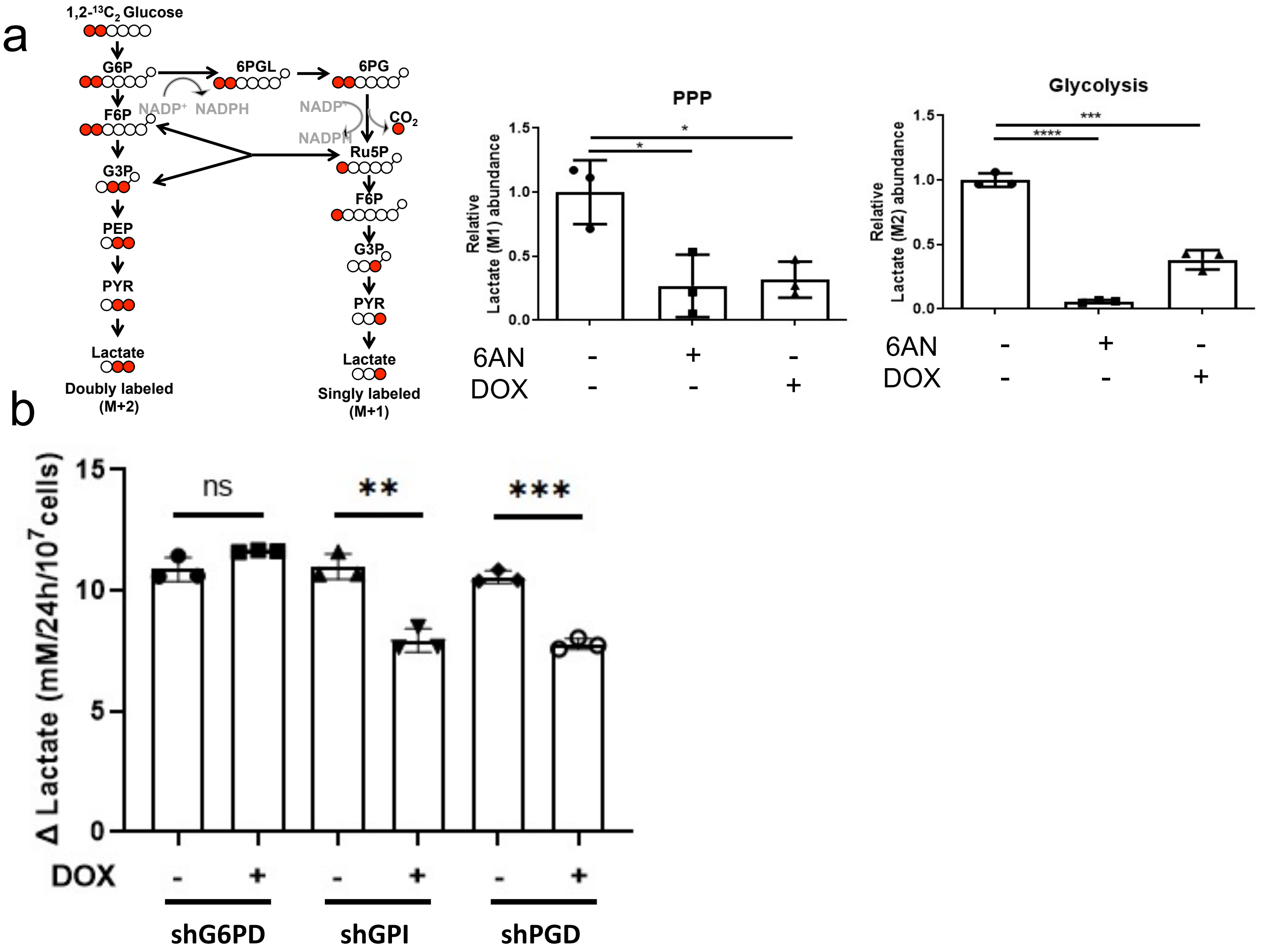

Supplementary Figure 10. a. Left panel: Simplified schematic of steps in glycolysis and the pentose phosphate pathway (PPP), showing ^{13}C labelling patterns resulting from [1,2-$^{13}C_2$]-glucose substrate and the conversion to M+1 and M+2 lactate. Red filled circles indicate ^{13}C atoms. Abbreviations: G6P, glucose-6-phosphate; 6PGL, 6-phosphogluconolactone; 6PG, 6-phosphogluconate; Ru5P, ribulose-5-phosphate; F6P, fructose-6-phosphate; G3P, glyceraldehyde-3-phosphate; PEP, phosphoenolpyruvate; PYR, pyruvate. Right panels: [1, 2-$^{13}C_2$]-glucose metabolic labelling in A549 cells showing the effect of 6-AN or the DOX-inducible knockdown of 6PDGH on the PPP and glycolysis (cells were treated with either 6-AN for 16h or with DOX for 4 days). Results show relative abundance of intracellular 1x^{13}C lactate (M+1) and 2x^{13}C lactate (M+2) after 25 mM [1, 2-$^{13}C_2$]-glucose labeling for 4 h. Results are the mean ± SEM of 3 independent experiments. *p<0.05, ***p<0.001, ****p<0.0001 versus control; unpaired t-test. P values were calculated using two-tailed unpaired t test;*P<0.05.***P<0.001,***P<0.001; exact P values are *P=0.0221 and *P=0.0144 for PPP,****P<0.0001 and ***P=0.003 for Glycolysis. **b.** Extracellular lactate production rates in A549 cells. The amount of extracellular lactate production in 24 h was normalized to viable cell number; cells were treated with or without 0.2ug/ml DOX for 6 days. Results are the mean ± SEM of 3 independent experiments. P values were calculated using two-tailed unpaired t-test. **P < 0.01, ***P < 0.001; exact P values are **P=0.0017, ***P=0.002. **P<0.005, ***P<0.0005.

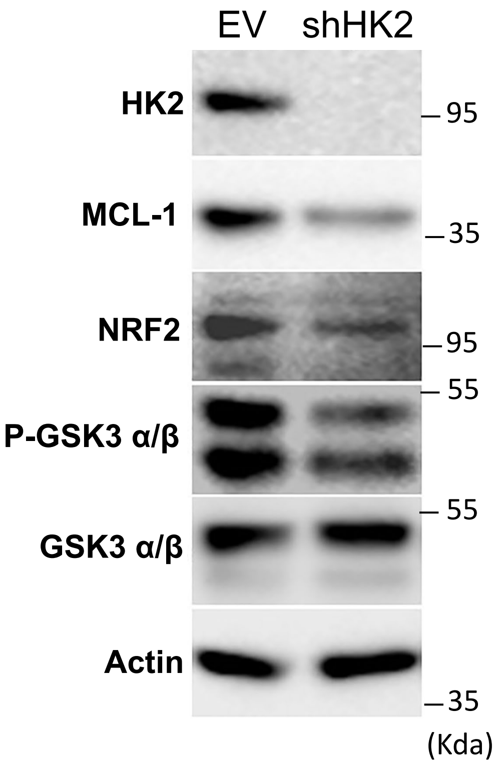

Supplementary Figure 11. Representative immunoblot image (n=2) showing p-GSK3β, MCL-1 and NRF2 levels after the knockdown of HK2 in A549 cells.

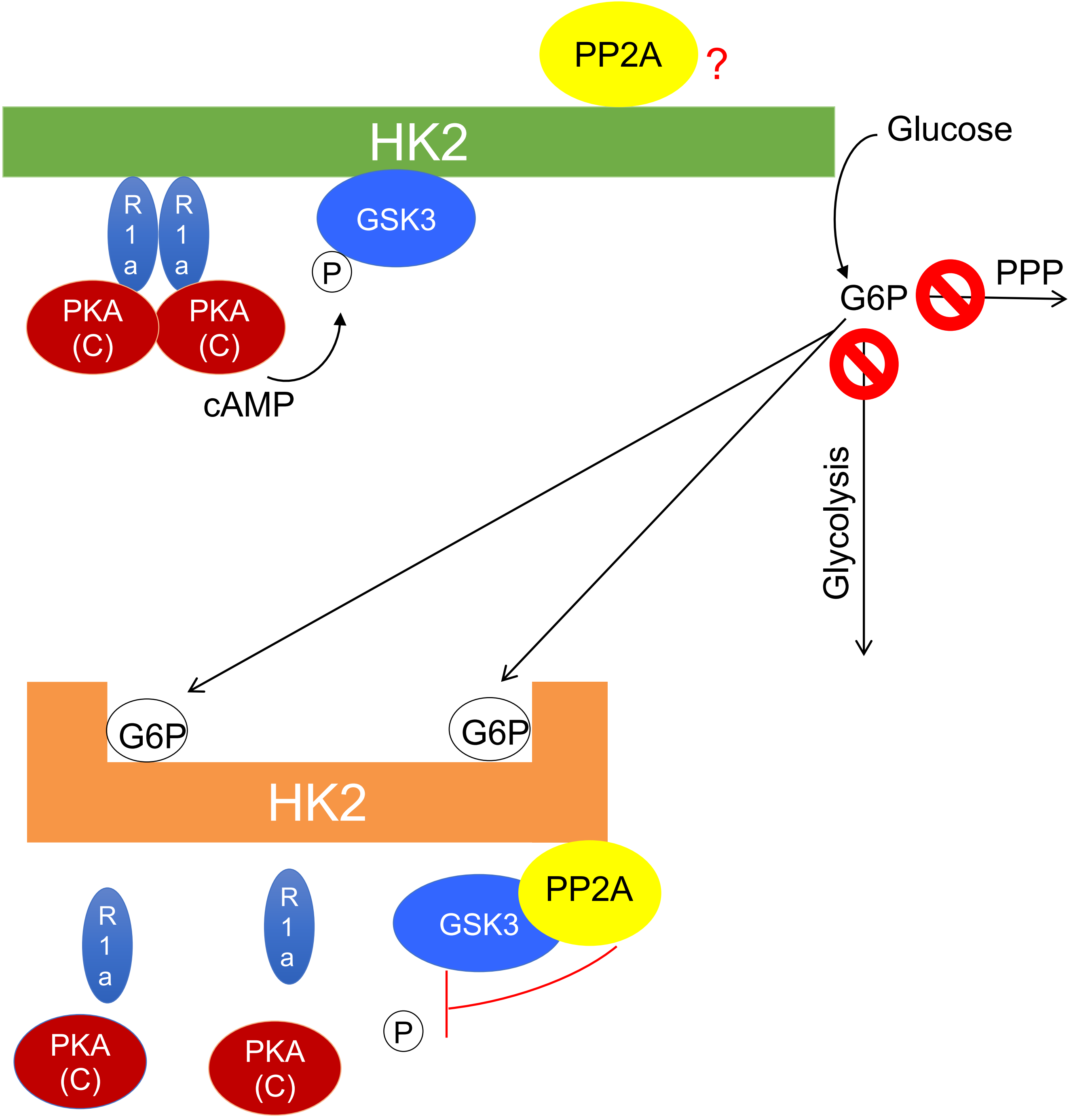

Supplementary Figure 12. A model depicting HK2 as a scaffold for GSK3 and PRKAR1a-PKA. When cells have high glucose flux, HK2 brings PKA and GSK3 into proximity. In the presence of cAMP, PKA is released from PRKAR1a to phosphorylate GSK3. When glucose flux is attenuated and G6P accumulates, an allosteric change is conferred to HK2 releasing GSK3 and PRKAR1a and increasing the availability of phosphorylated GSK3 to PP2A. It is possible that the amino-terminus half and the carboxy-terminus half of HK2 each binds GSK3 and PRKAR1a.

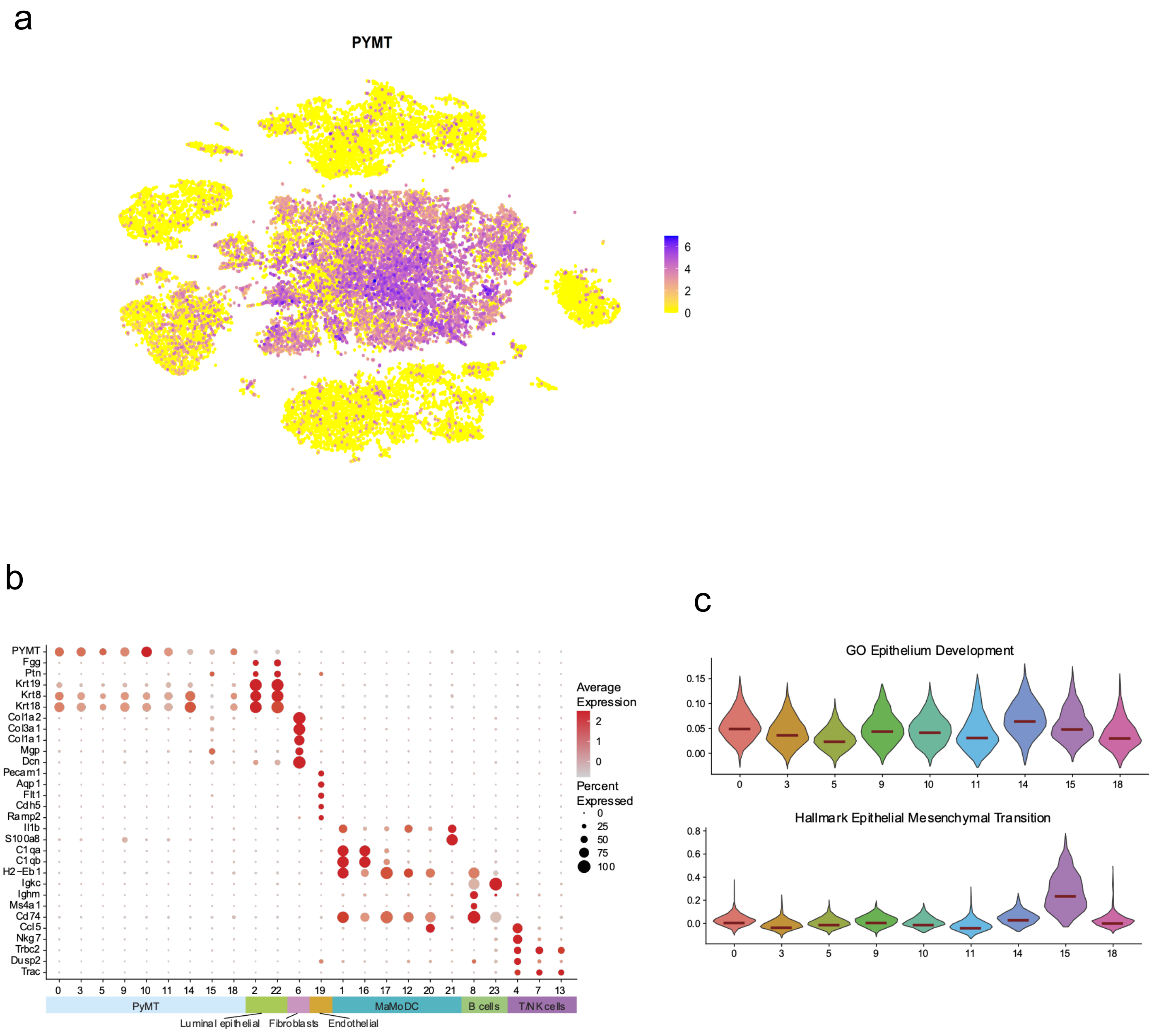

Supplementary Figure 13: Annotated clusters of pooled wild-type and HK2 deletion primary tumor samples.

a. Feature plot showing cells colored by the expression of PyMT.

b. Dot plot of expression for top cluster markers and cell type annotation for each cluster. The color of each dot represents the average expression level from low (gray) to high (red), and the size of each dot represents the percentage of the cells expressing the gene. MaMoDC: macrophages, monocytes and dendritic cells; B: B cells; T/NK: T and Natural Killer cells.

c. Violin plots of expression level for groups of genes related to "GO_EPITHELIUM_DEVELOPMENT" and "HALLMARK_EPITHELIAL_MESENCHYMAL_TRANSITION" (MSigDB Collections). The median of the data is shown by the horizontal line.

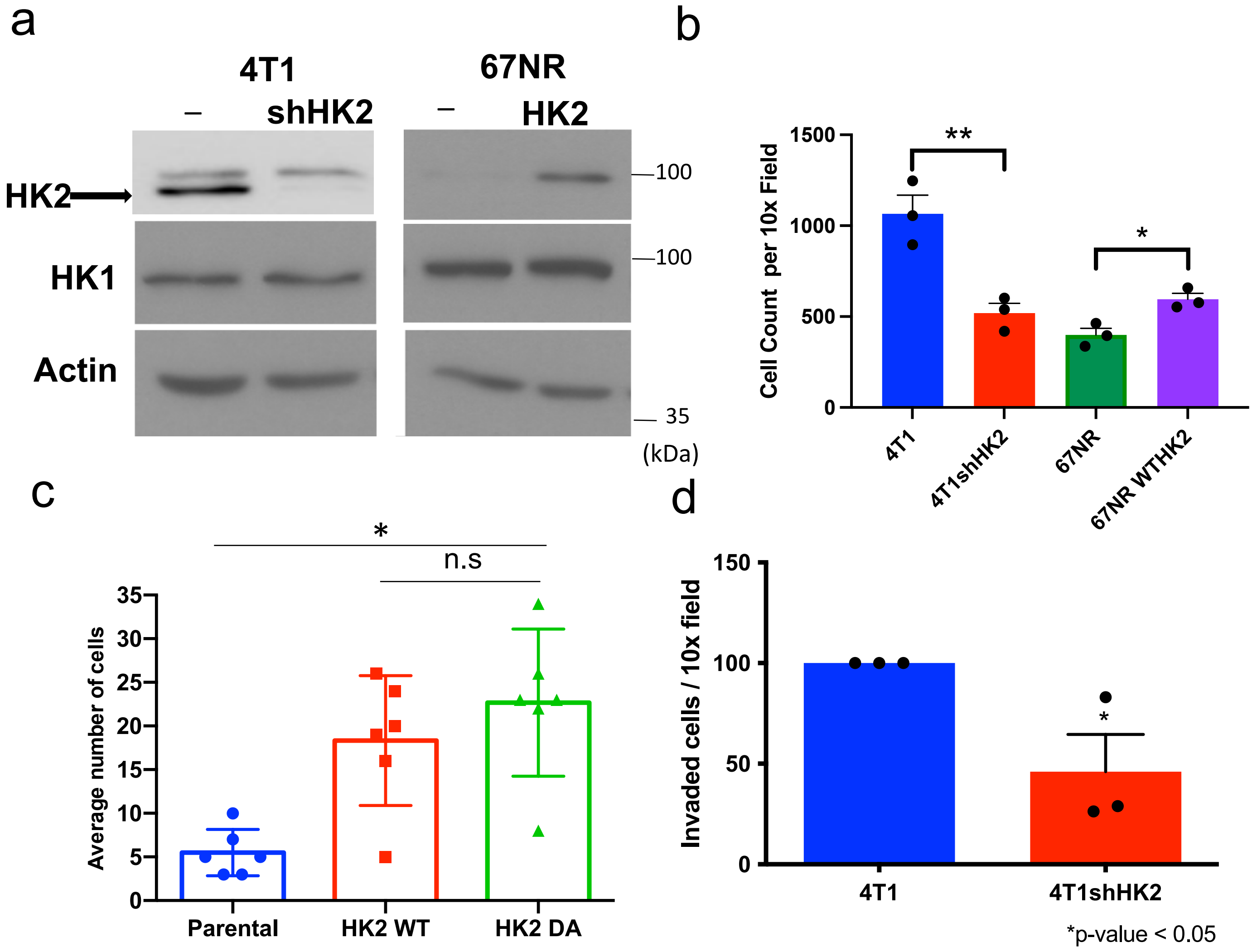

Supplementary Figure 14. HK2 silencing in 4T1 cells decreases transwell migration and invasion, while overexpression of HK2 in 67NR cells increases transwell migration.

a. Immunoblot showing (representative of 2 independent experiments) HK2 levels in 4T1 cells before and after silencing of HK2 and HK2 levels 67NR cells before and after overexpression of HK2.

b. Transwell migration comparing 4T1 shHK2 and 67NR WTHK2 cells to their parental cell lines. For transwell migration analysis, the cells were incubated in the upper transwell chambers for 12 hr with no serum while 20% serum was added to the lower chambers as a stimulant. Migrated cells in five random fields were counted after crystal violet staining, and three independent experiments with each group plated in triplicates were statistically analyzed. Quantified data represented as the mean ± SEM, *p<0.05, **p<0.01 (p=0.009 for 4T1 vs 4T1shHK2 and *p=0.015 for 67NR and 67NR WTHK2) using an unpaired two sided t-test.

c. Transwell migration analysis comparing 67NR cells to cells expression WT HK2 or HK2DA mutant. Quantified data represented as the mean ± SEM, *p<0.01 (*p =0.0013) from three independent experiments with each group plated in duplicates.

d. Transwell invasion of 4T1shHK2 compared to their parental cell line. Briefly, the cells were incubated in a gel-coated transwell chambers for 24 hr with no serum, and 20% serum was added to the lower chambers as a stimulant. The same numbers of cells were plated on control transwell chambers for migration. The percent areas of invaded and migrated cells stained with crystal violet in ten random fields were counted, and the percentage of invaded cells was calculated. Three independent experiments were performed. The data represent the mean ± SEM, *p =0.043 using unpaired two sided t-test.

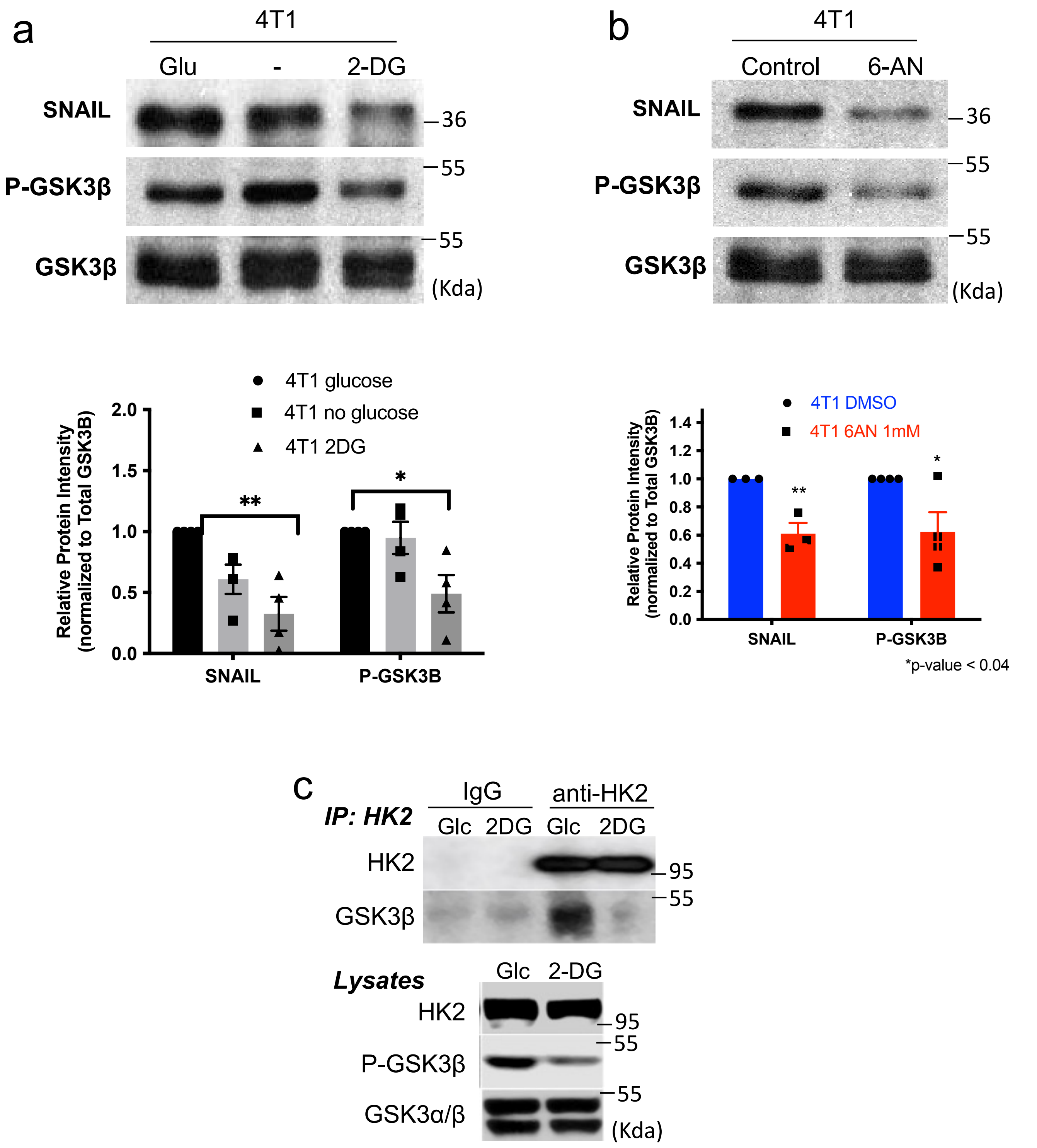

Supplementary Figure 15

Supplementary Figure 15. The effect 2-DG and 6AN treatment on SNAIL protein levels, GSK3 phosphorylation, and HK2-GSK3 binding.

a. 4T1 cells were treated with 10 mM 2-DG or glucose as a control for 2 hr and then subjected to immunoblotting. Data were normalized to the amount of total GSK3β, and fold changes shown using an unpaired two tailed t-test. are relative to the control glucose-treated 4T1 cells. Quantified western blot data from 2-DG treatment are represented as the means ± SEMs. *$p<0.05$, **$p<0.01$ ($p=0.0027$ for SNAIL and for P-GSK3β $p=0.016$) compared 2DG to glucose as the control from biologically 4 independent experiments

b. 4T1 cells were treated with 1 mM 6-AN for 12 hr and then subjected to immunoblotting to determine SNAIL and P-GSK3β levels. Quantified western blot data after 6-AN treatment are represented as the means ± SEMs. *$p<0.05$, **$p<0.01$ ($p=0.0068$ for SNAIL and $p=0.036$) for P-GSK3β from 3-4 biologically independent experiments using an unpaired two tailed t-test. Data were normalized to the amount of total GSK3β, and the fold changes shown are relative to the control DMSO-treated 4T1 cells. 4 replicates for P-GSK3β.

c. 4T1 cells were incubated in glucose free medium in the presence of 10mM glucose (Glc) or 2-DG. After 2hr, cells were lysed for immunoprecipitation. Endogenous HK2 was immunoprecipitated with anti-HK2 and subjected to immunoblotting with anti-HK2 and anti-GSK3β antibodies. Similar results were obtained in 2 independent experiments.

Supplementary Figure 16: Uncropped WBs

Fig. 1a

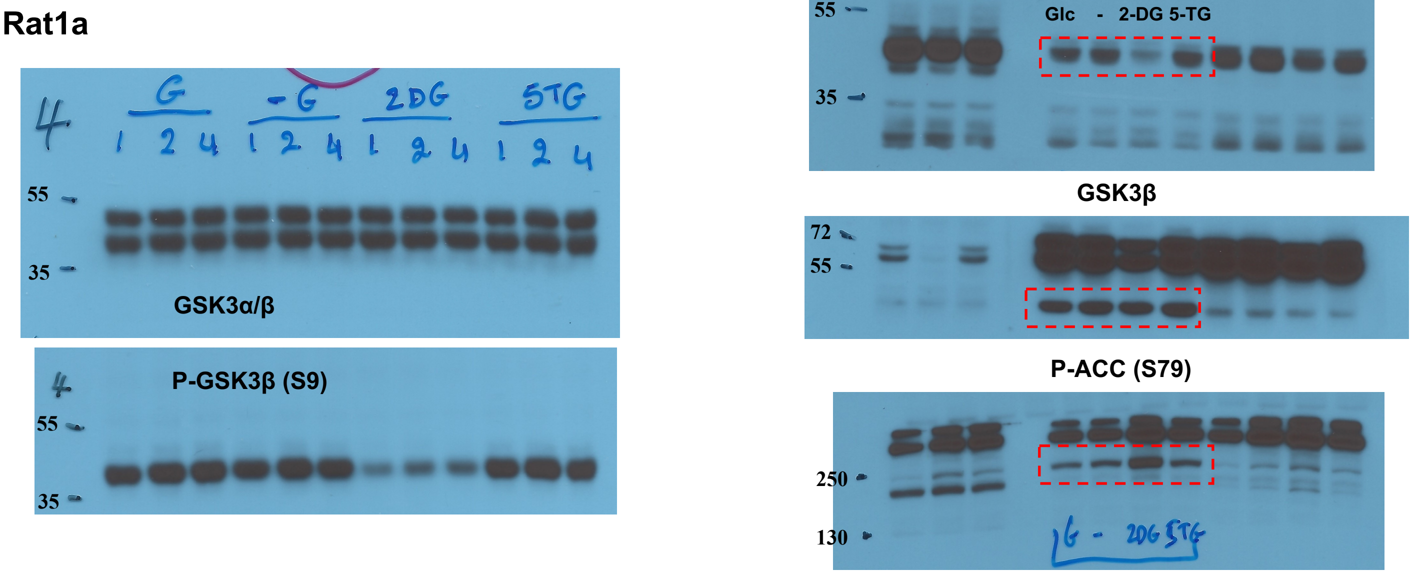

Additional experiments for quantifications

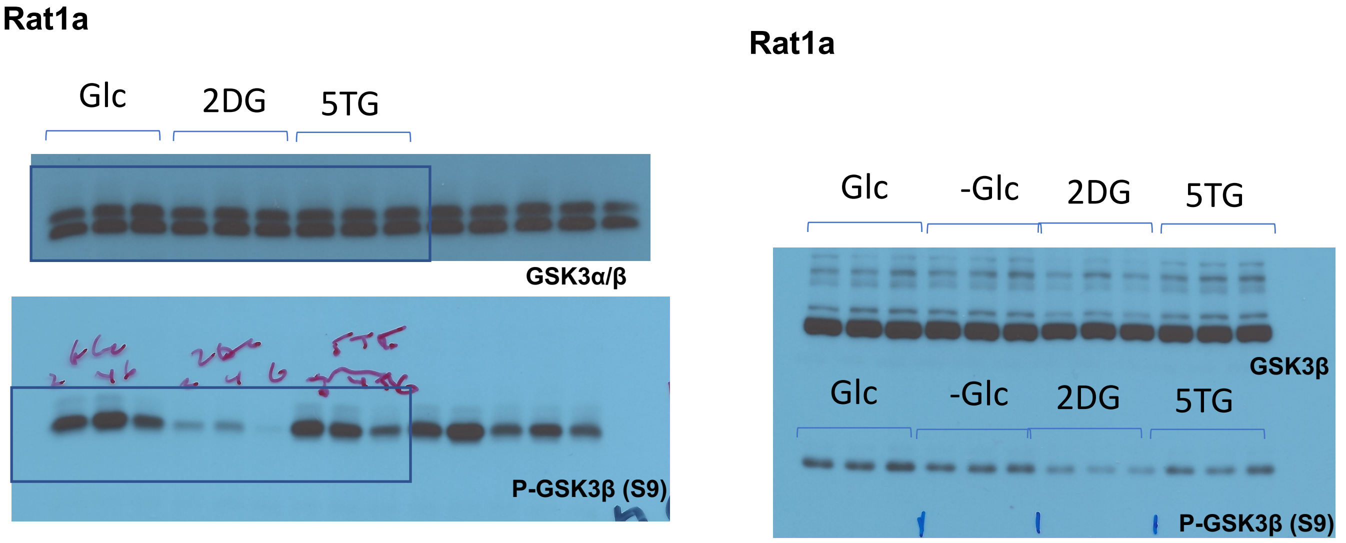

Fig. 1c

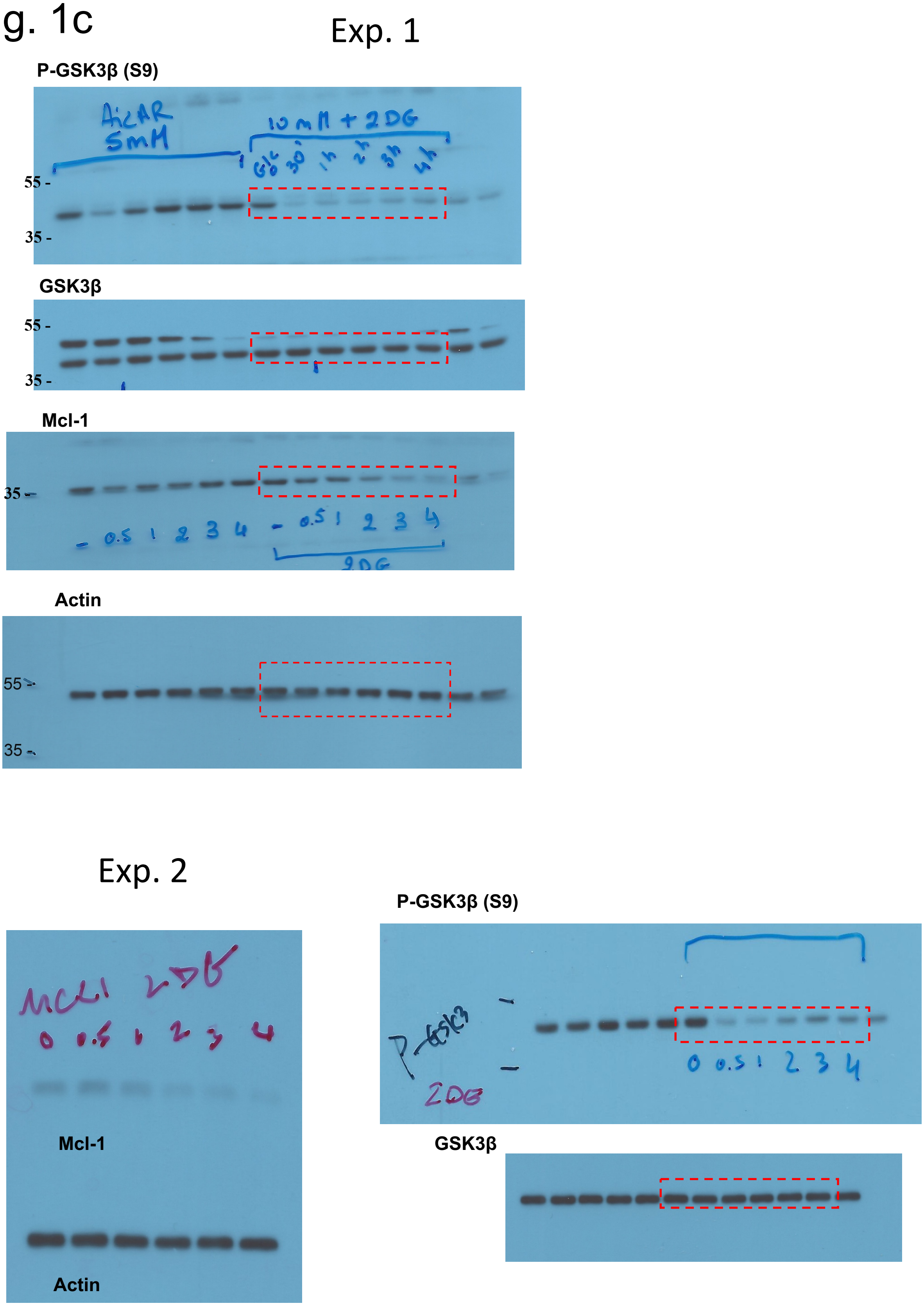
Exp. 1
P-GSK3β (S9)
55 -
35 -
GSK3β
55 -
35 -
Mcl-1
35 -
Actin
55 -
35 -
Exp. 2
Mcl-1
Actin
P-GSK3β (S9)
GSK3β

Fig. 1d

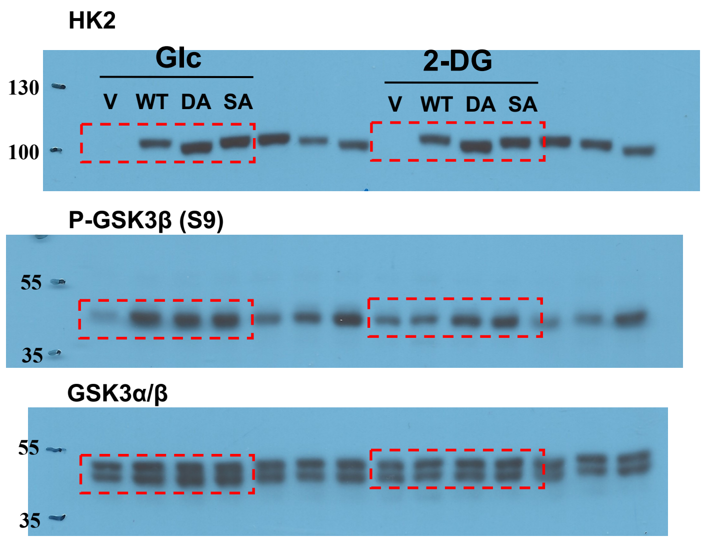

Additional experiments for quantifications

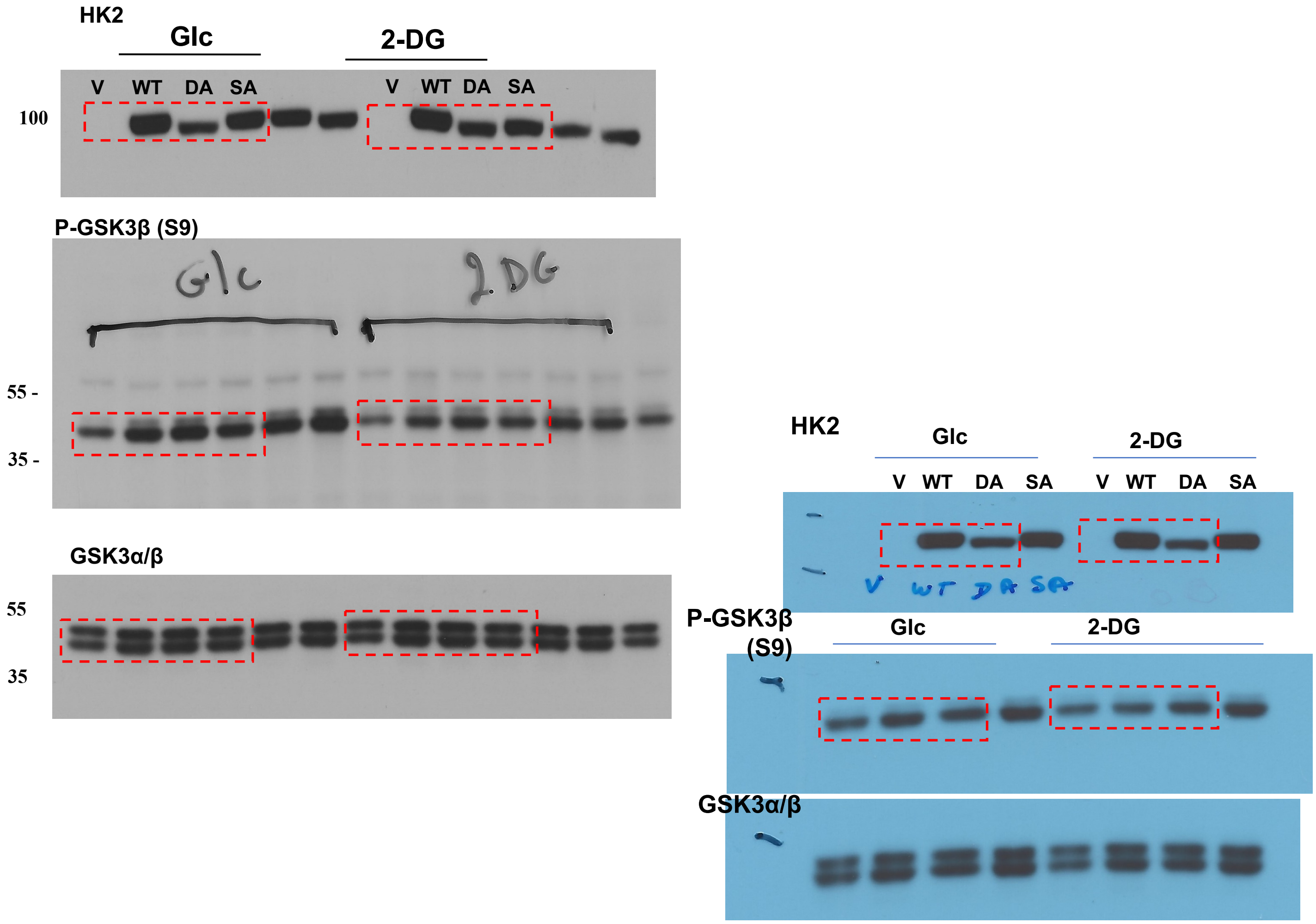

Fig. 1e

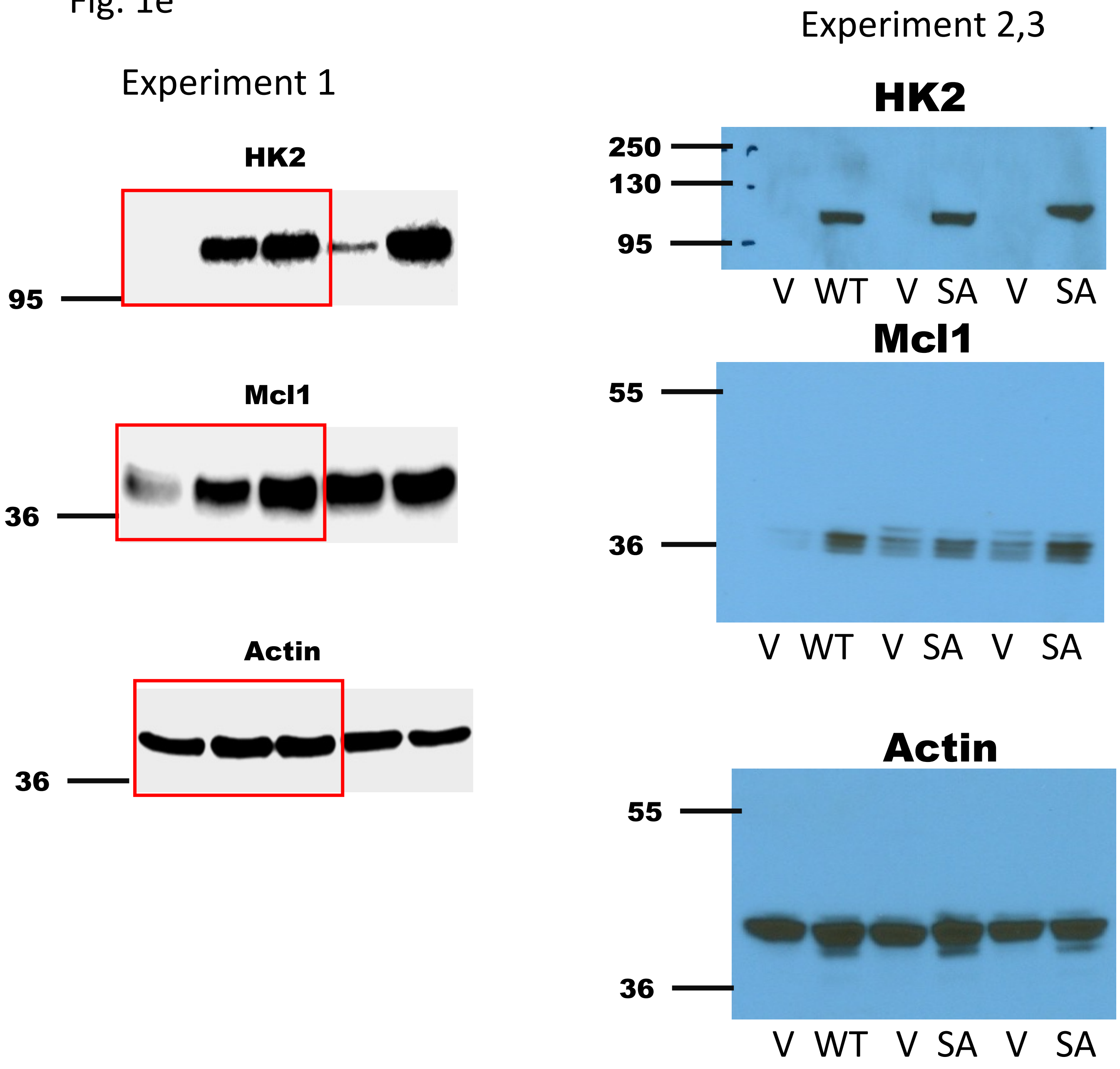

Experiment 1
HK2
95
Mcl1
36
Actin
36
Experiment 2,3
HK2
250
130
95
V WT V SA V SA
Mcl1
55
36
V WT V SA V SA
Actin
55
36
V WT V SA V SA

Fig. 1f

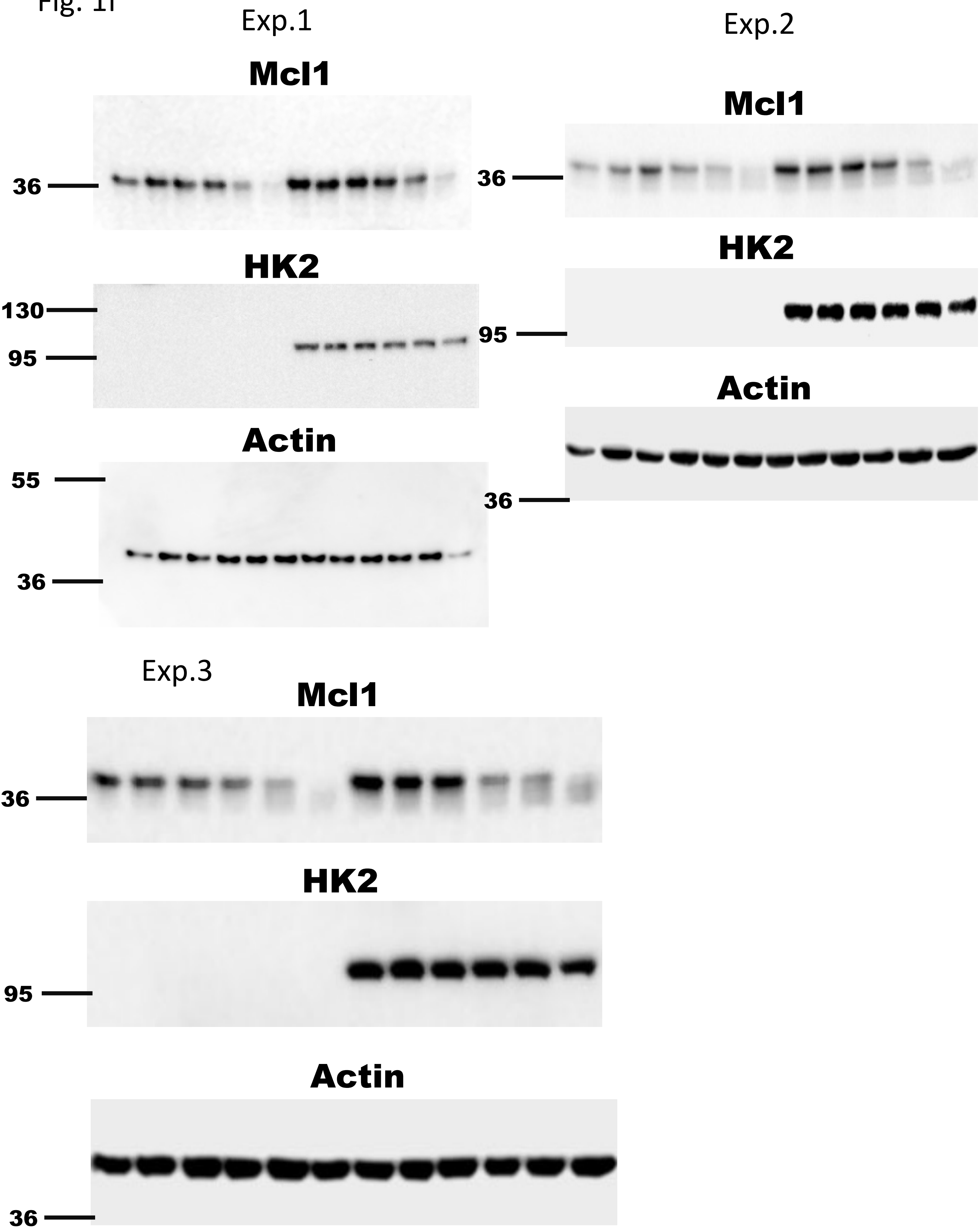
Exp.1
Mcl1
36
HK2
130
95
Actin
55
36
Exp.2
Mcl1
36
HK2
95
Actin
36
Exp.3
Mcl1
36
HK2
95
Actin
36

Fig. 2a

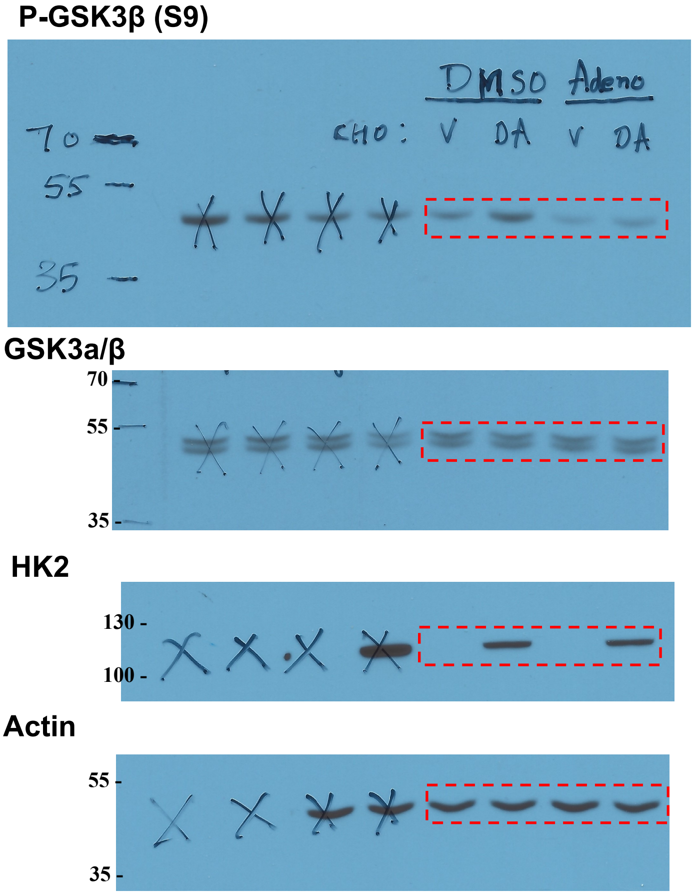

Additional experiment

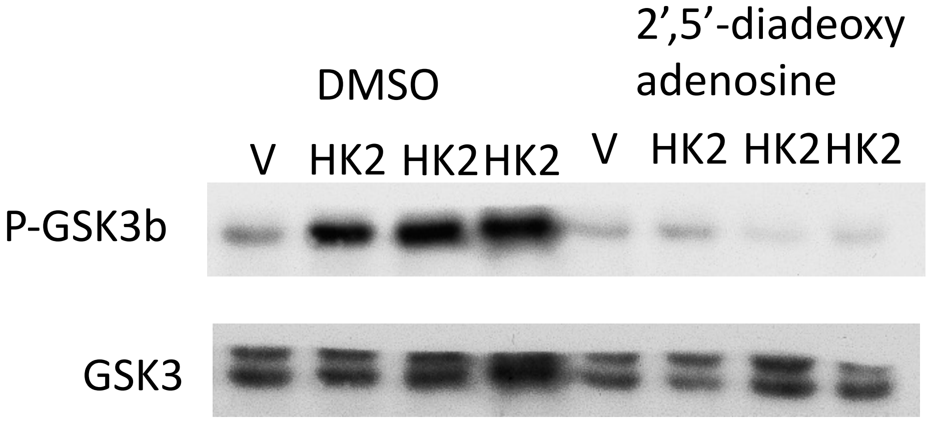

Fig. 2b

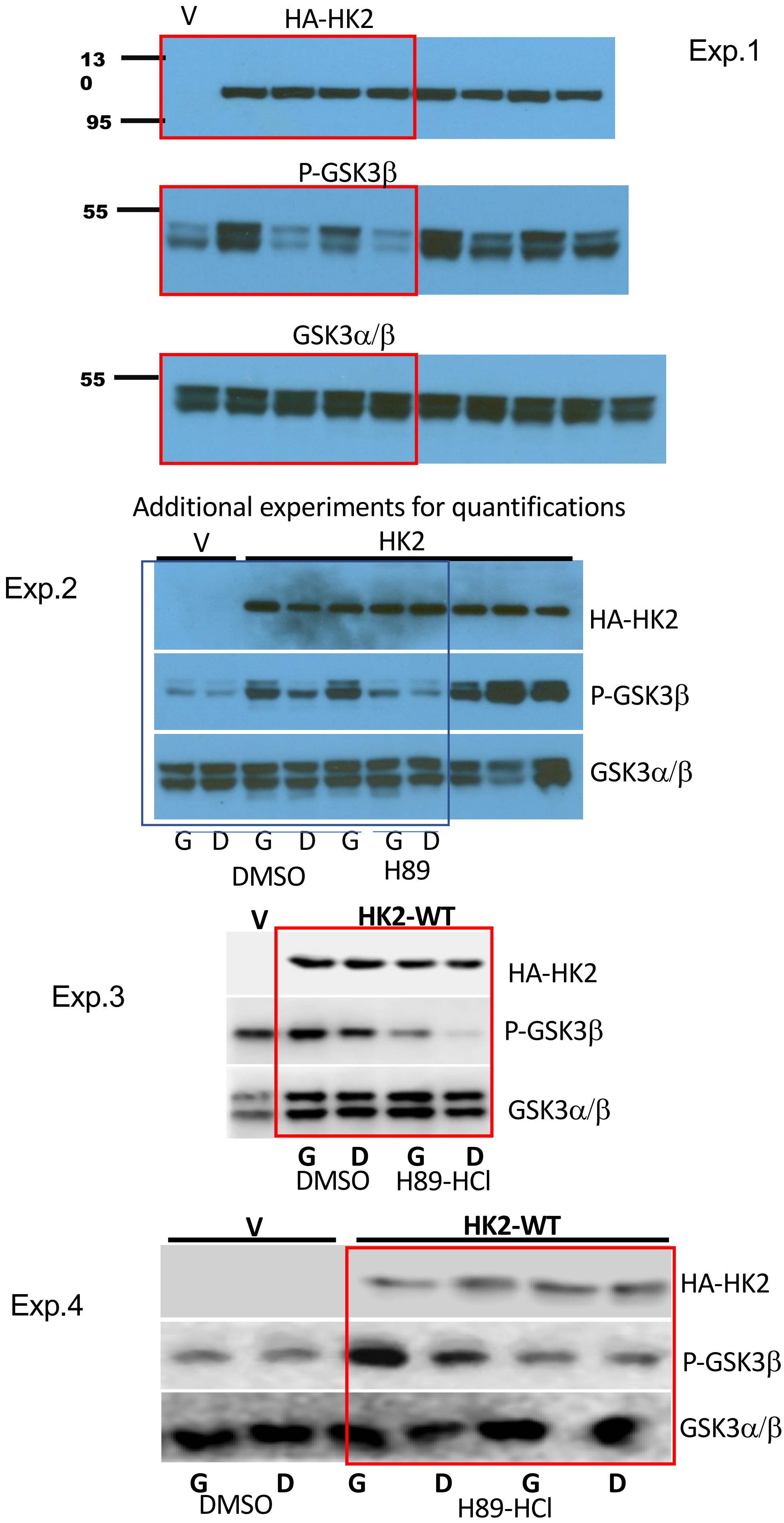
V
HA-HK2
Exp.1
130
95
P-GSK3β
55
GSK3α/β
55
Additional experiments for quantifications
V
HK2
Exp.2
HA-HK2
P-GSK3β
GSK3α/β
G D G D G G D
DMSO
H89
V
HK2-WT
Exp.3
HA-HK2
P-GSK3β
GSK3α/β
G D G D
DMSO H89-HCl
V
HK2-WT
Exp.4
HA-HK2
P-GSK3β
GSK3α/β
G D G D G D
DMSO
H89-HCl

Fig. 2c

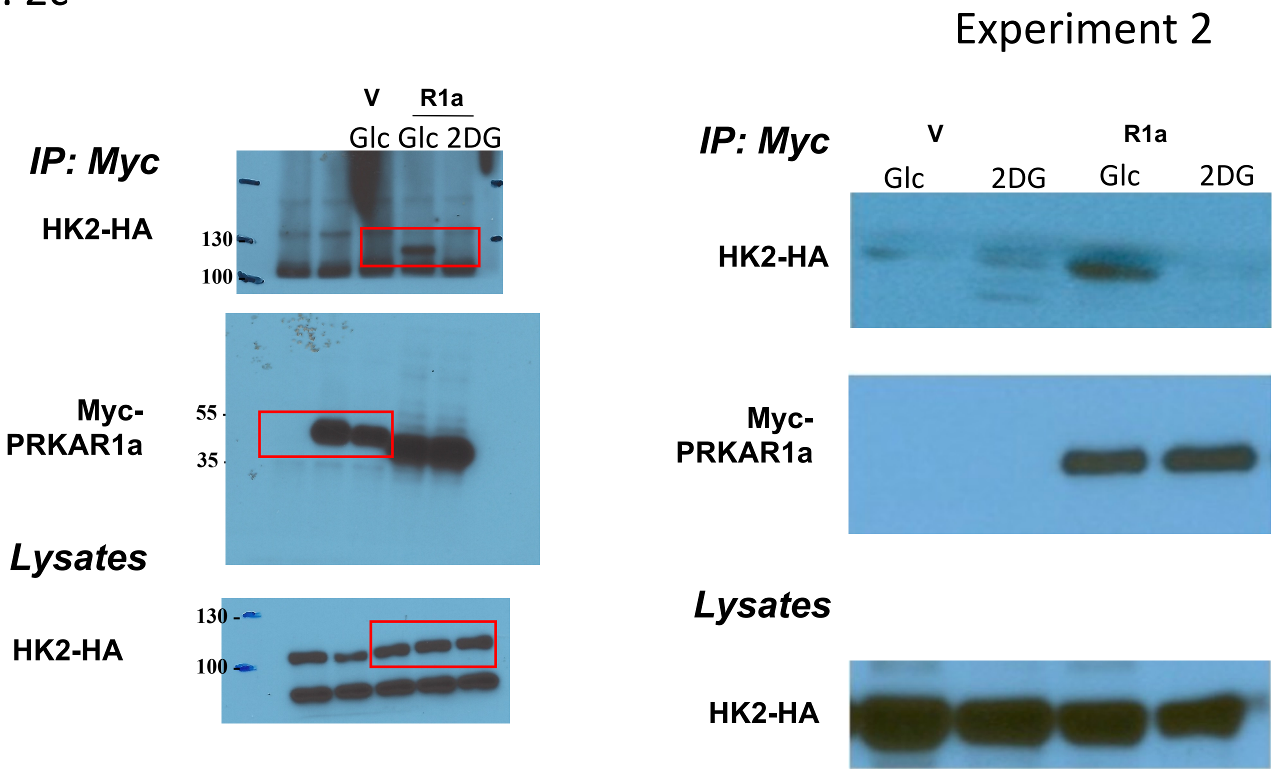

Experiment 2
V
R1a
Glc Glc 2DG
IP: Myc
HK2-HA
130
100
Myc-
PRKAR1a
55
35
Lysates
HK2-HA
130
100
IP: Myc
V
R1a
Glc
2DG
Glc
2DG
HK2-HA
Myc-
PRKAR1a
Lysates
HK2-HA

Fig. 2d

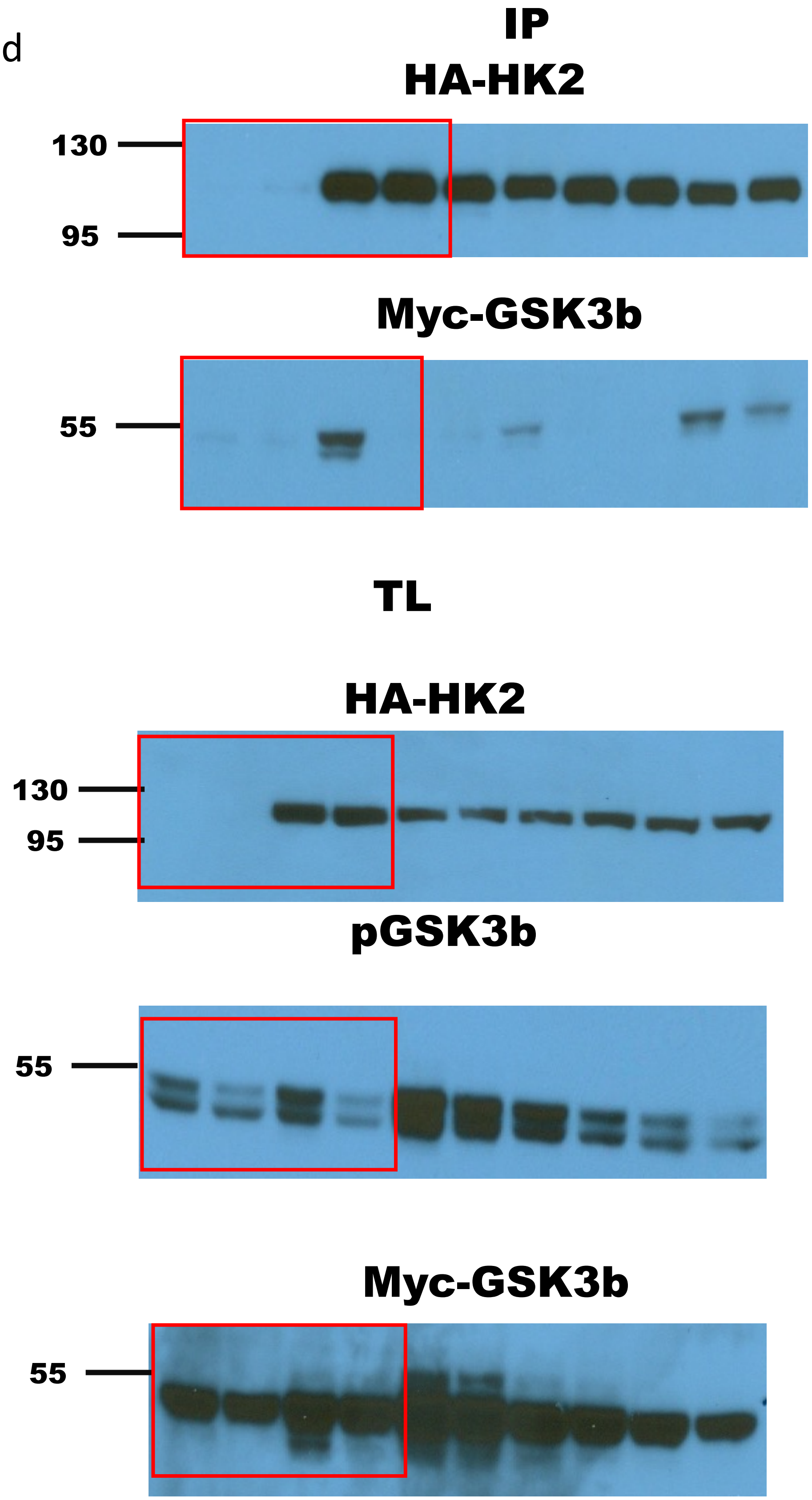

IP
HA-HK2
130
95
Myc-GSK3b
55
TL
HA-HK2
130
95
pGSK3b
55
Myc-GSK3b
55

Fig. 2d (experiment 2)

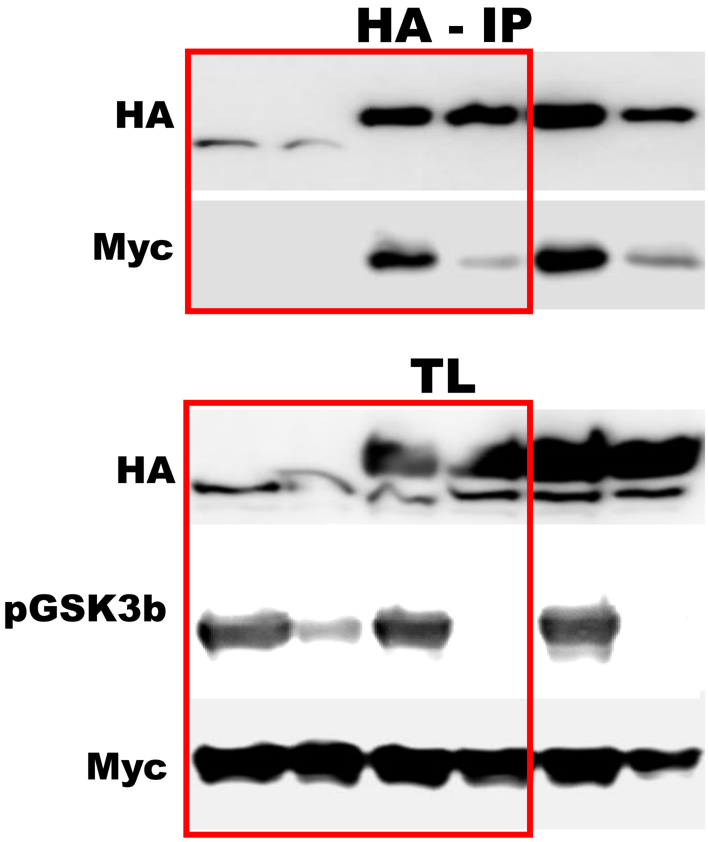

Fig. 2e

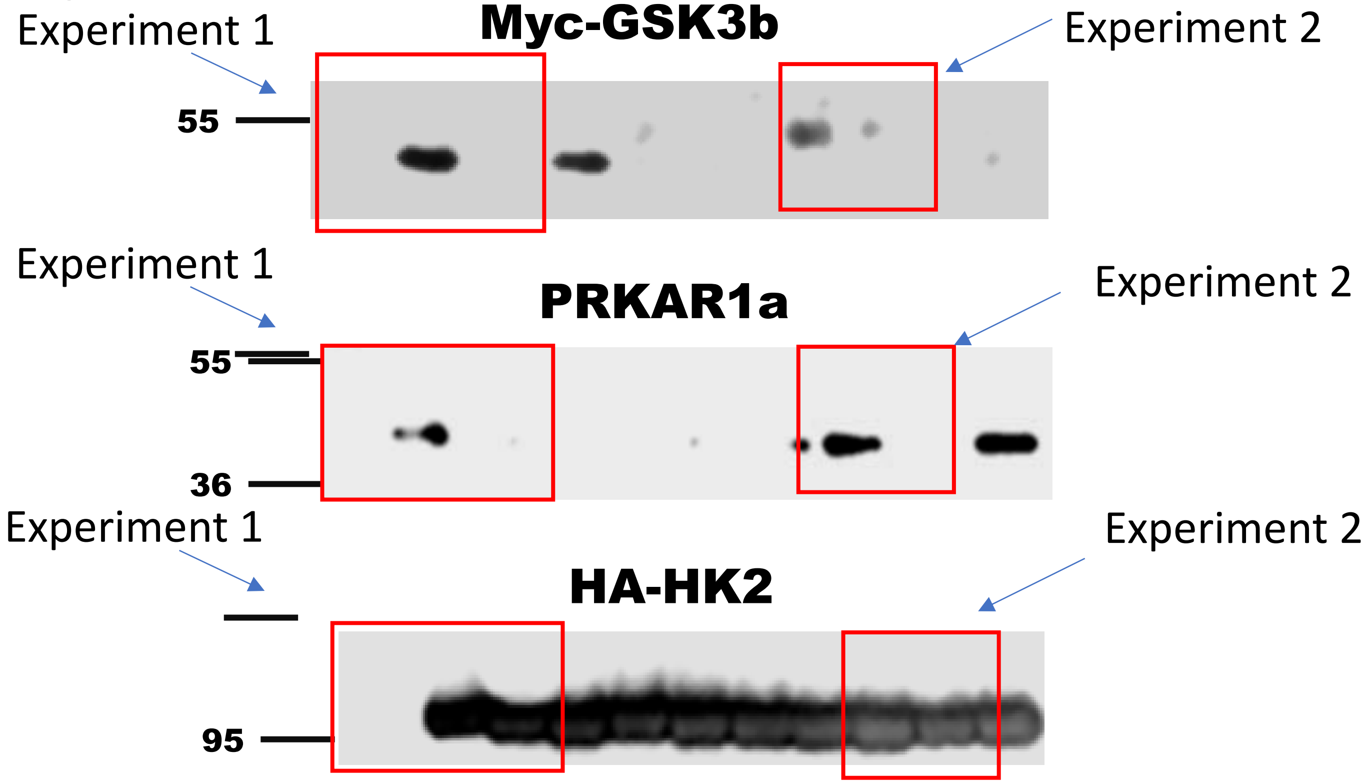

Experiment 1
Myc-GSK3b
Experiment 2
55
Experiment 1
PRKAR1a
Experiment 2
55
36
Experiment 1
HA-HK2
Experiment 2
95

Fig. 2f

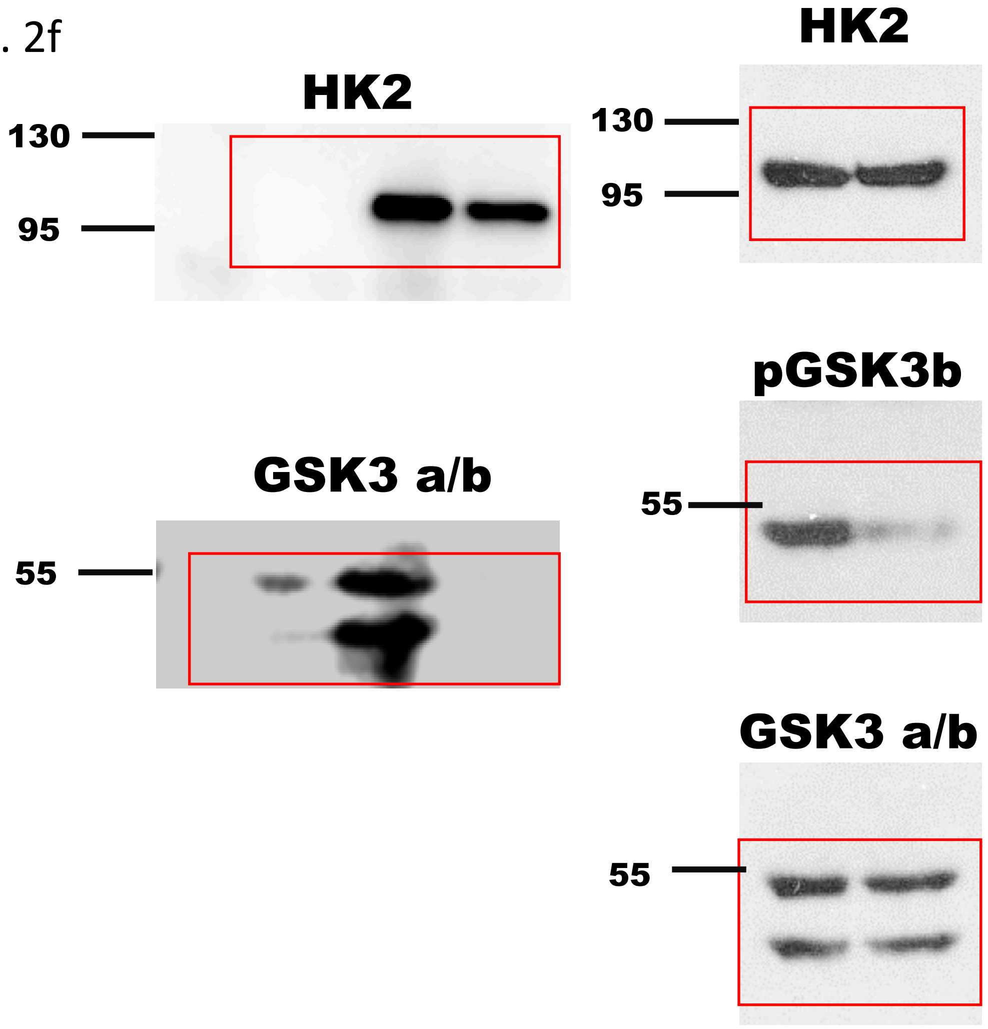

HK2
130
95
HK2
130
95
pGSK3b
55
GSK3 a/b
55
GSK3 a/b
55

Fig. 2g

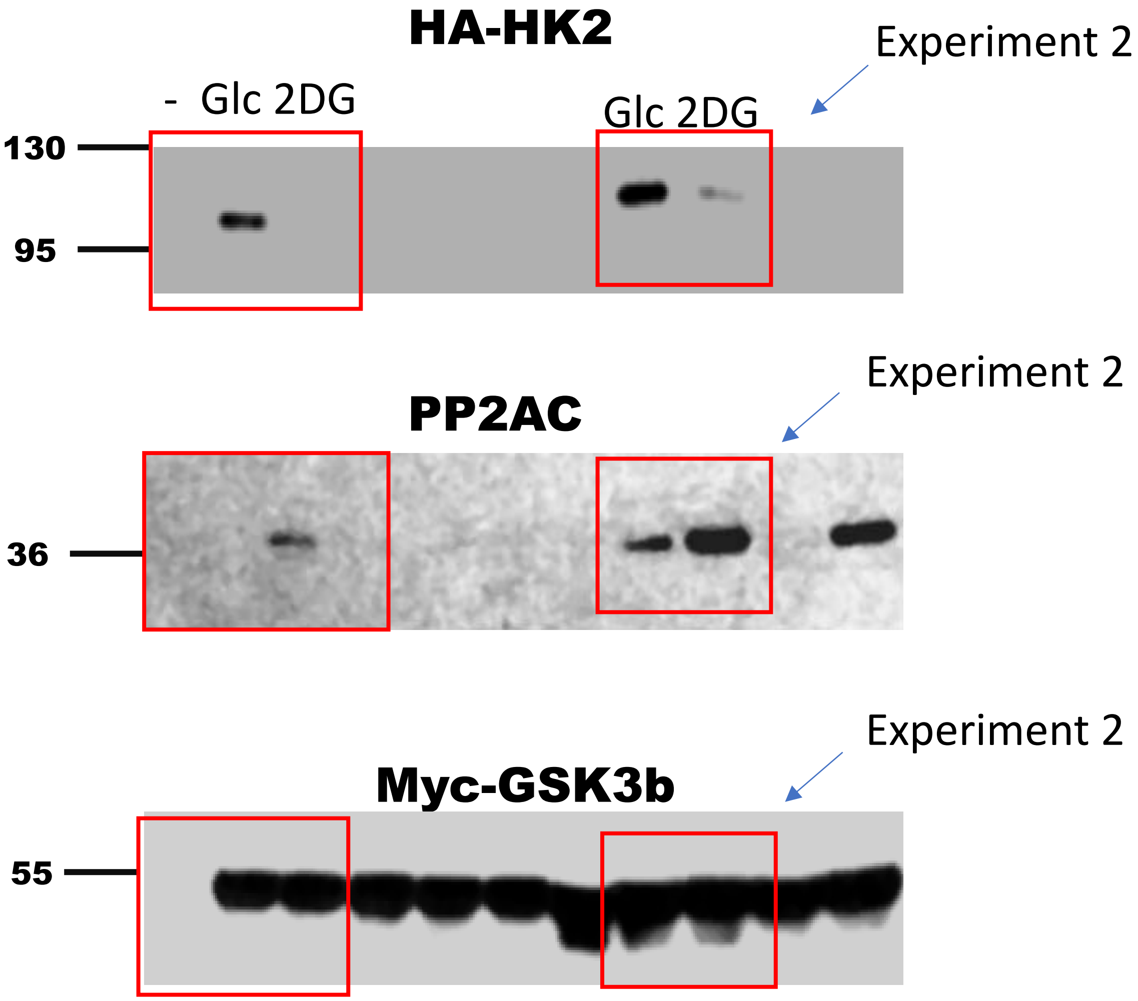
HA-HK2
Experiment 2
- Glc 2DG
Glc 2DG
130
95
PP2AC
Experiment 2
36
Myc-GSK3b
Experiment 2
55

Fig. 5a

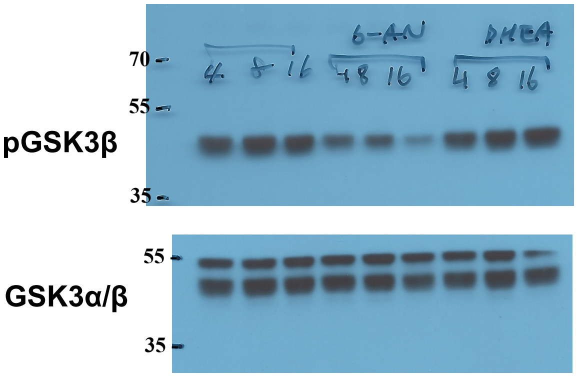

Additional experiments used for quantifications

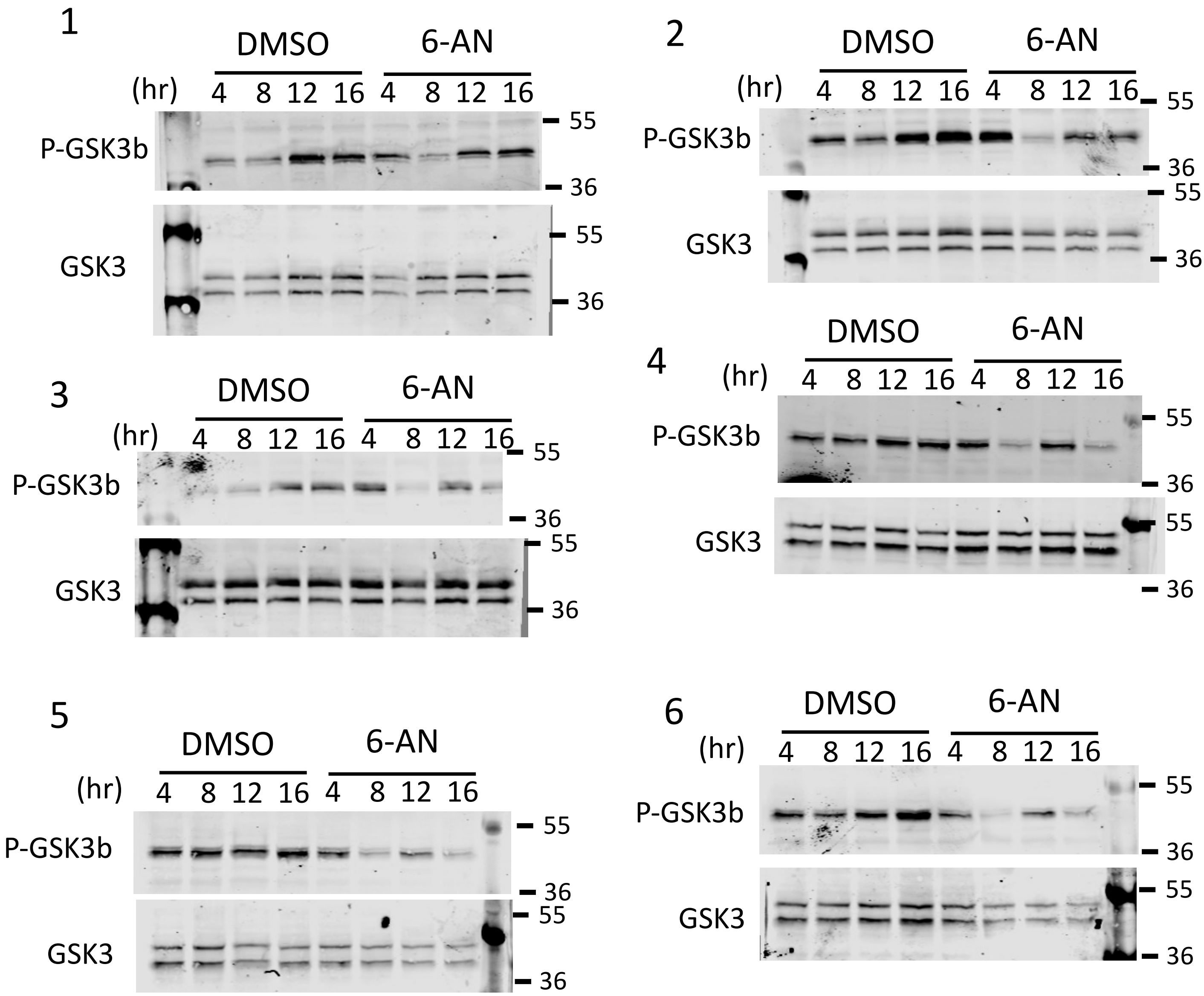

Fig. 5d

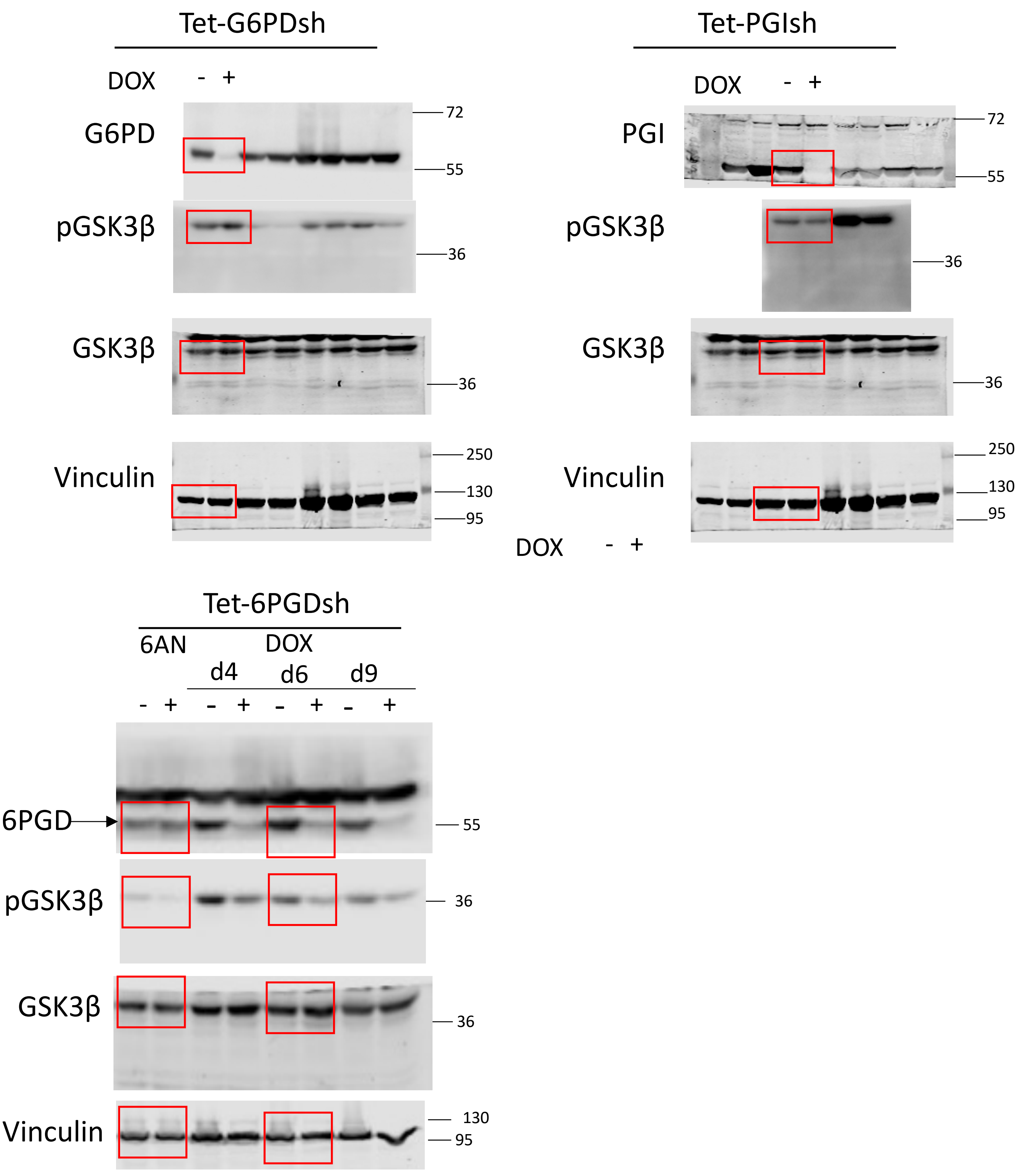
Tet-G6PDsh
DOX - +
G6PD
72
55
pGSK3β
36
GSK3β
36
Vinculin
250
130
95
Tet-PGIsh
DOX - +
PGI
72
55
pGSK3β
36
GSK3β
36
Vinculin
250
130
95
DOX - +
Tet-6PGDsh
6AN
DOX
d4
d6
d9
- + - + - + - +
6PGD
55
pGSK3β
36
GSK3β
36
Vinculin
130
95

Fig. 5g

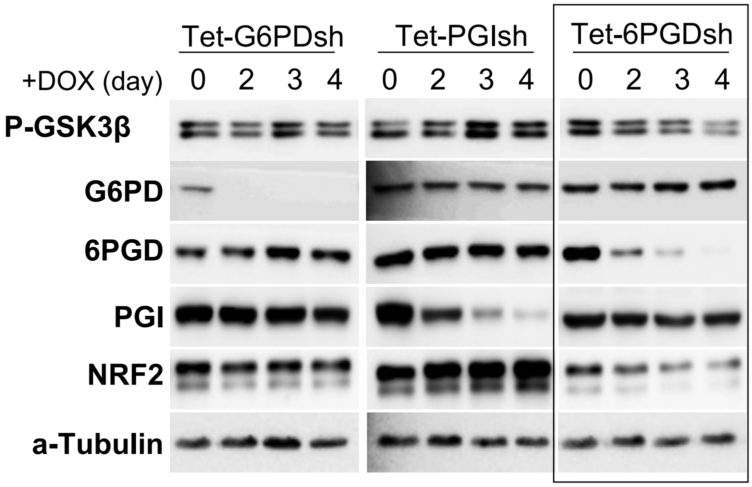

Additional experiments

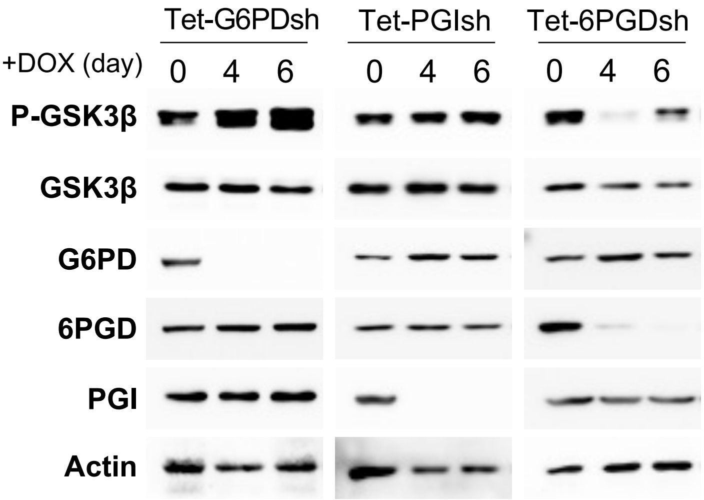

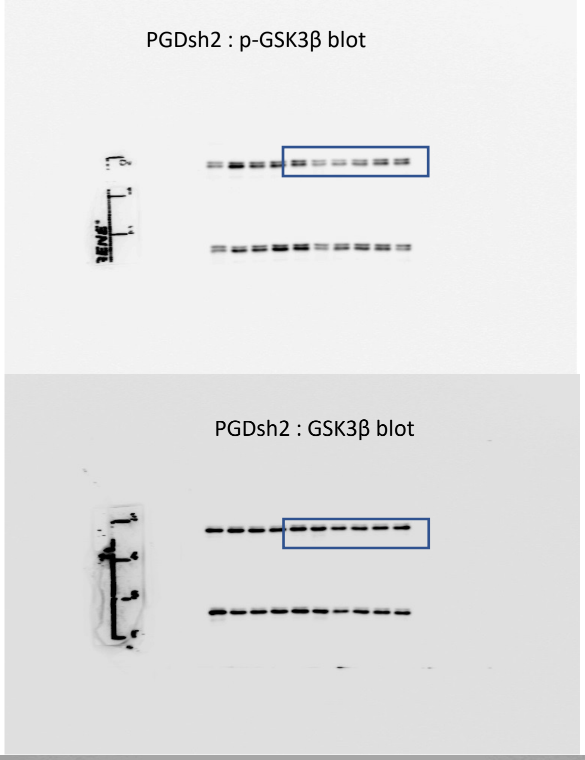

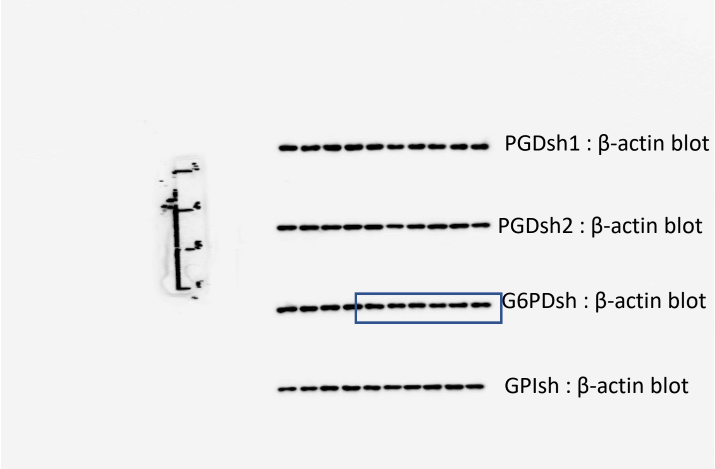

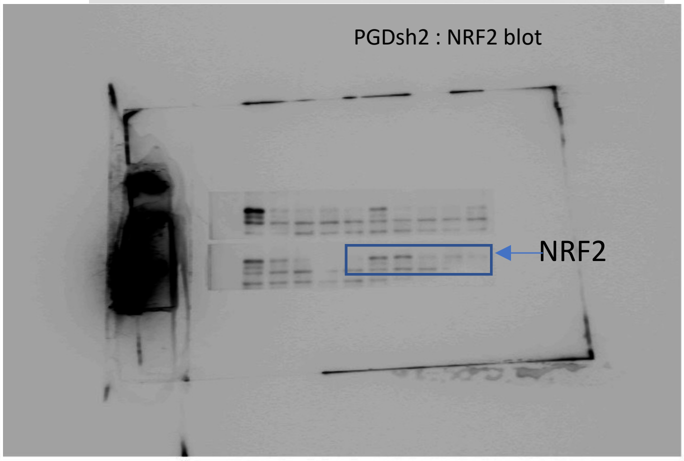

Fig. 6a

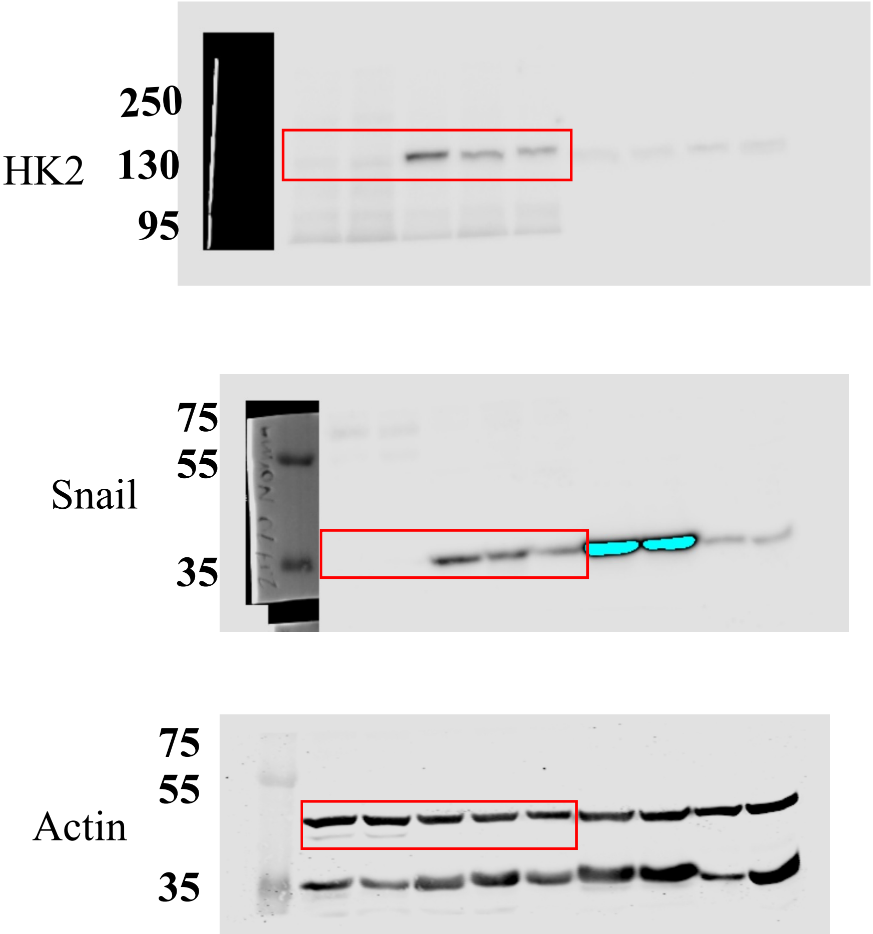

Fig. 7d

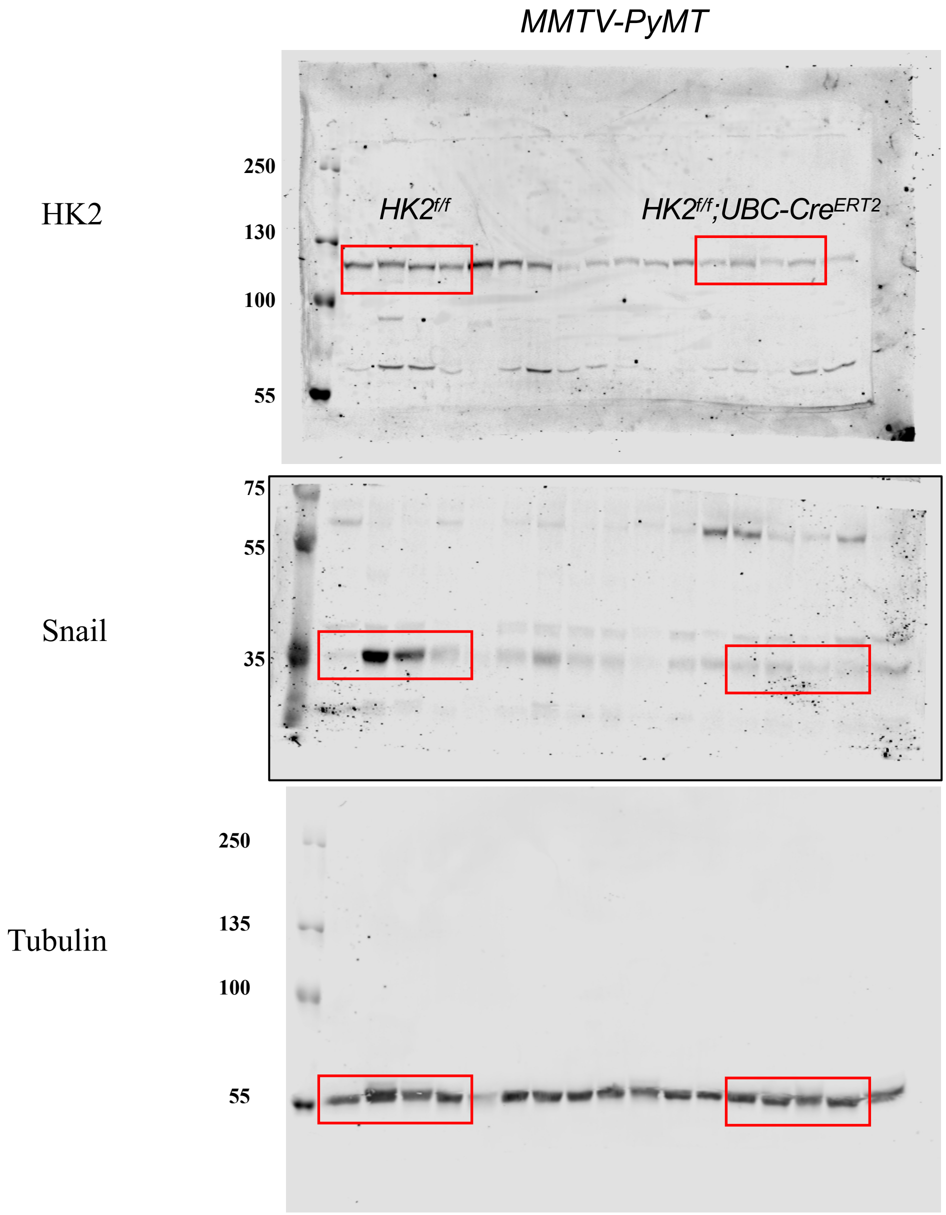

MMTV-PyMT
HK2
250
130
100
55
HK2f/f
HK2f/f;UBC-CreERT2
Snail
75
55
35
Tubulin
250
135
100
55

Fig. 7e

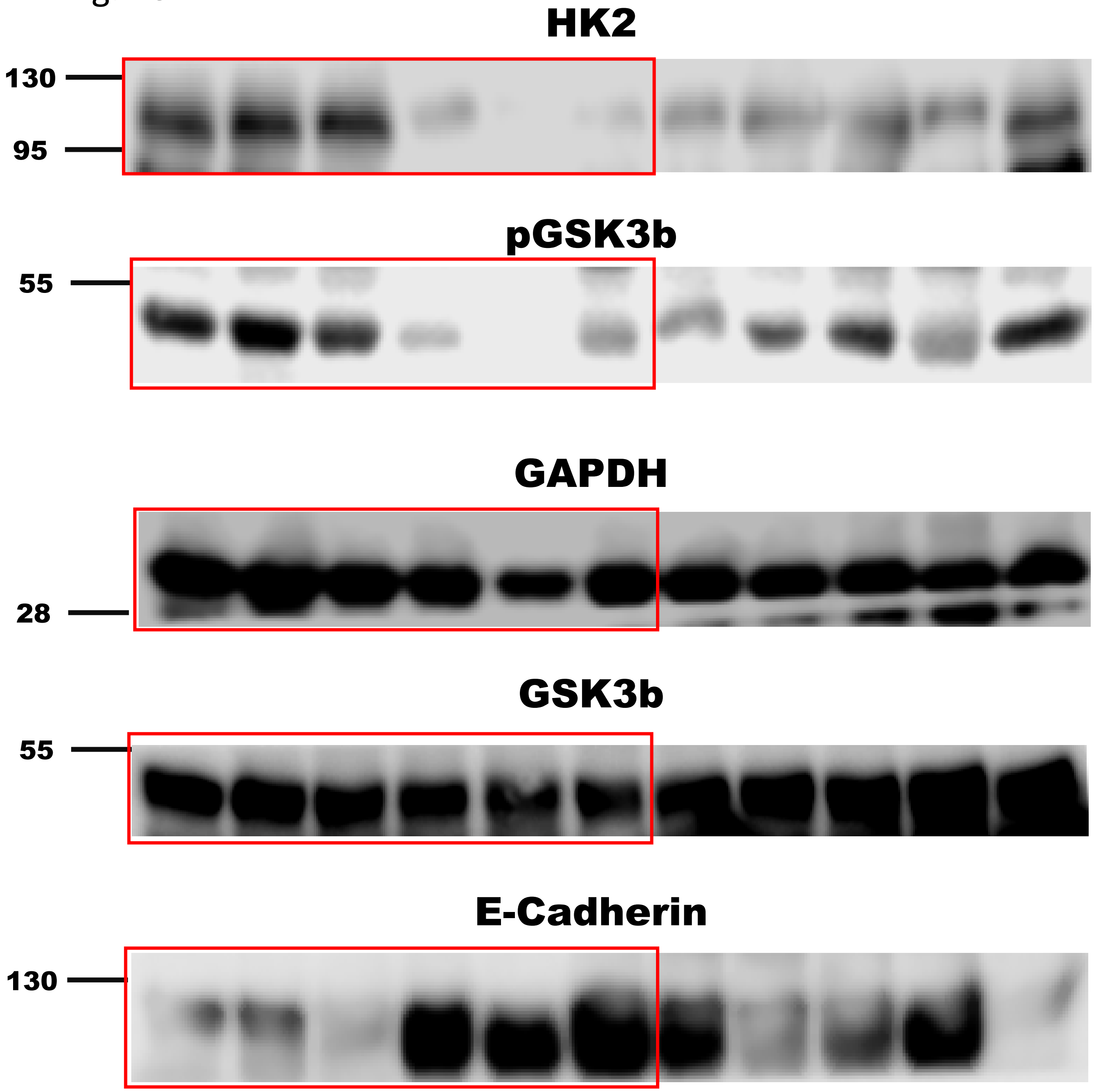

Fig. 7f

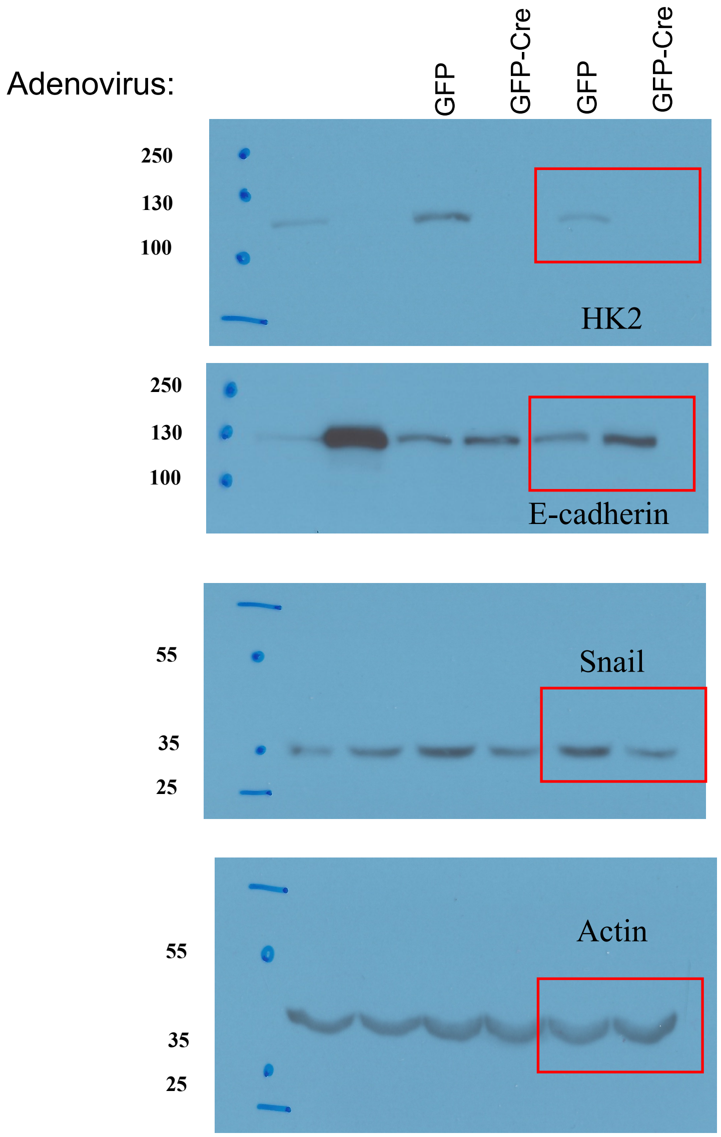
Adenovirus:
GFP
GFP-Cre
GFP
GFP-Cre
250
130
100
HK2
250
130
100
E-cadherin
55
35
25
Snail
55
35
25
Actin

Fig. 8a

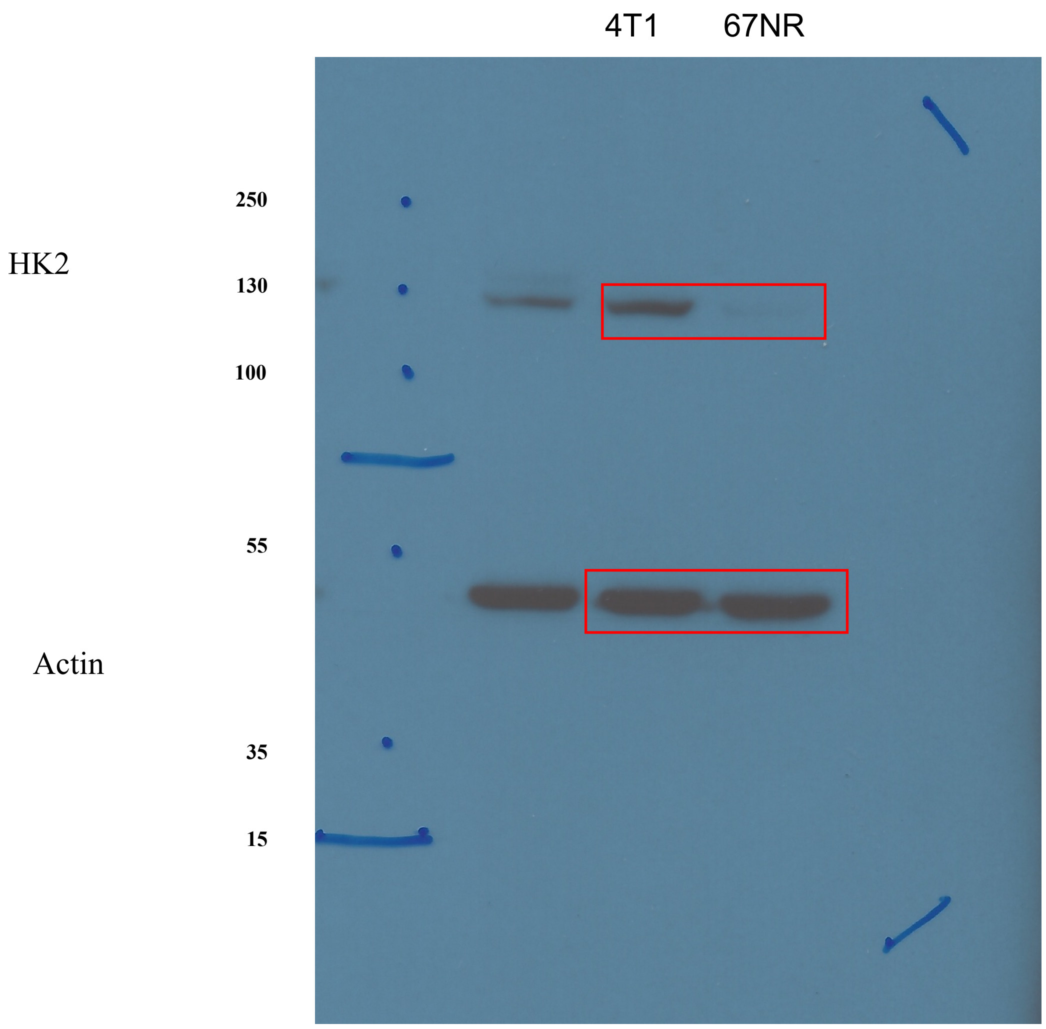

Fig. 8c +8d

c

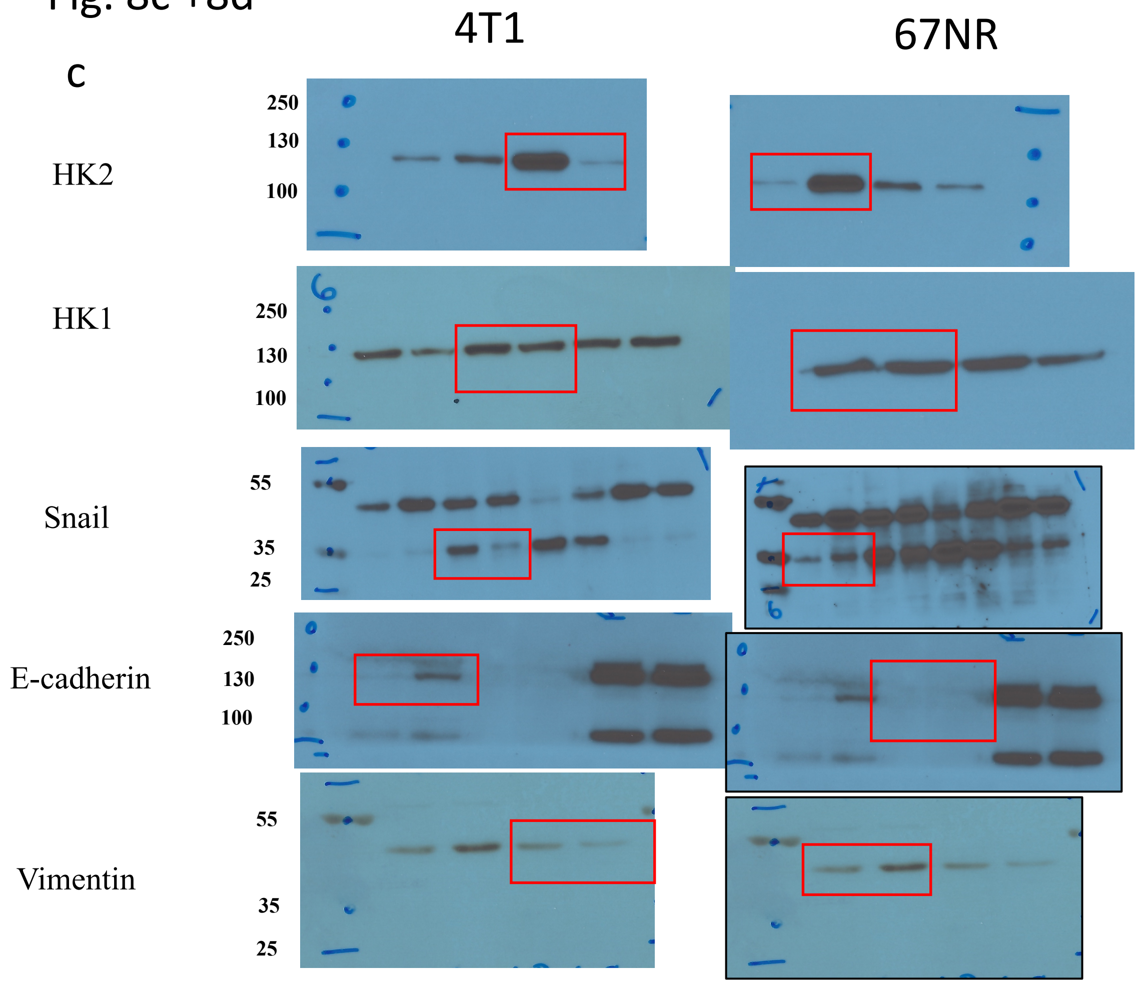

d (additional Immunoblots used for quantification)

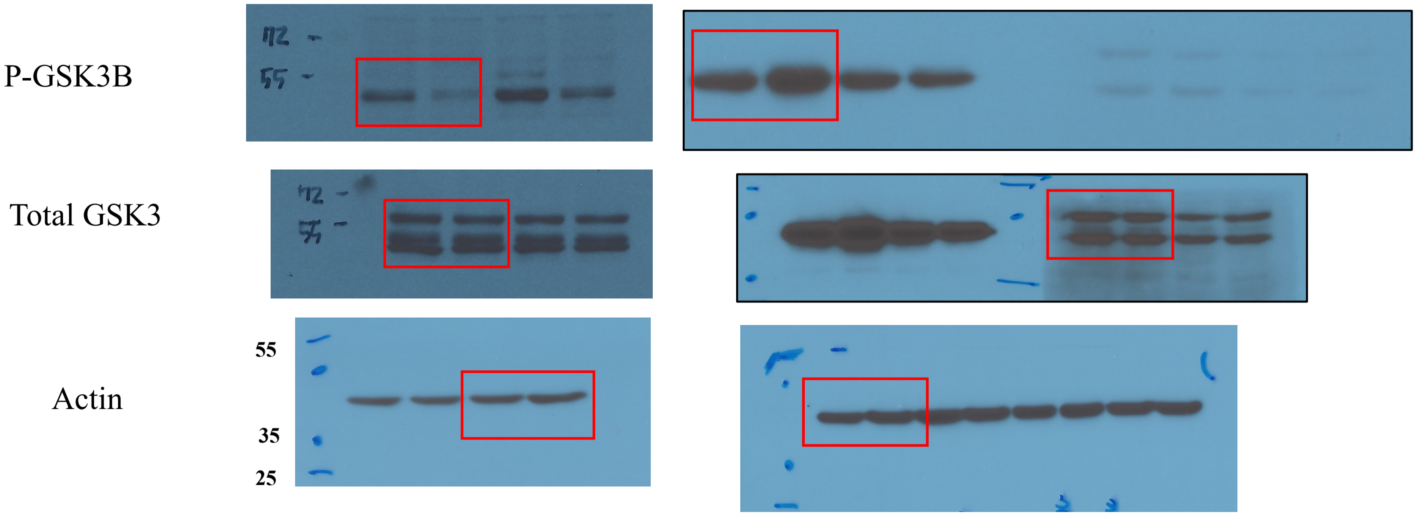

Fig. 8d (additional experiments)

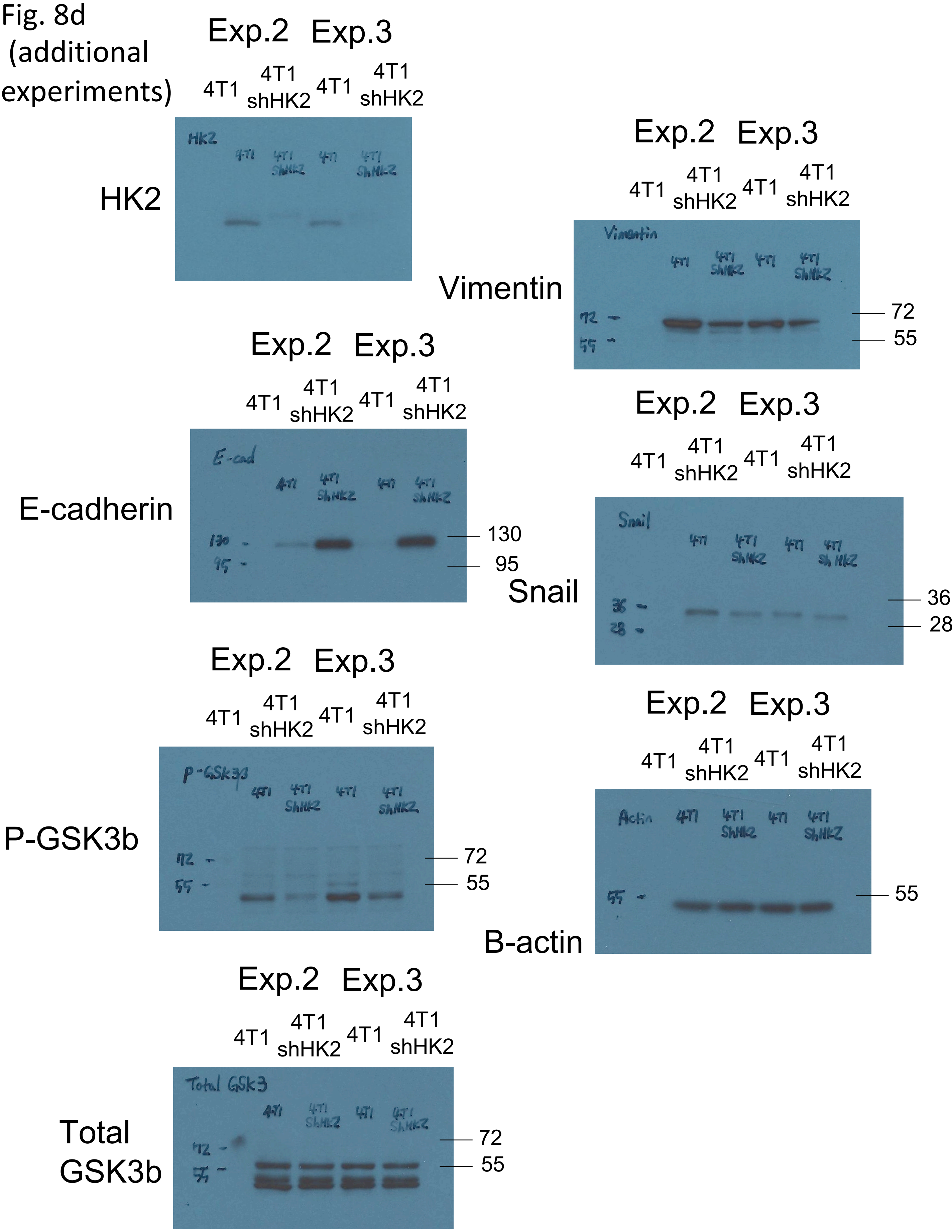

Fig. 8d
(additional
experiments)

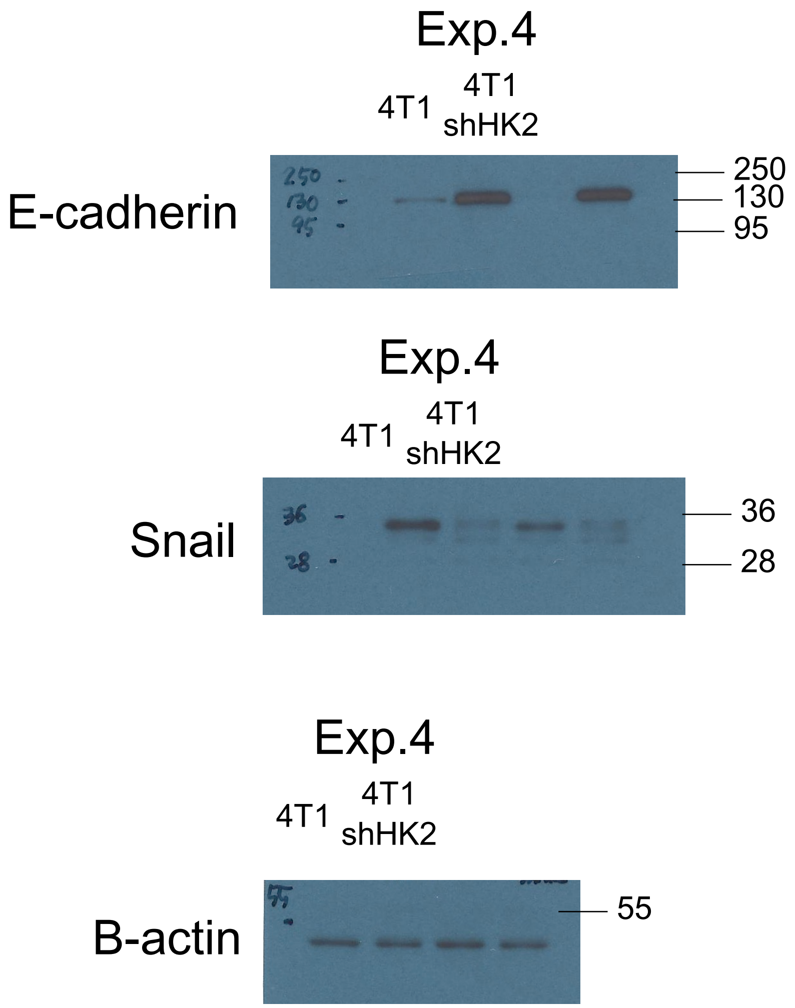

Fig. 9a

Snail

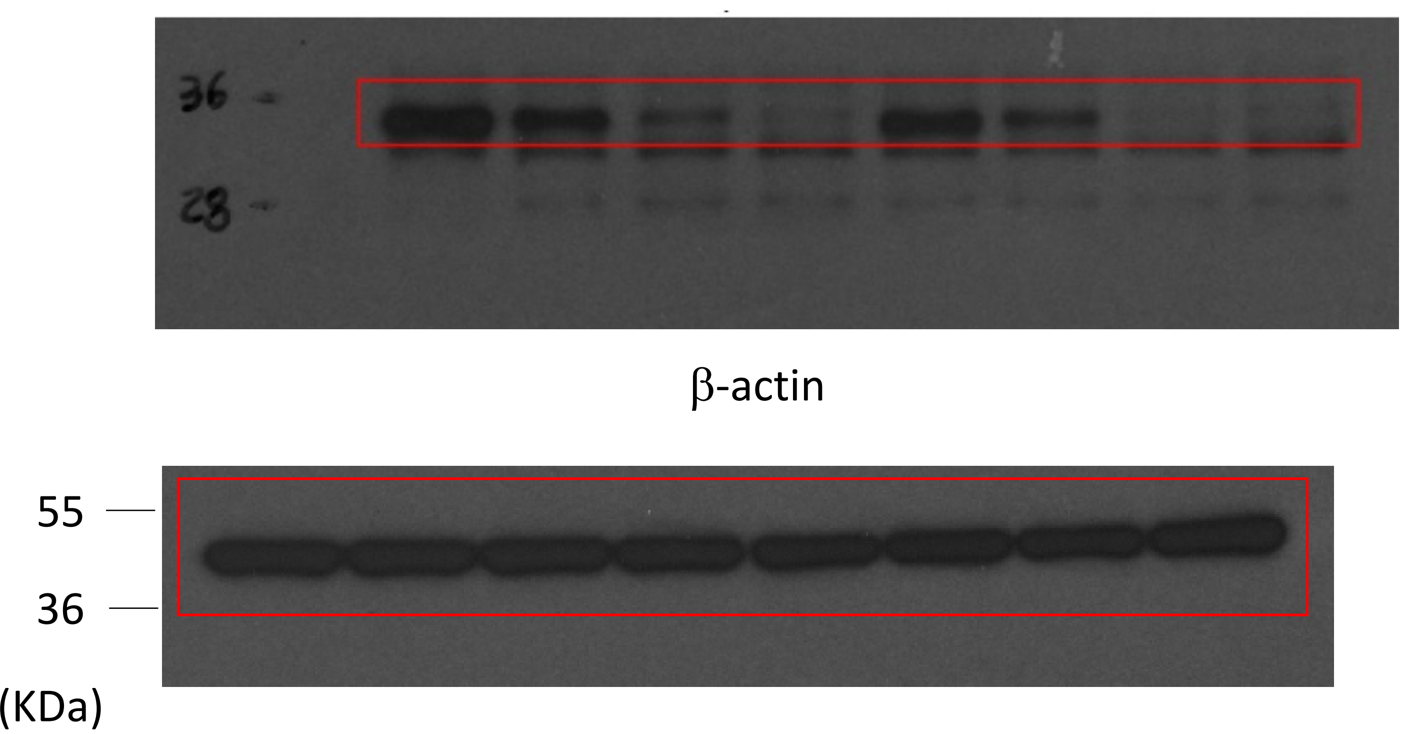

Fig. 9b (additional experiments)

Exp.2

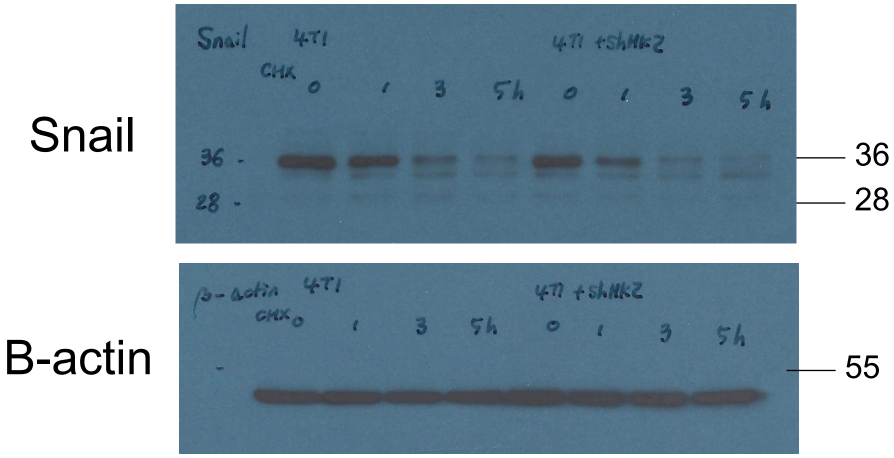

Fig. 9a (additional experiments)

Exp.3

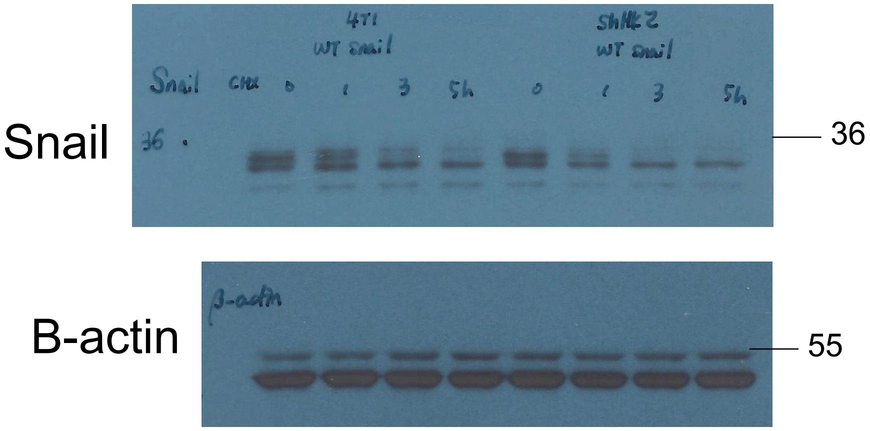

Exp.4

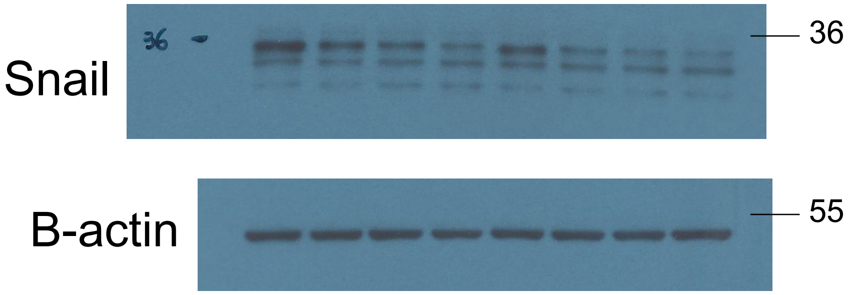

Fig.9b

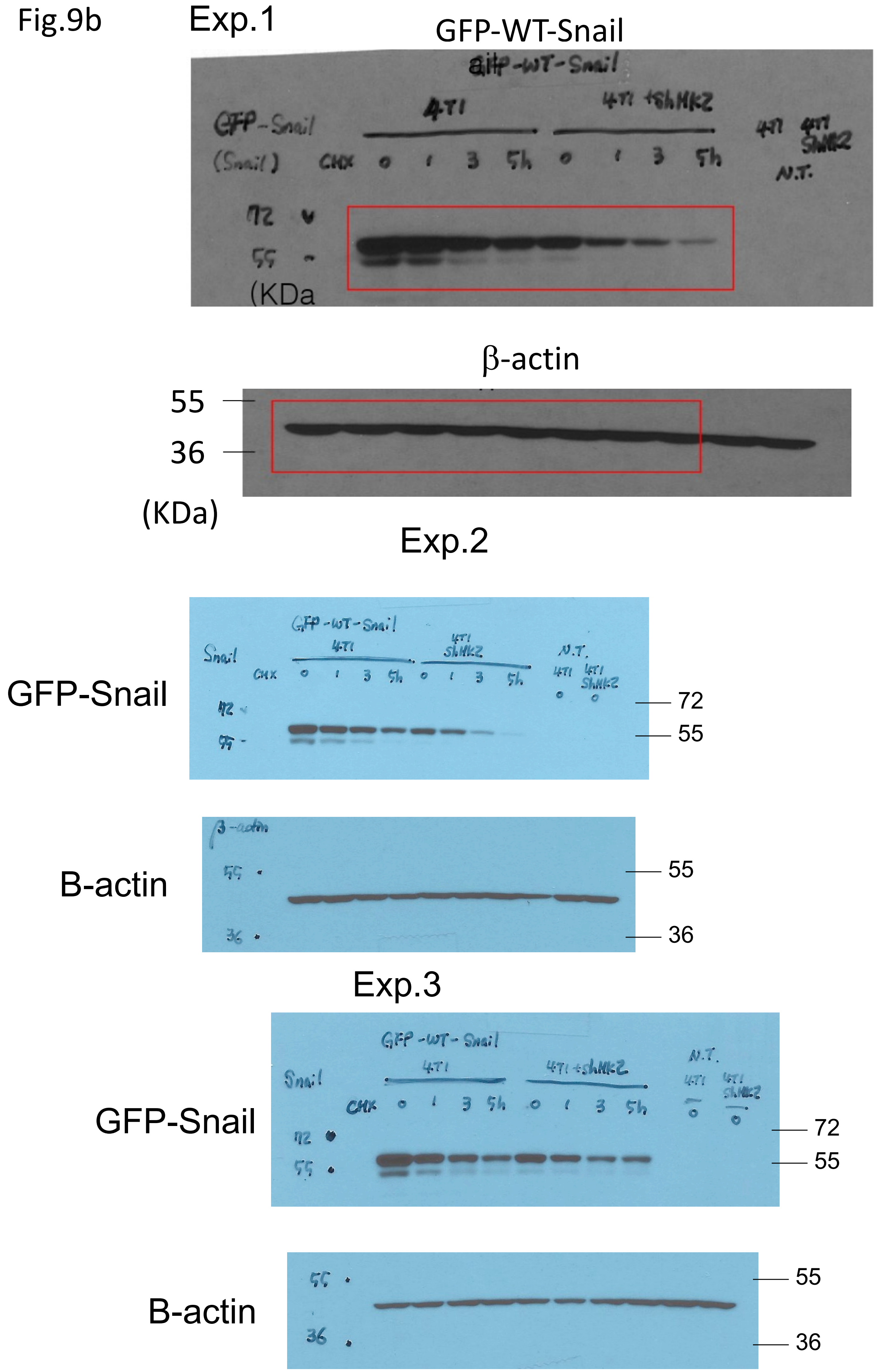
Exp.1
GFP-WT-Snail
β-actin
55
36
(KDa)
Exp.2
GFP-Snail
72
55
B-actin
55
36
Exp.3
GFP-Snail
72
55
B-actin
55
36

Fig. 9c

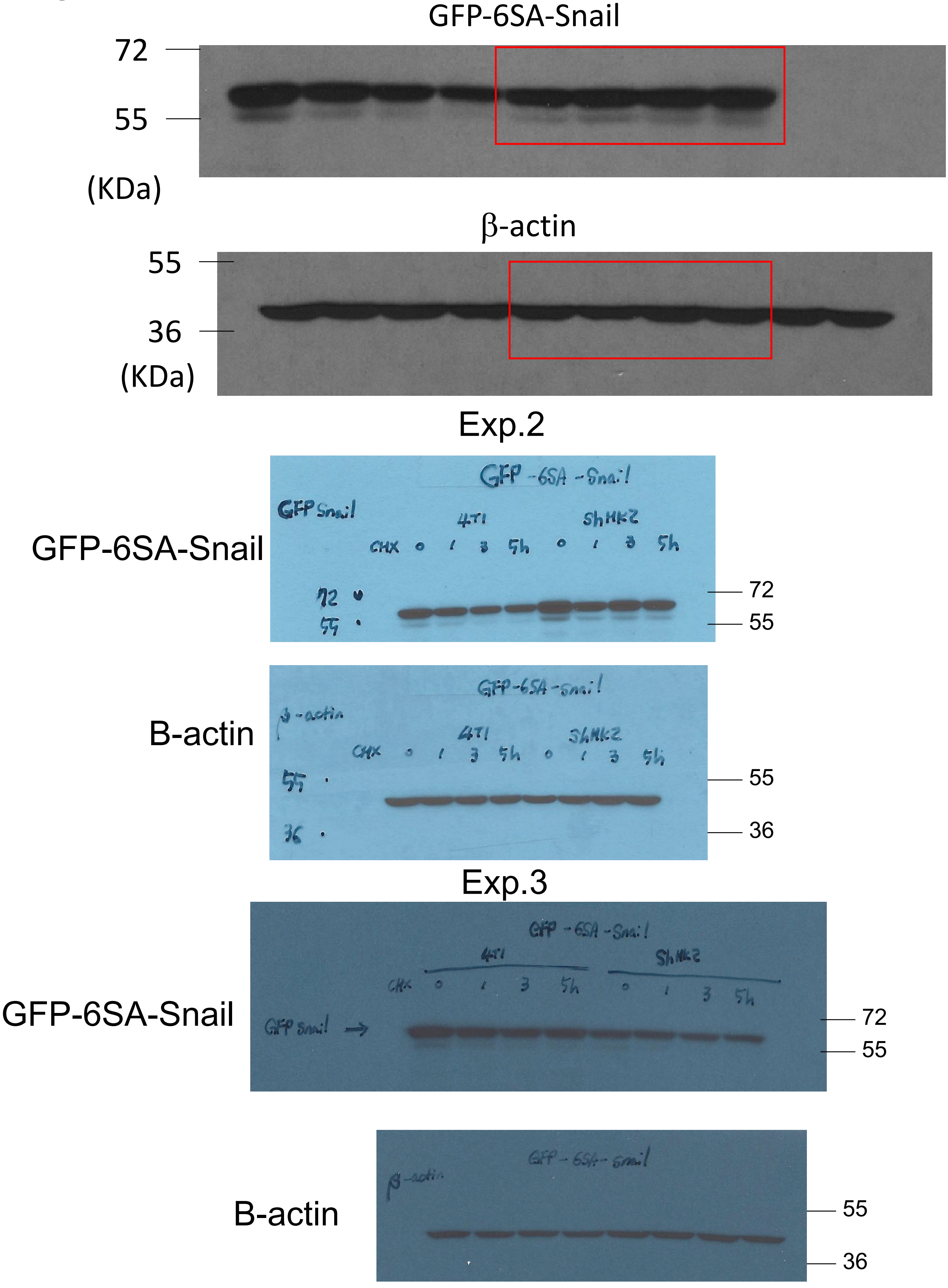
Exp.1
GFP-6SA-Snail
72
55
(KDa)
β-actin
55
36
(KDa)
Exp.2
GFP-6SA-Snail
GFP-6SA-Snail
GFP Snail
4T1
ShMK2
CHX 0 1 3 5h 0 1 3 5h
72
55
72
55
B-actin
GFP-6SA-Snail
β-actin
4T1
ShMK2
CHX 0 1 3 5h 0 1 3 5h
55
36
55
36
Exp.3
GFP-6SA-Snail
GFP-6SA-Snail
4T1
ShMK2
CHX 0 1 3 5h 0 1 3 5h
GFP Snail →
72
55
B-actin
GFP-6SA-Snail
β-actin
55
36

Fig. 10a

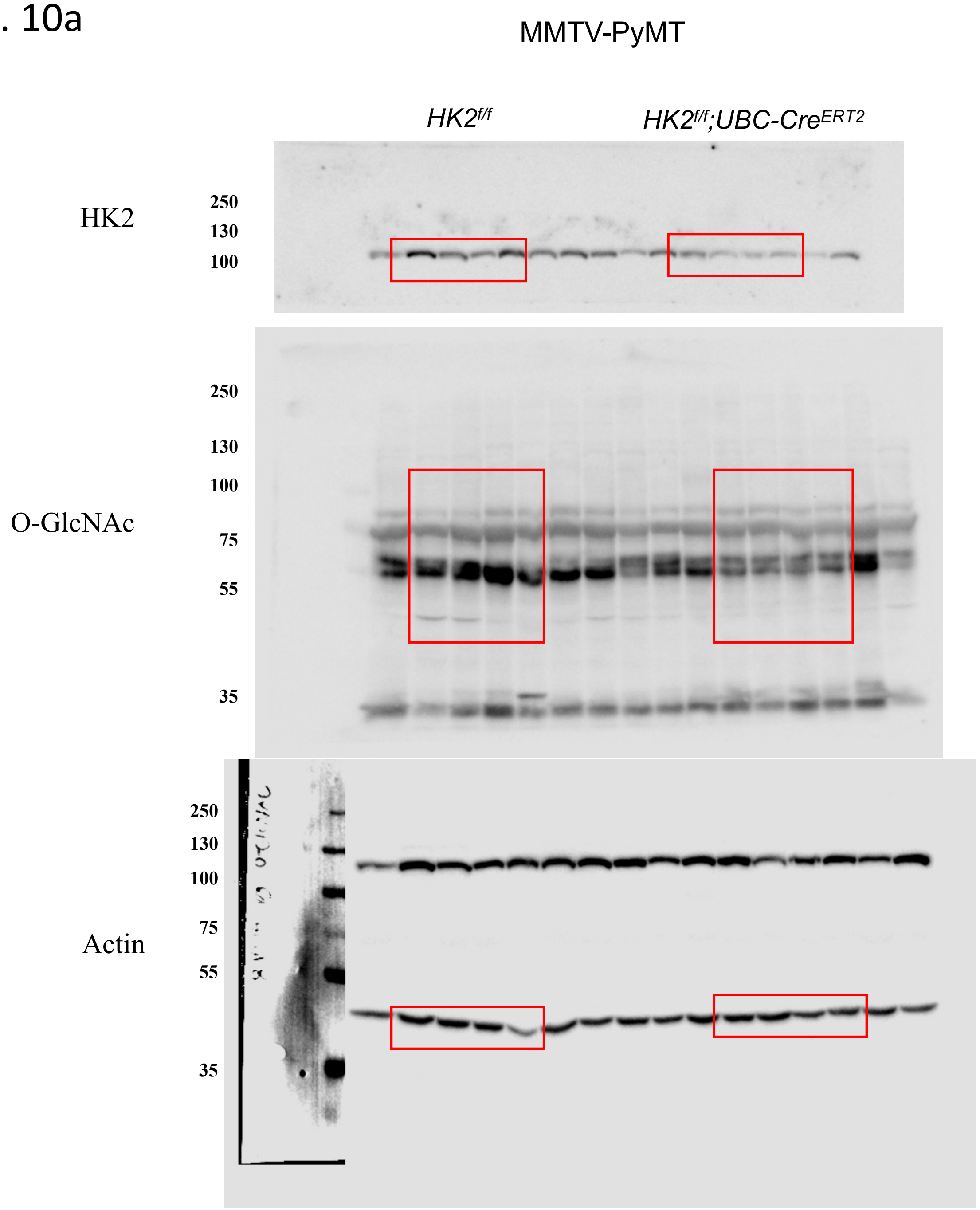
MMTV-PyMT
HK2f/f
HK2f/f;UBC-CreERT2
HK2
250
130
100
O-GlcNAc
250
130
100
75
55
35
Actin
250
130
100
75
55
35

Fig. 10b

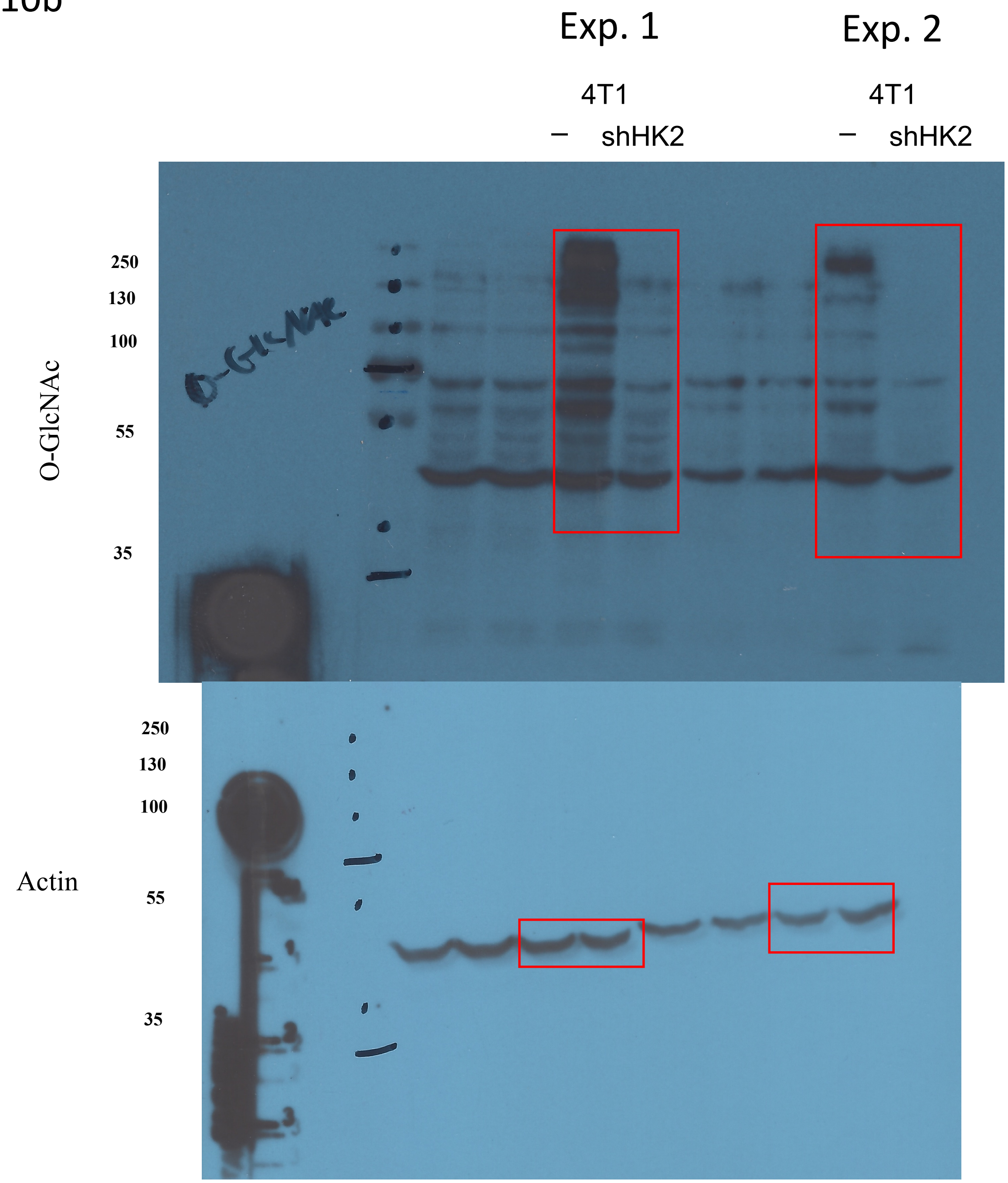
Exp. 1
Exp. 2
4T1
–
shHK2
4T1
–
shHK2
O-GlcNAc
250
130
100
55
35
Actin
250
130
100
55
35

Fig. 10b

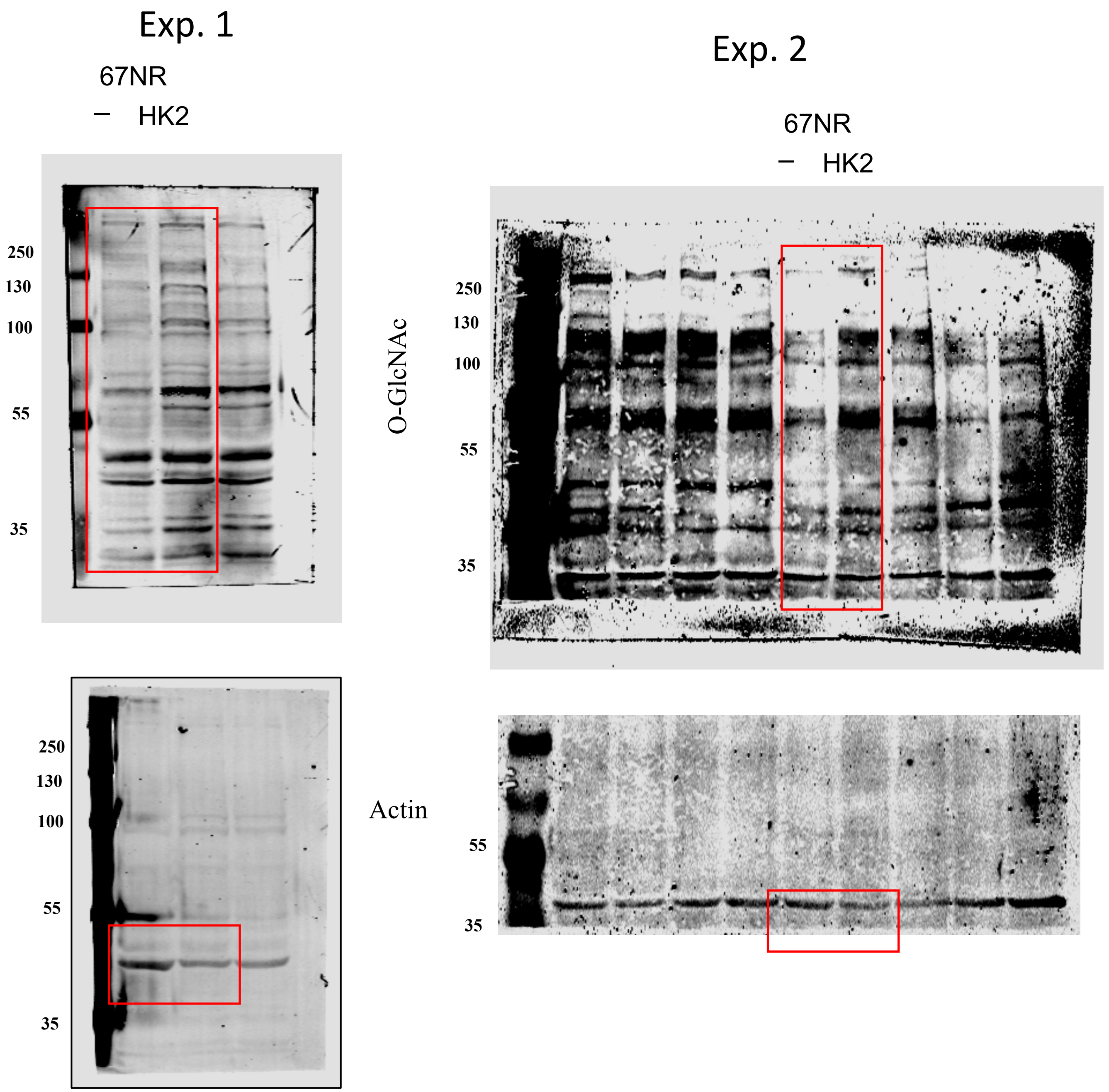

Exp. 1
67NR
– HK2
250
130
100
55
35
O-GlcNAc
250
130
100
55
35
Actin
Exp. 2
67NR
– HK2
250
130
100
55
35
55
35

Fig. 10d

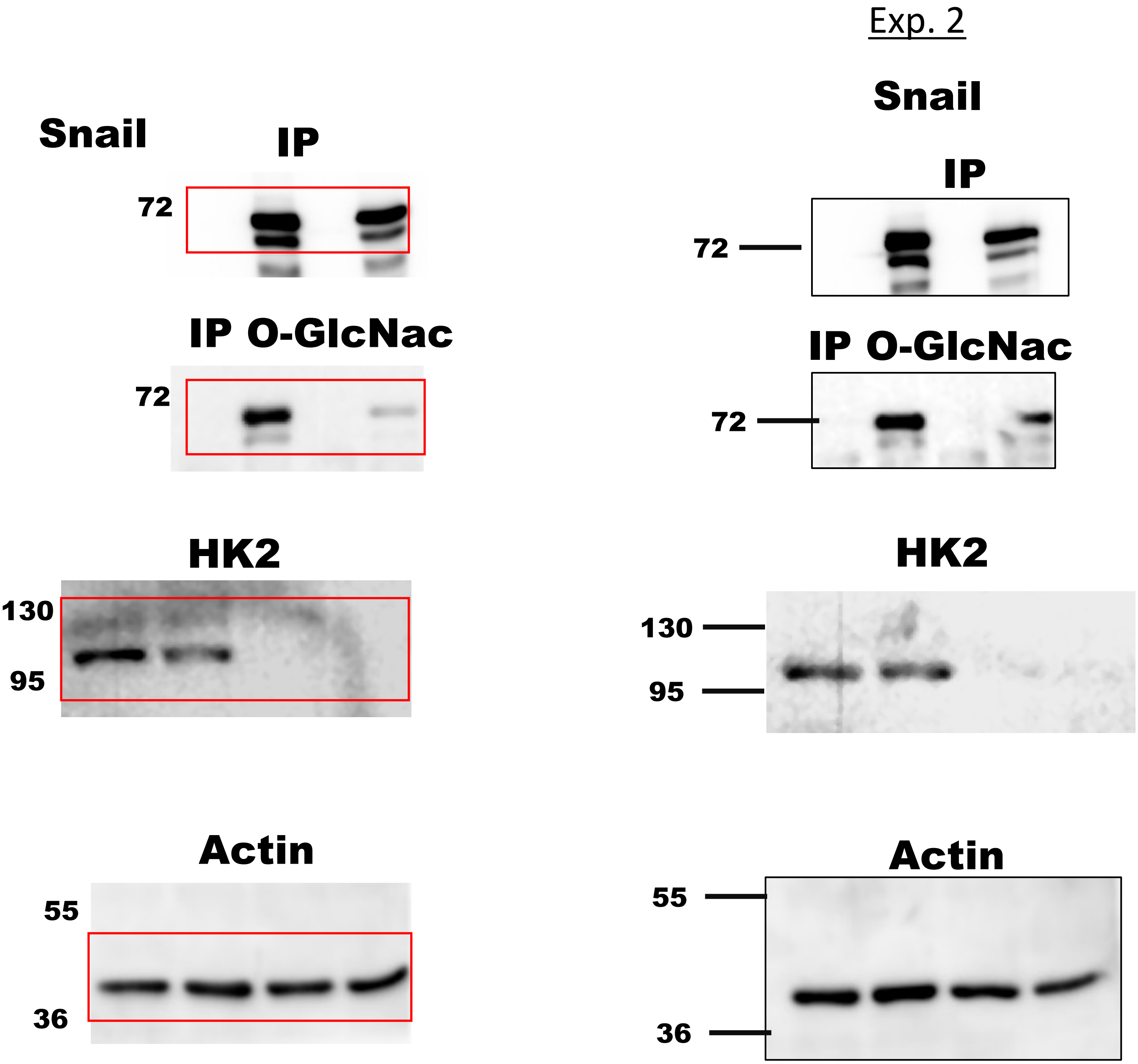
Exp. 2
Snail
IP
72
IP O-GlcNac
72
HK2
130
95
Actin
55
36
Snail
IP
72
IP O-GlcNac
72
HK2
130
95
Actin
55
36

Supplmetary Fig. 1

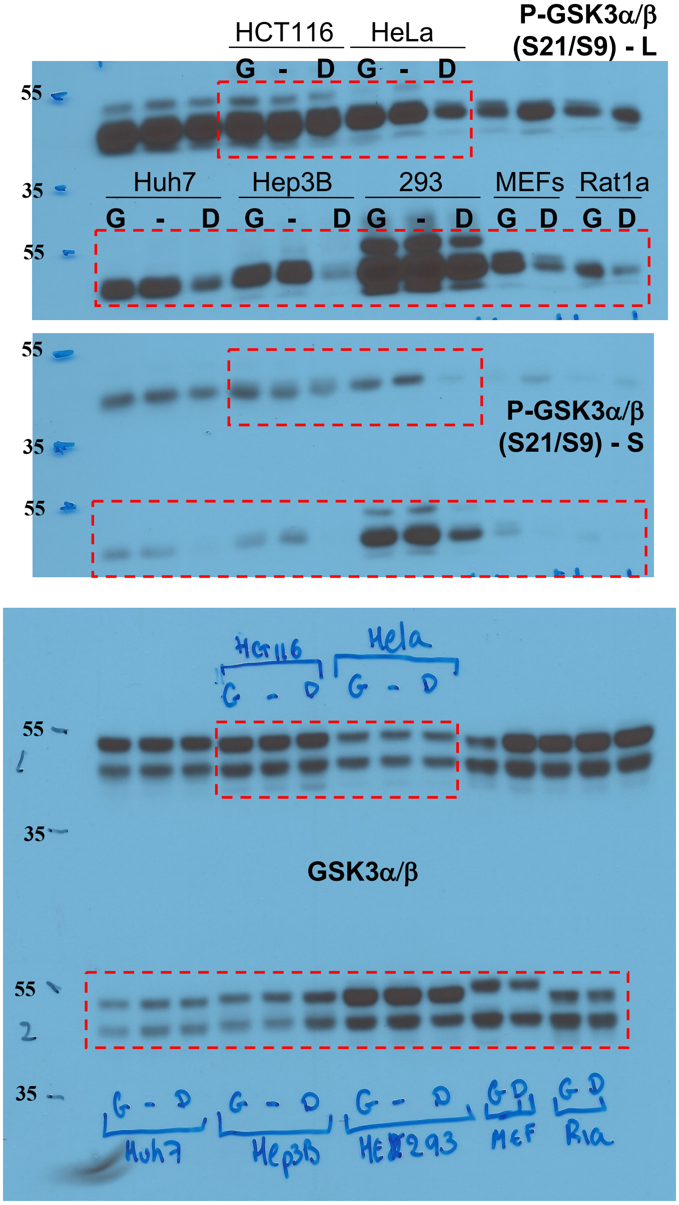

Additional Experiments

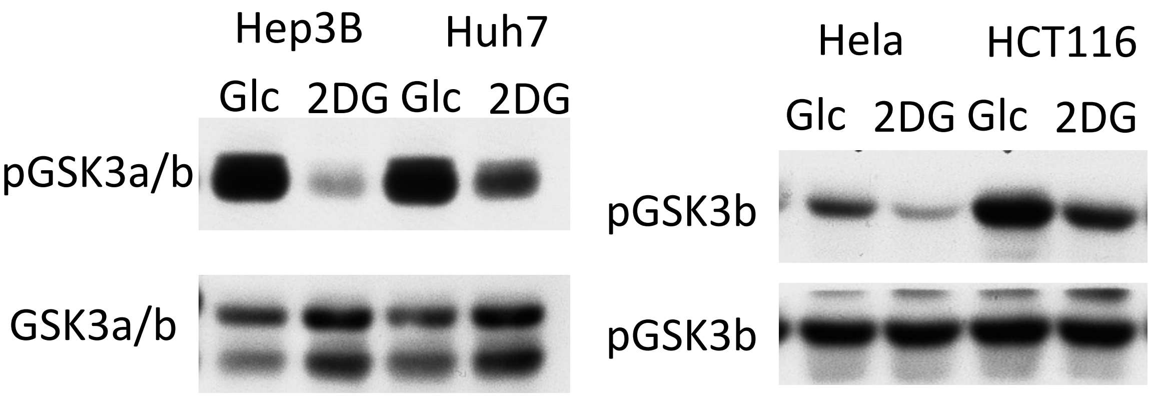

Supplementary Fig. 2

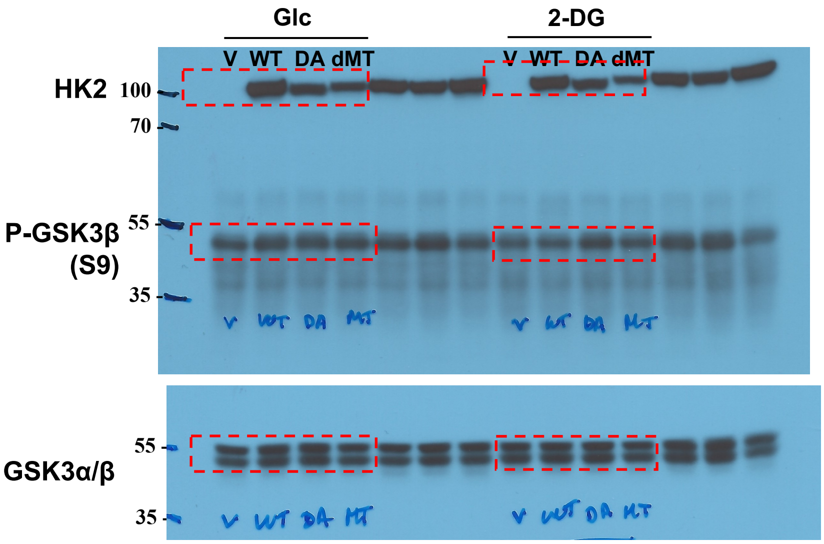

Suppelementary Fig. 3a

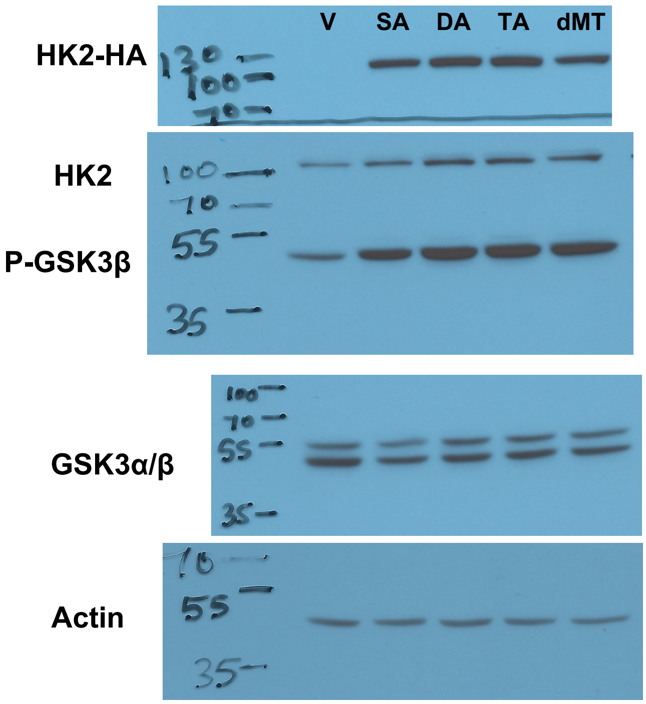

Extended Data Fig. 3b

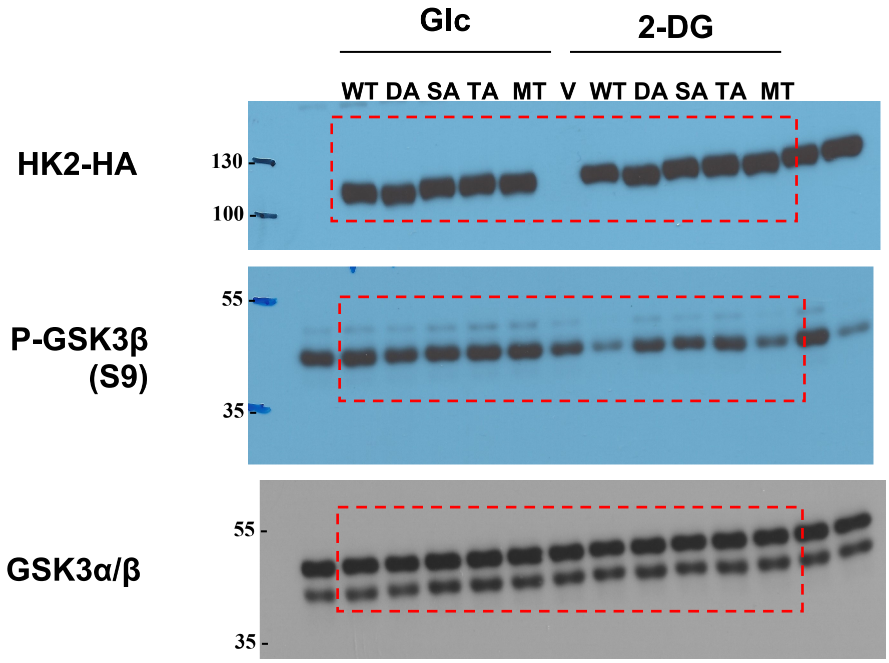

Supplementary Fig. 4a/b

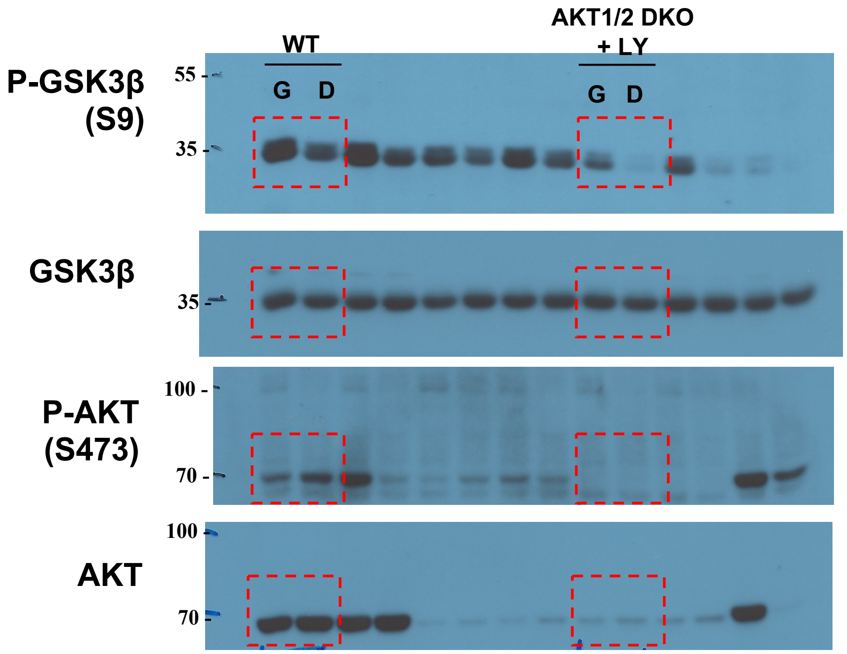

Extended Data Fig. 4c

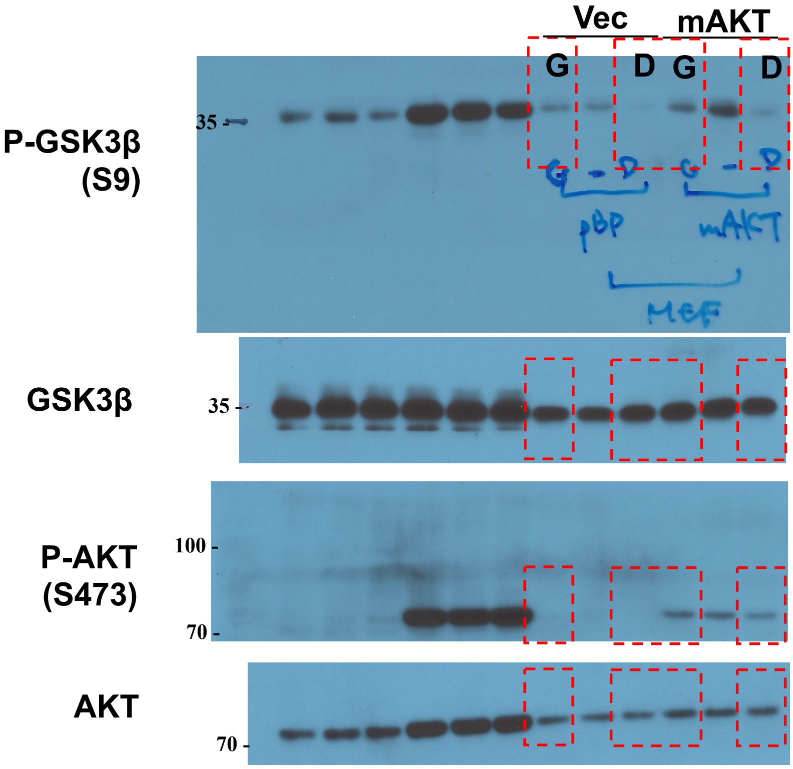

Supplementary Data Fig. 5a

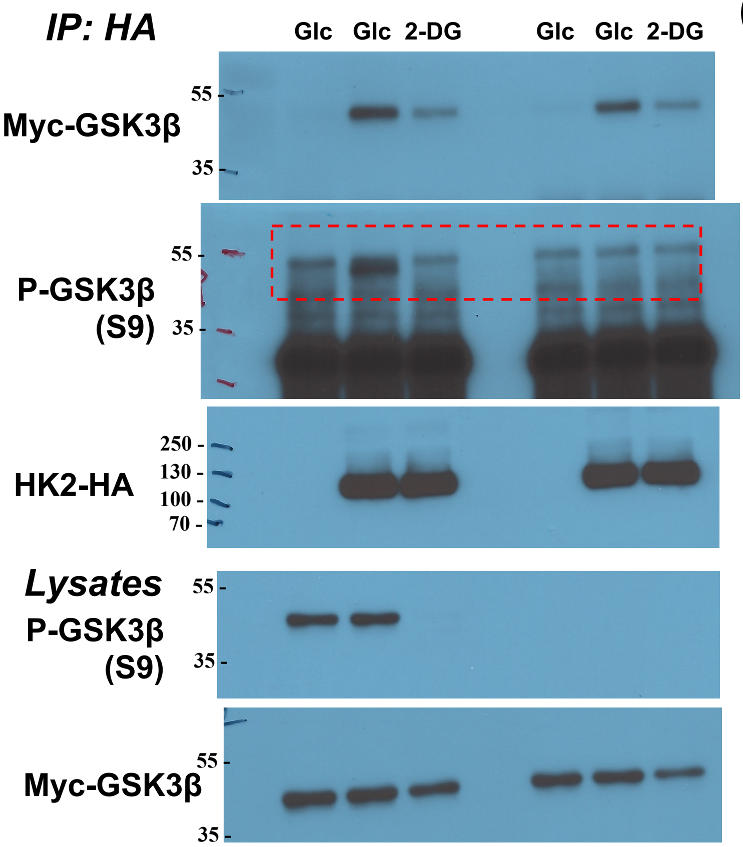

Experiment 2

(Co-IP HK2-HA and Myc-GSK3b (S9A)

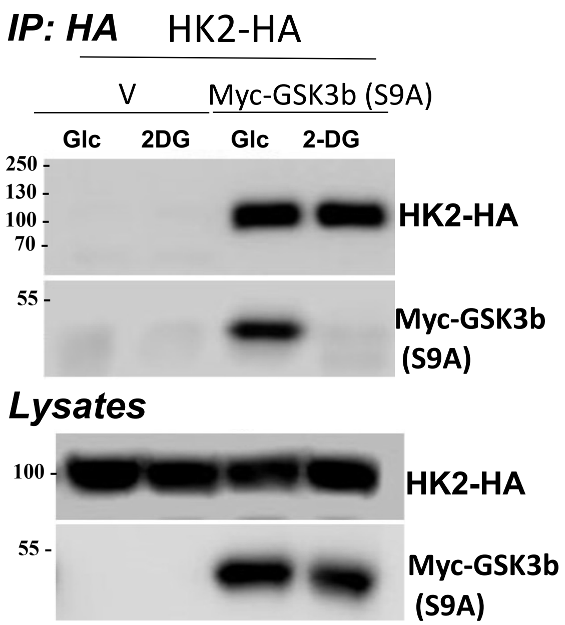

Supplementary Fig. 5b

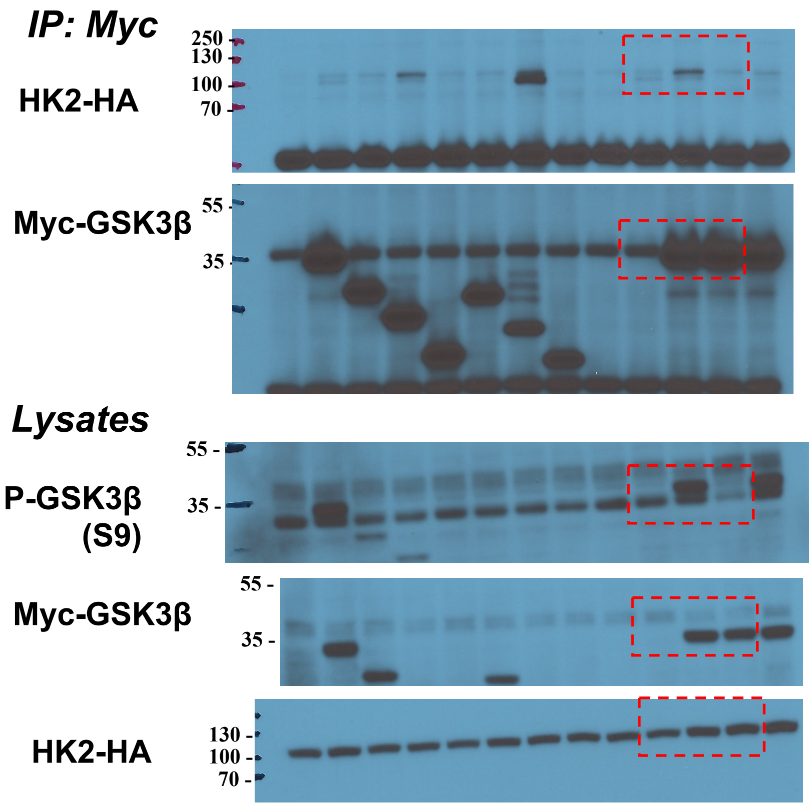

Supplementary Fig. 5c

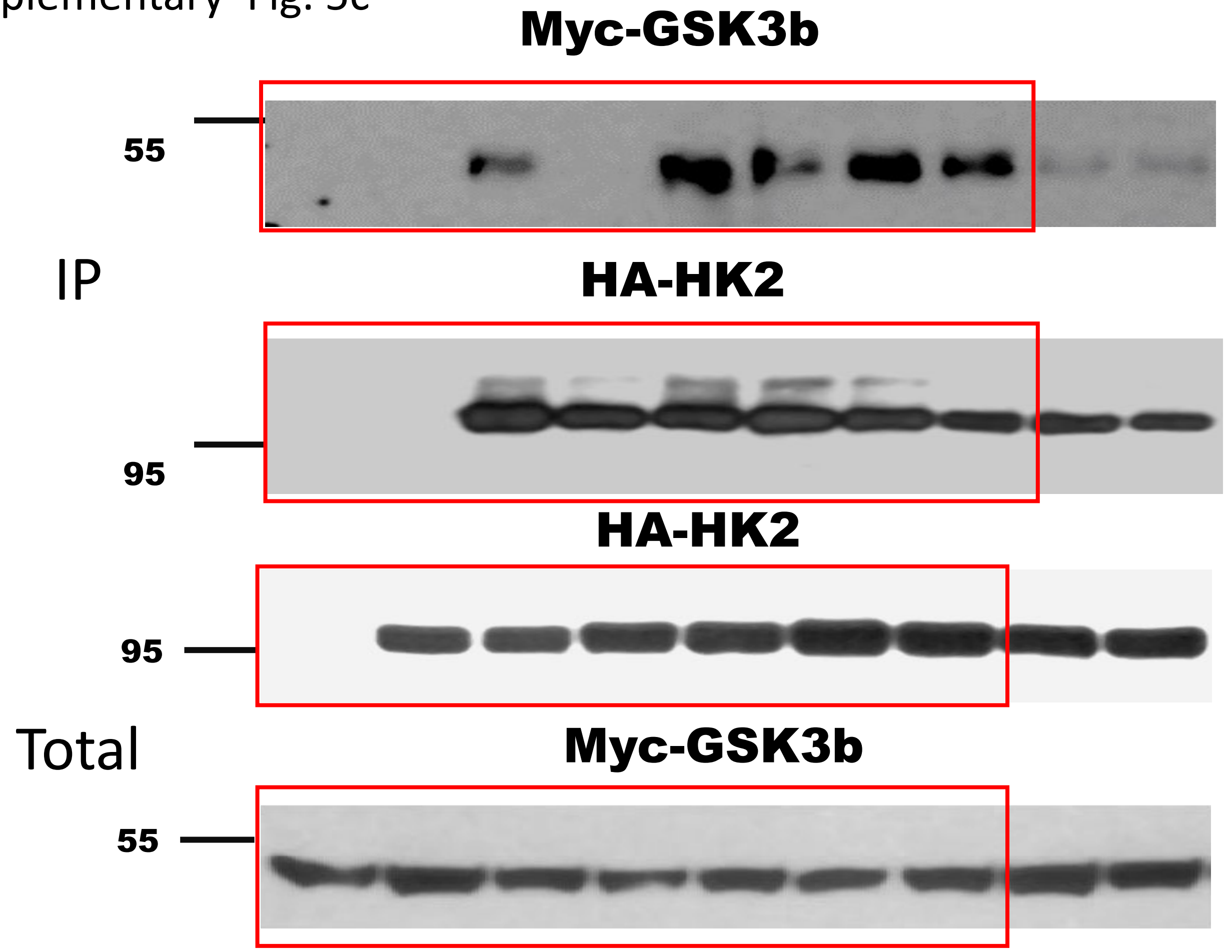

Supplementary Fig. 5c (Exp. 2)

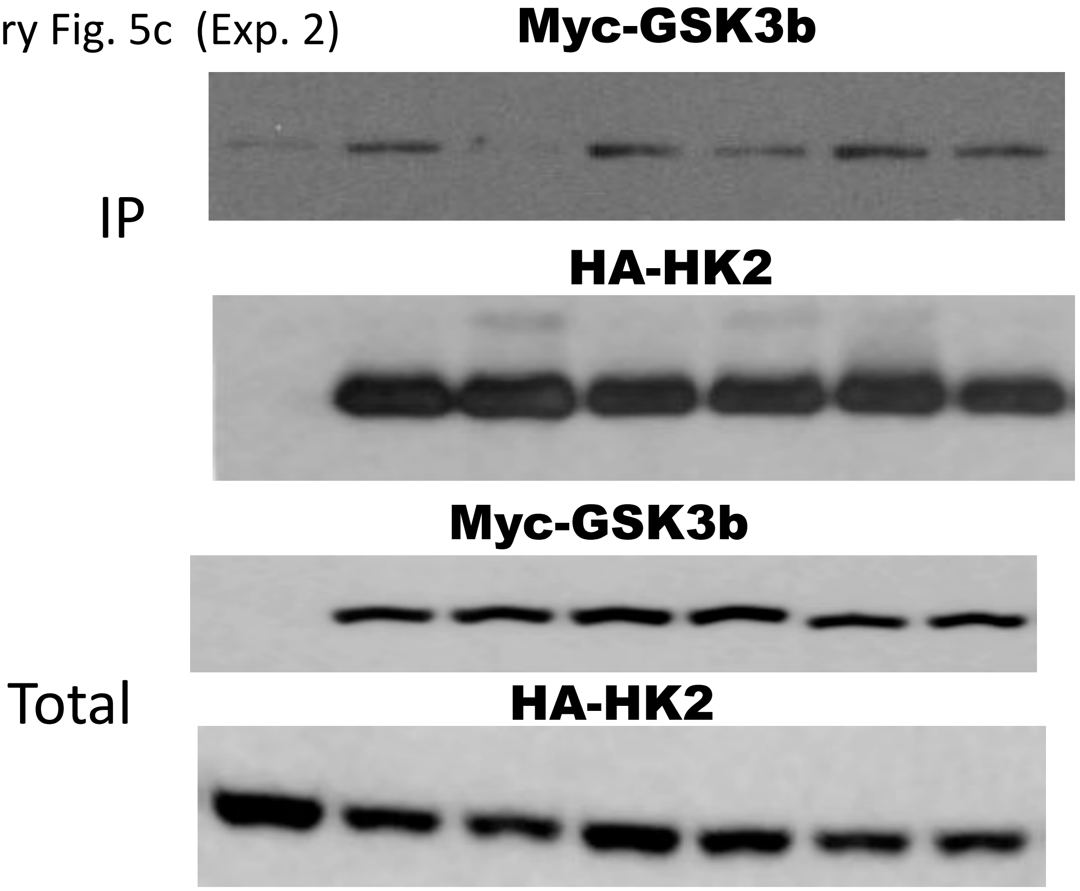

Supplementary Fig. 6

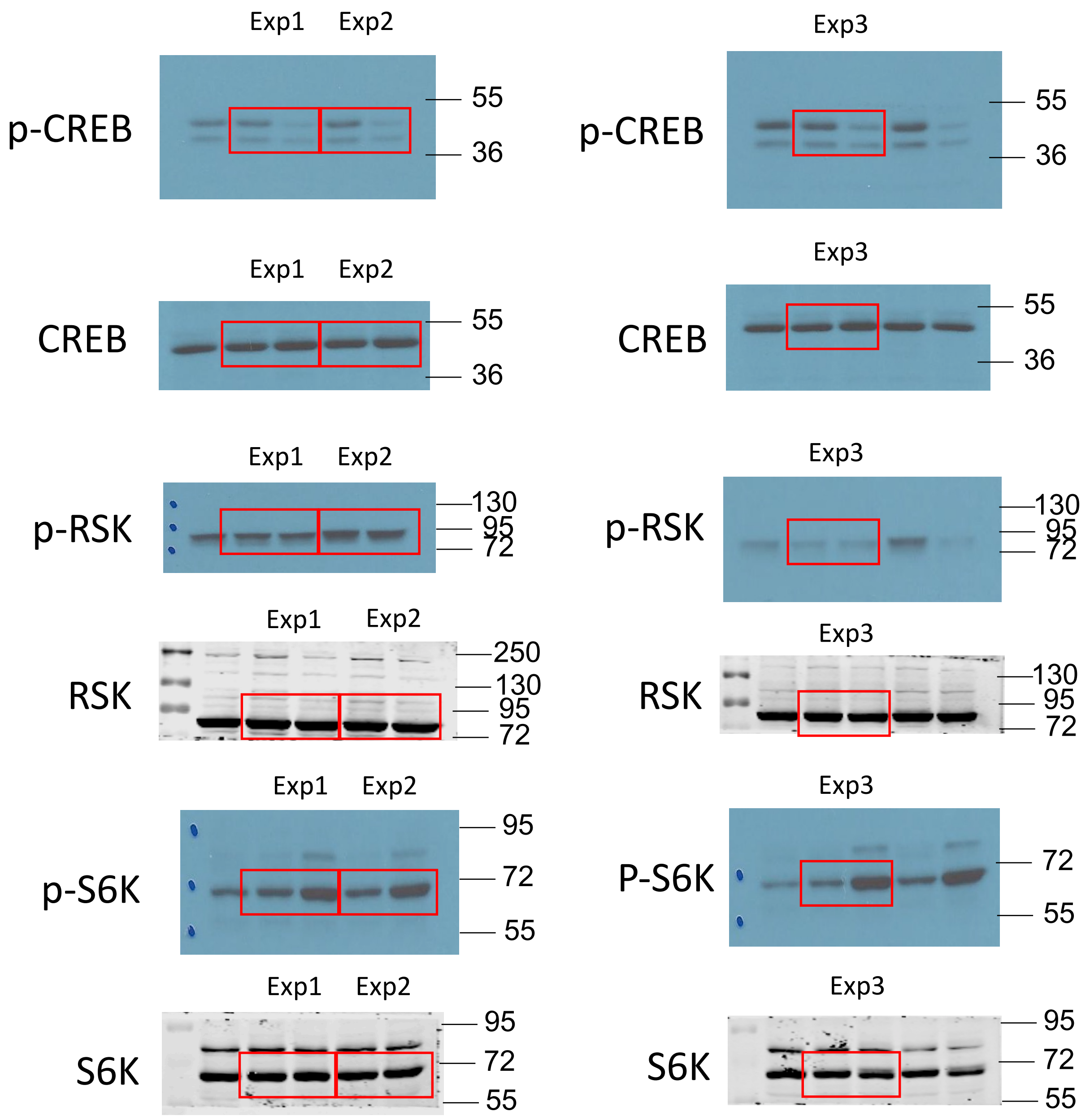

Supplementary Fig. 6 (continued)

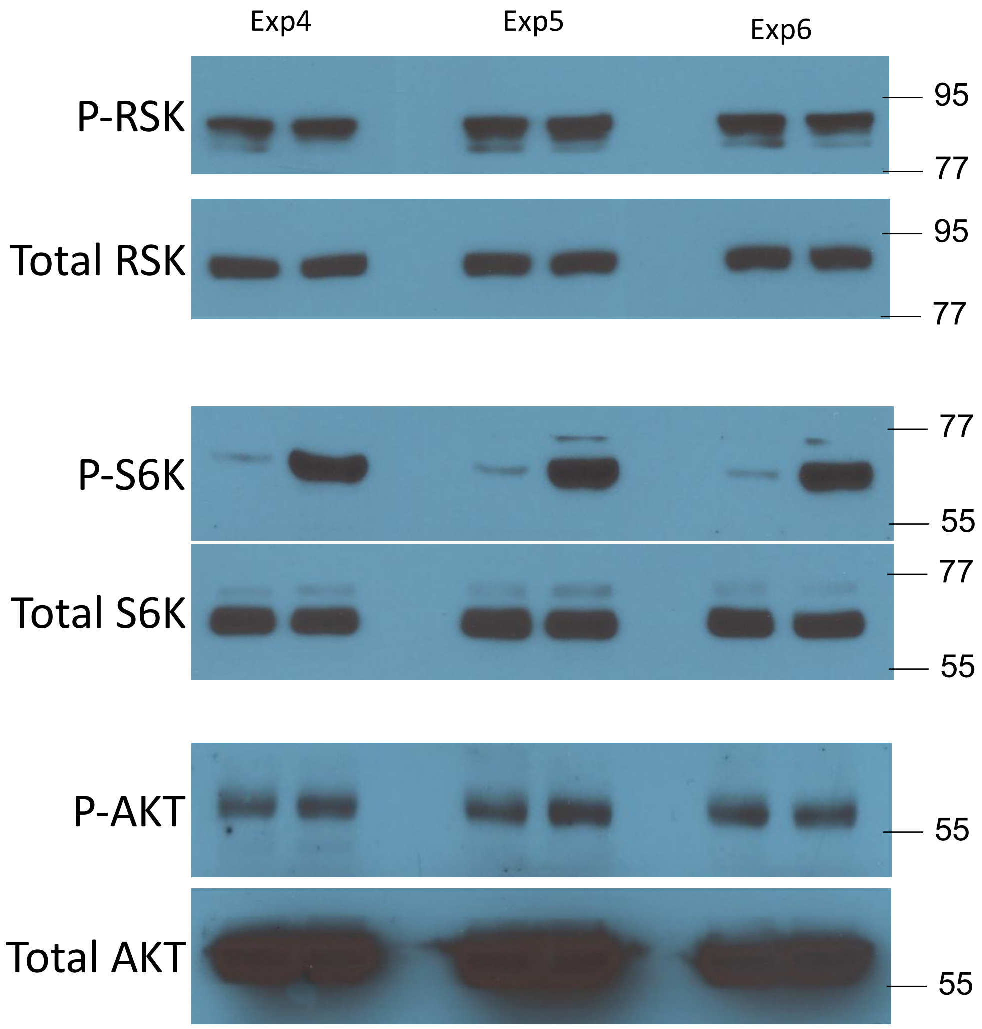

Supplementary Fig. 6 (continued)

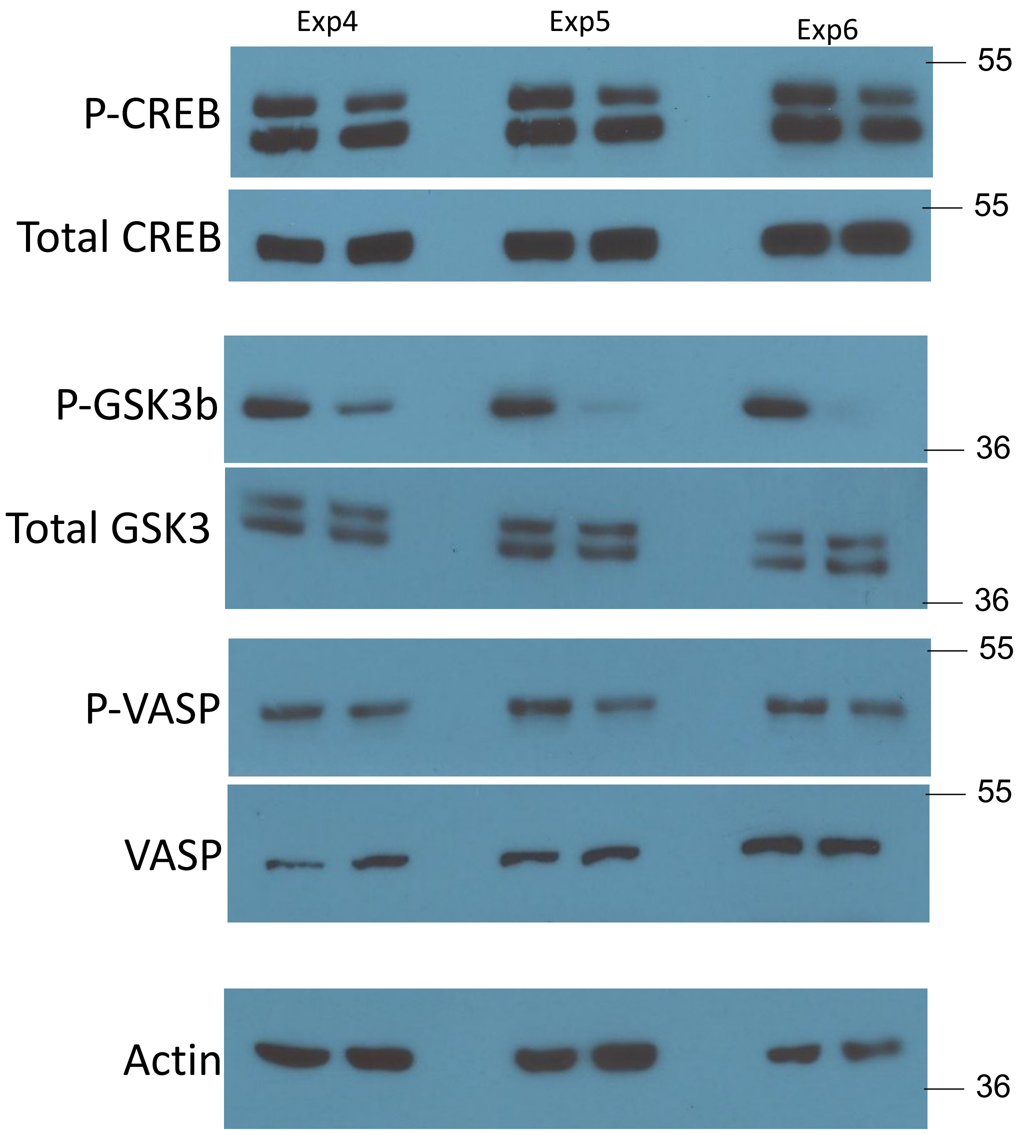

Supplementary Fig. 8a

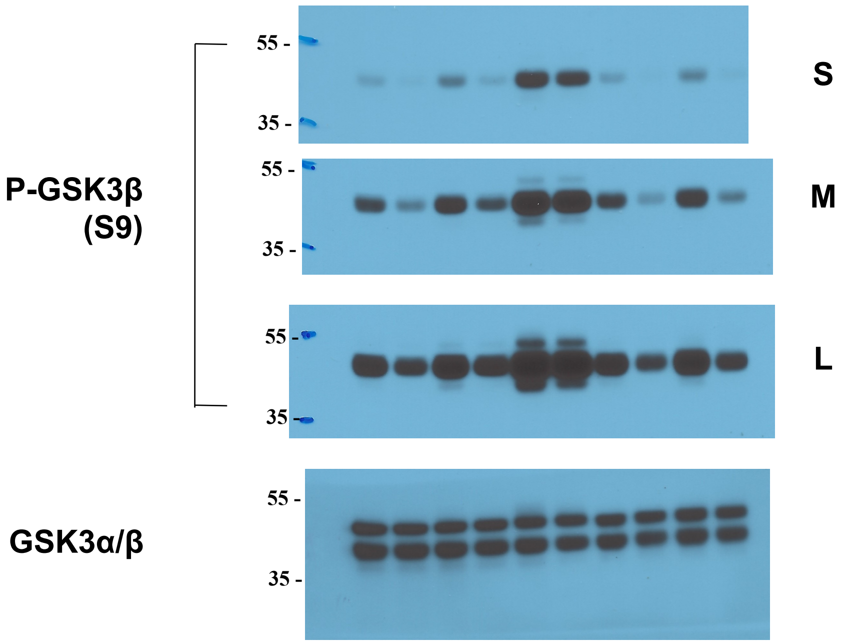

Supplementary Fig. 8b

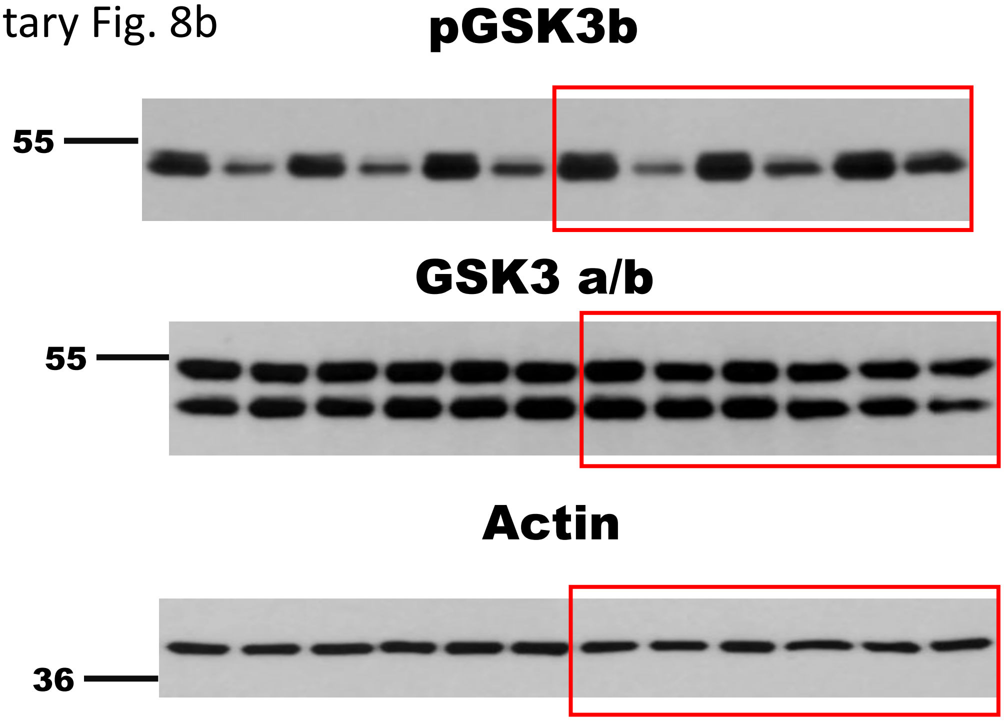

Additional Experiments for quantifications

Exp. 2

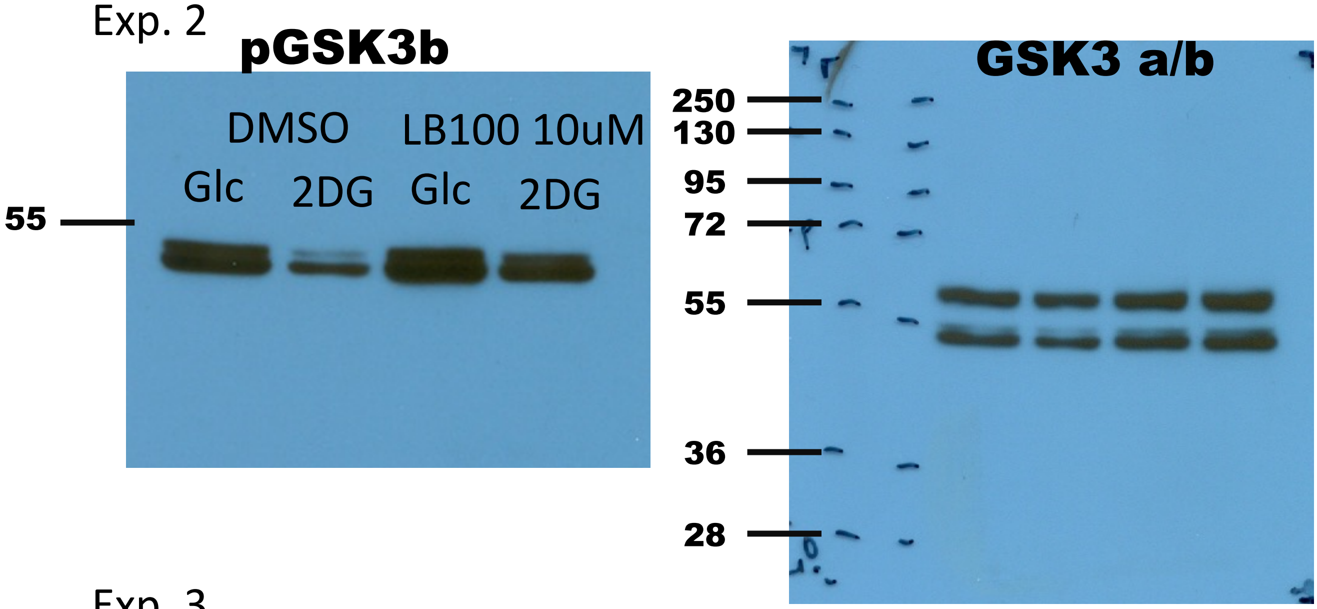

Exp. 3

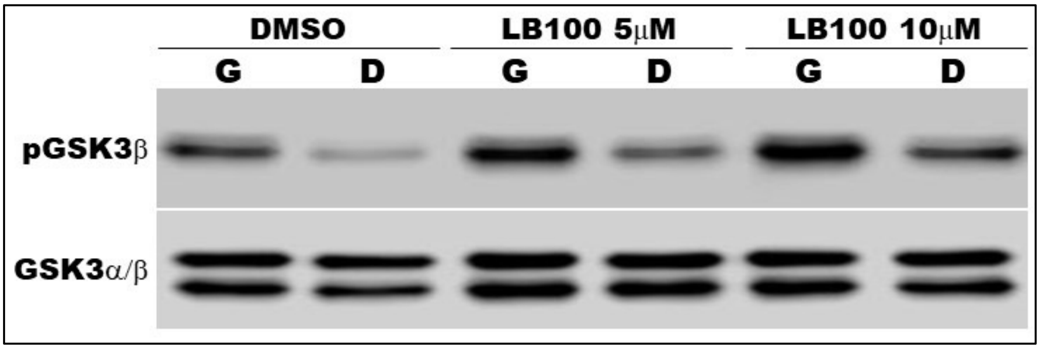

Supplementary Fig. 9

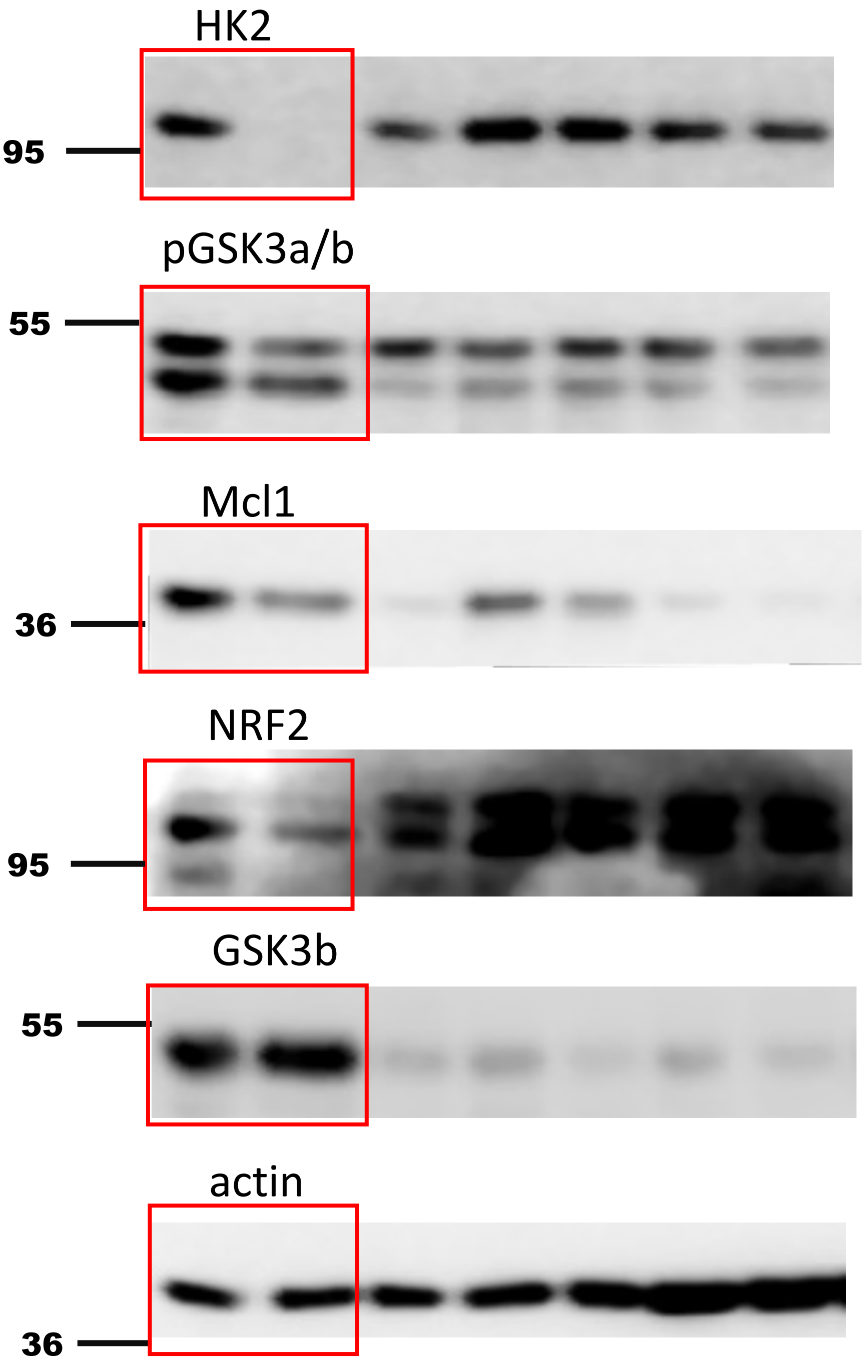

Supplementary Fig. 9 (Exp. 2)

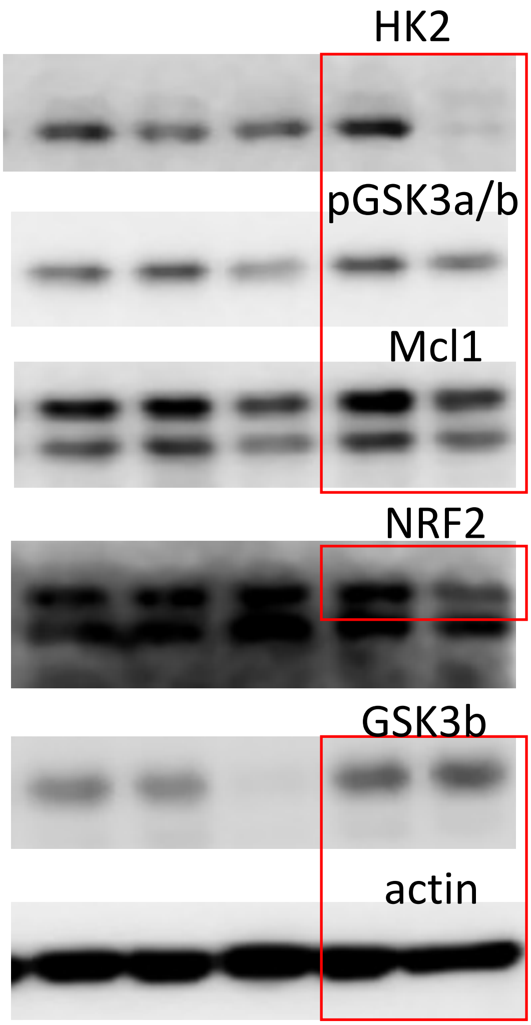

Supplementary Fig. 14a

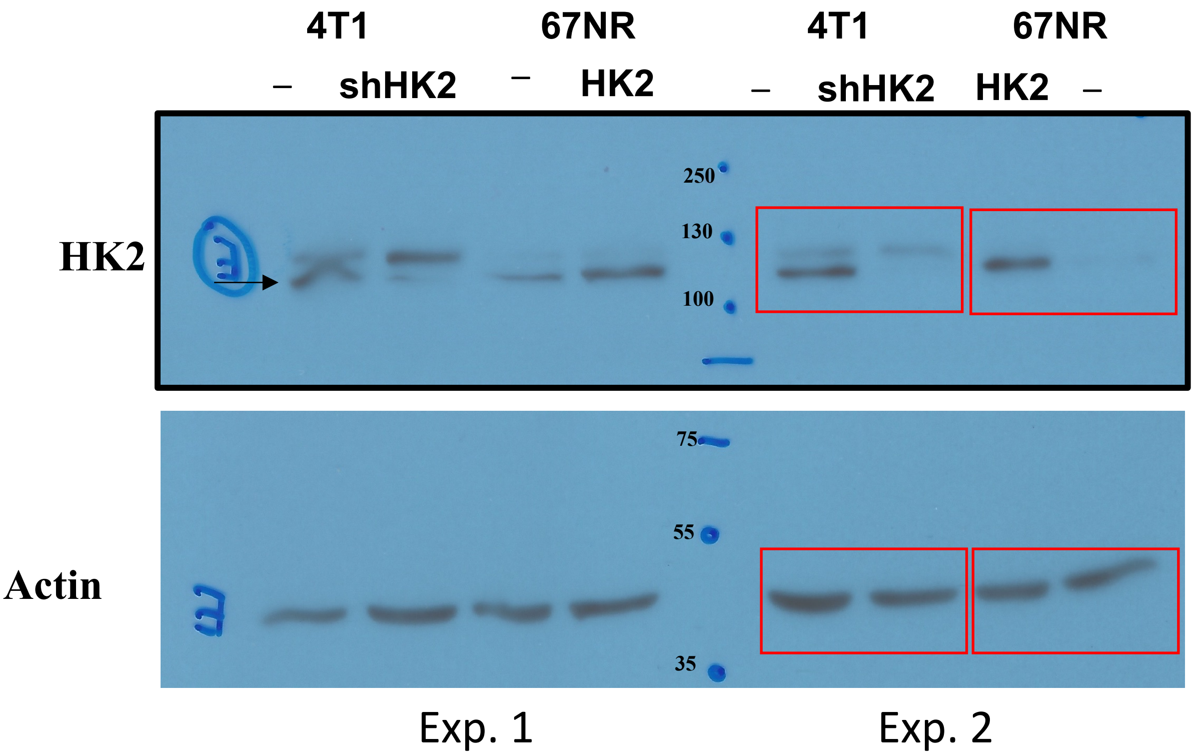

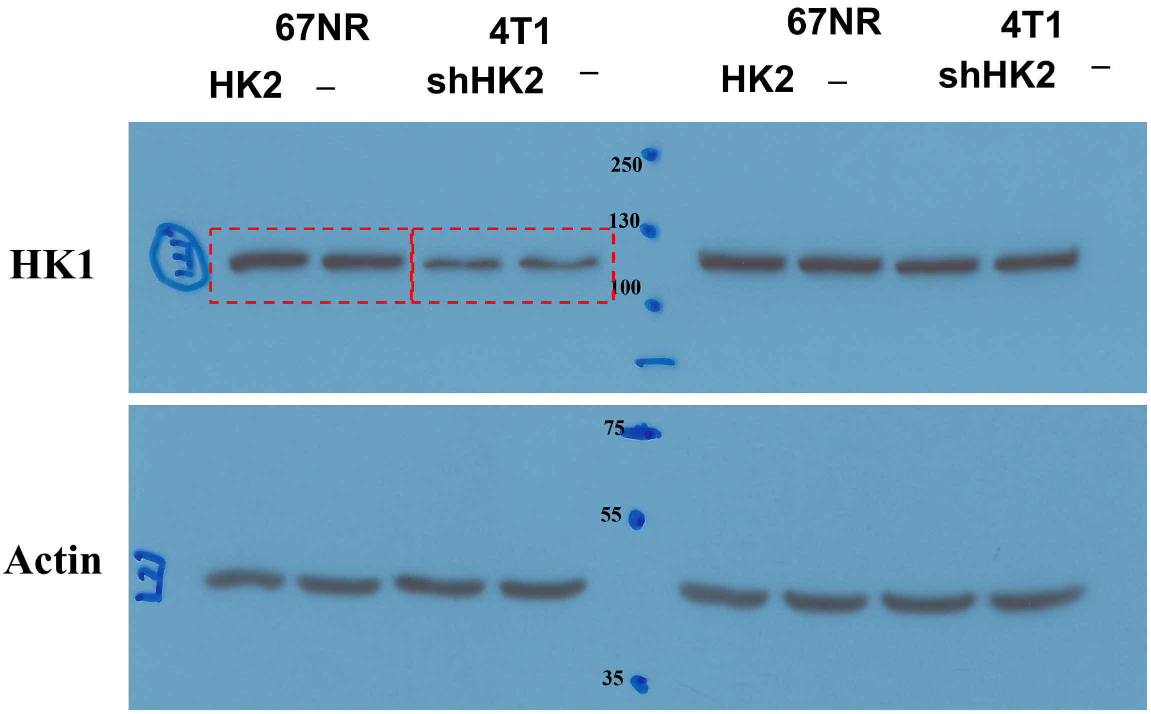

Supplementary Fig. 15a

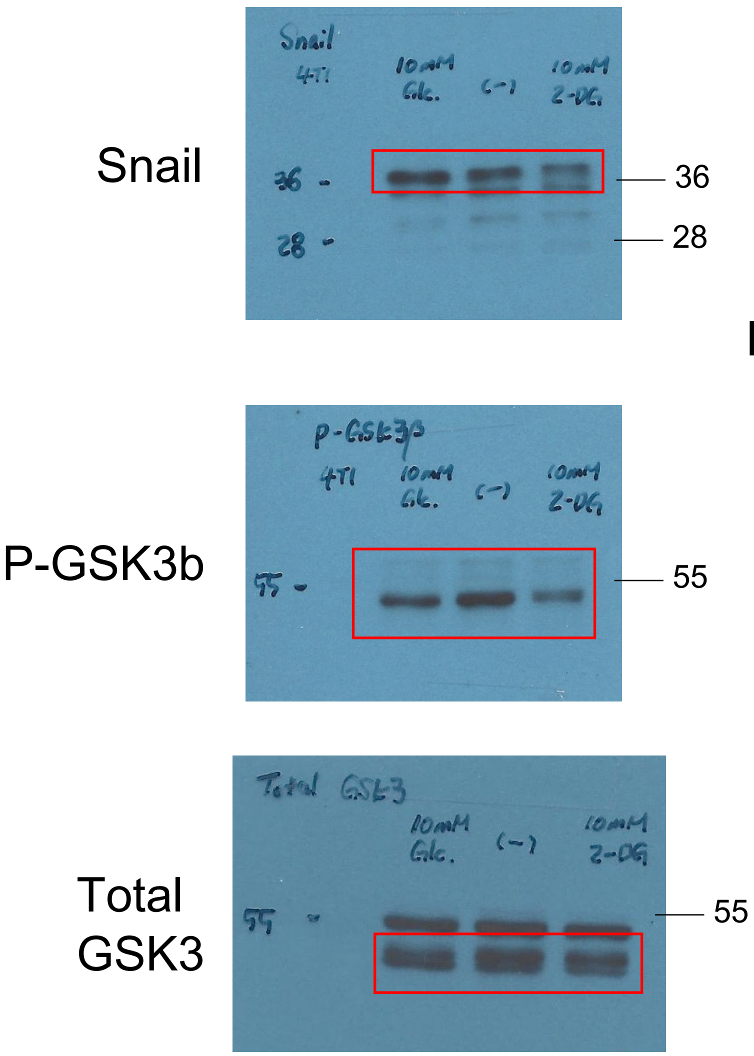

Supplementary Fig. 14b

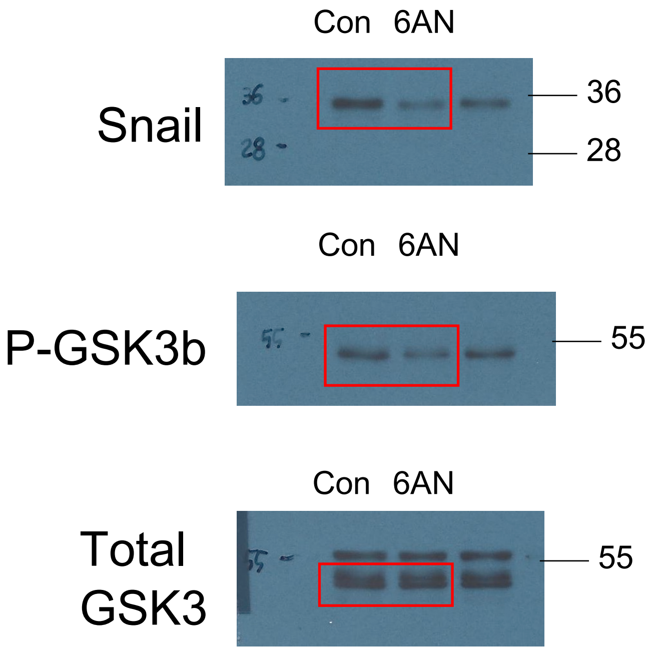

Additional experiments used for quantifications

① p-GSK3β

Supplementary Fig. 15a

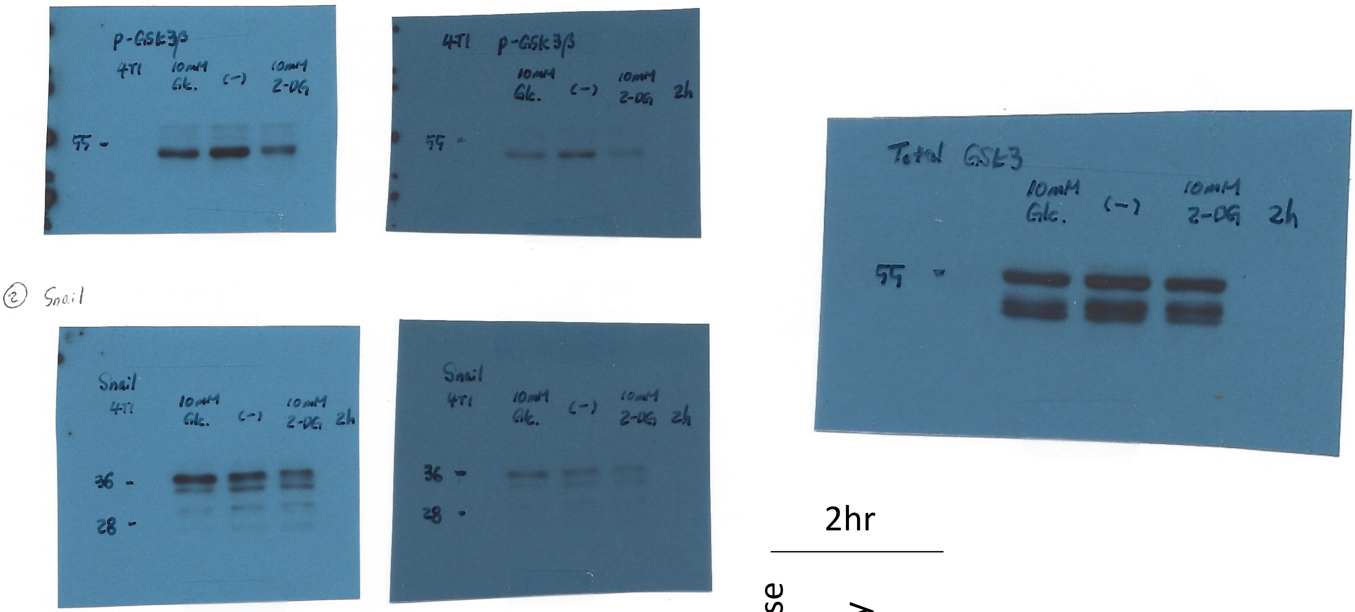

Extended data Fig. 14a

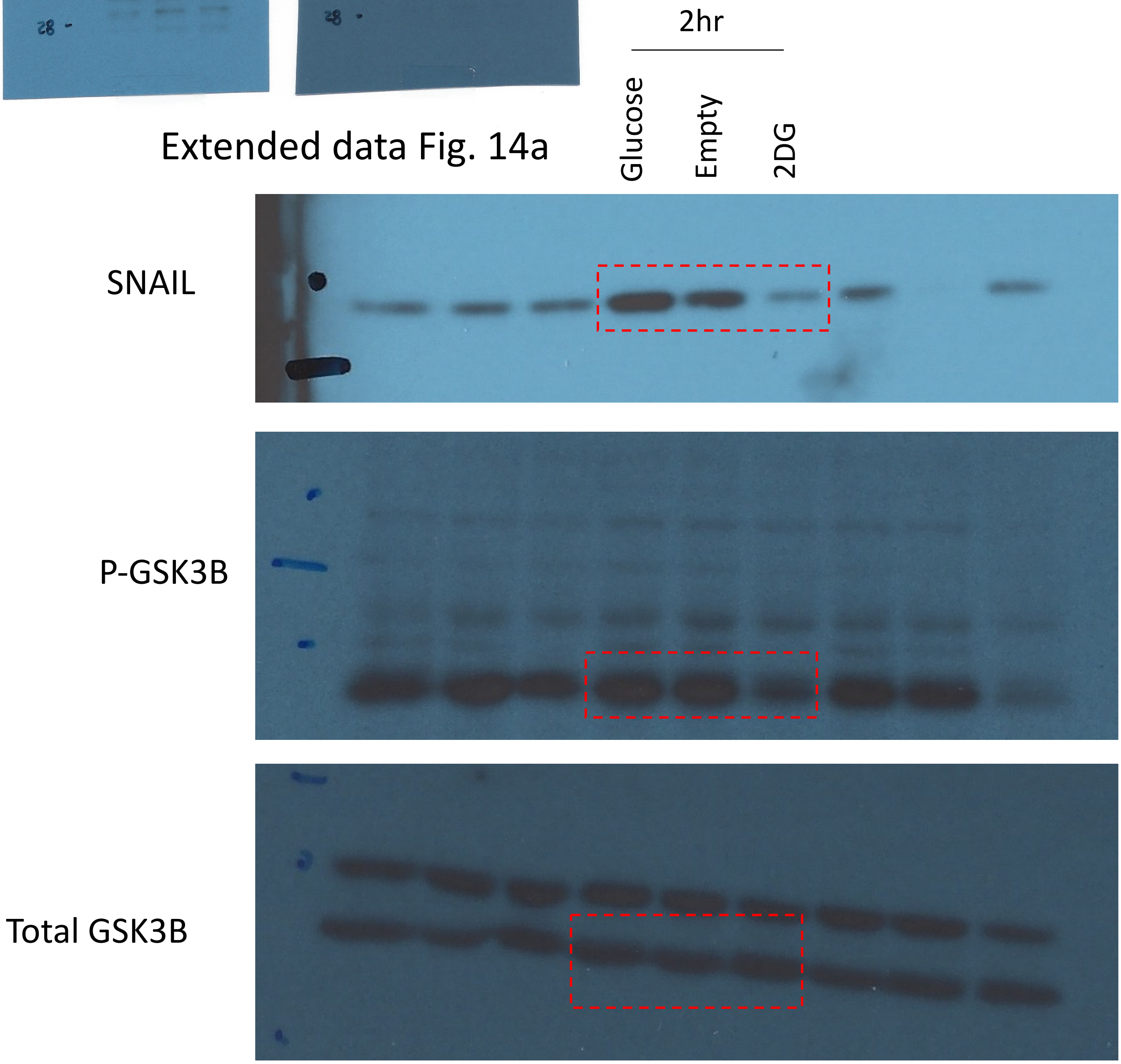

<u>Additional experiments used for quantifications</u>

Supplemntary Fig. 15a

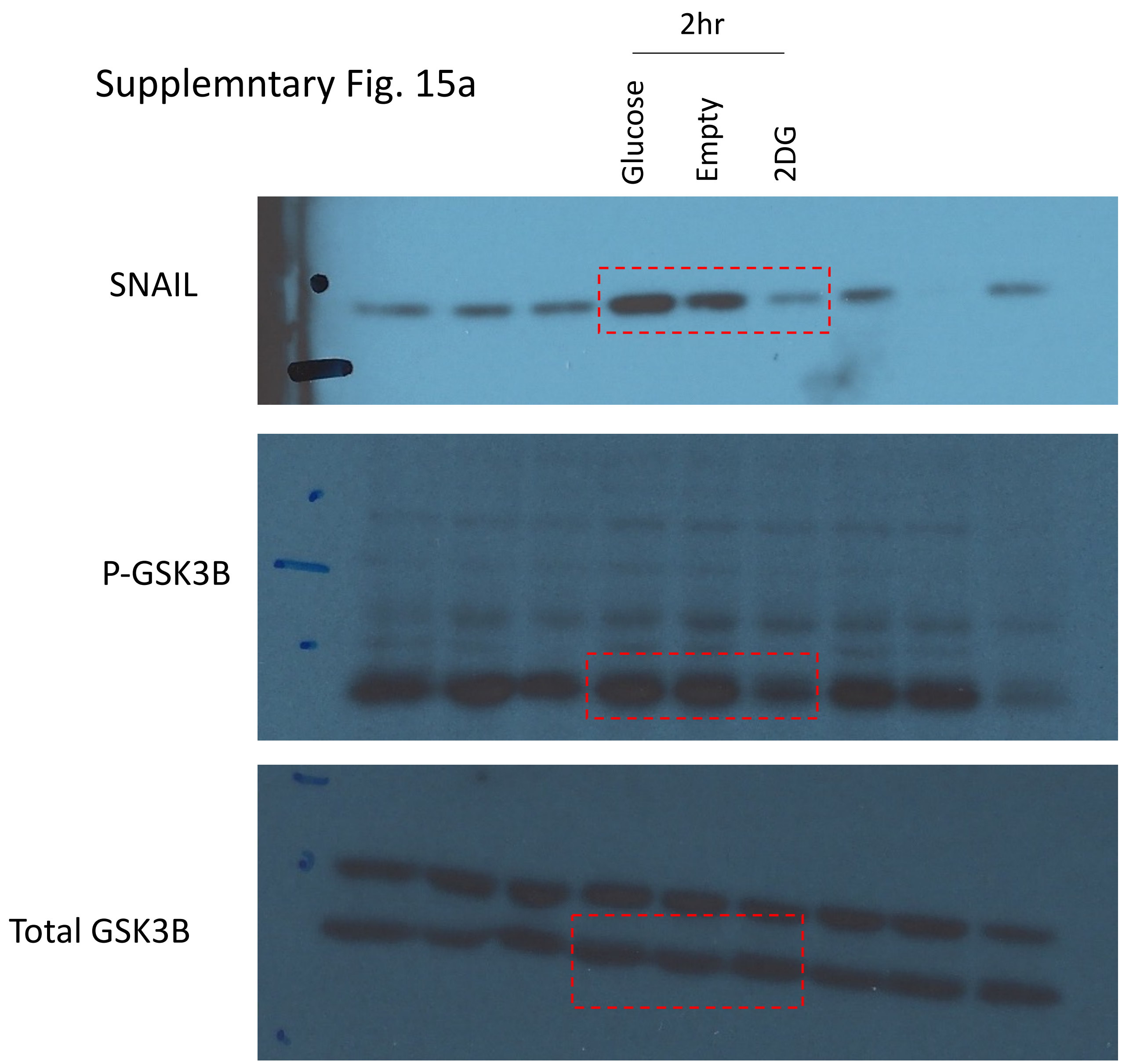

Additional experiments used for quantifications

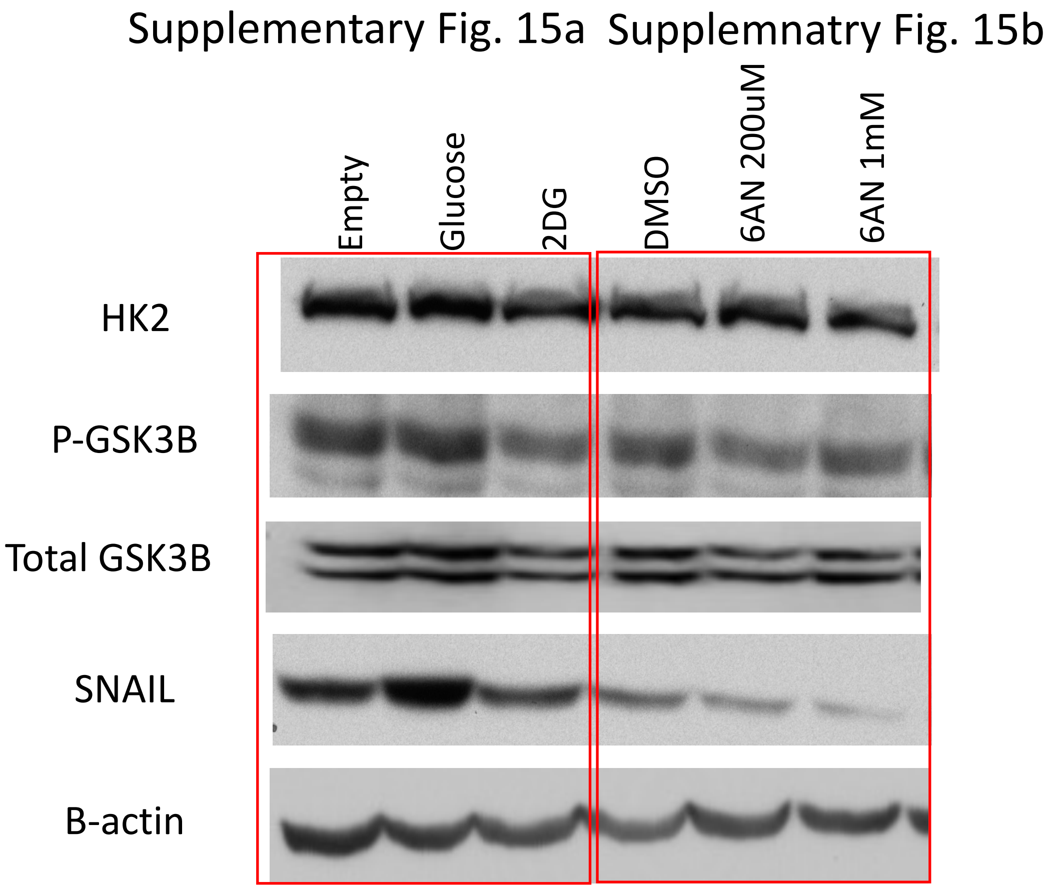

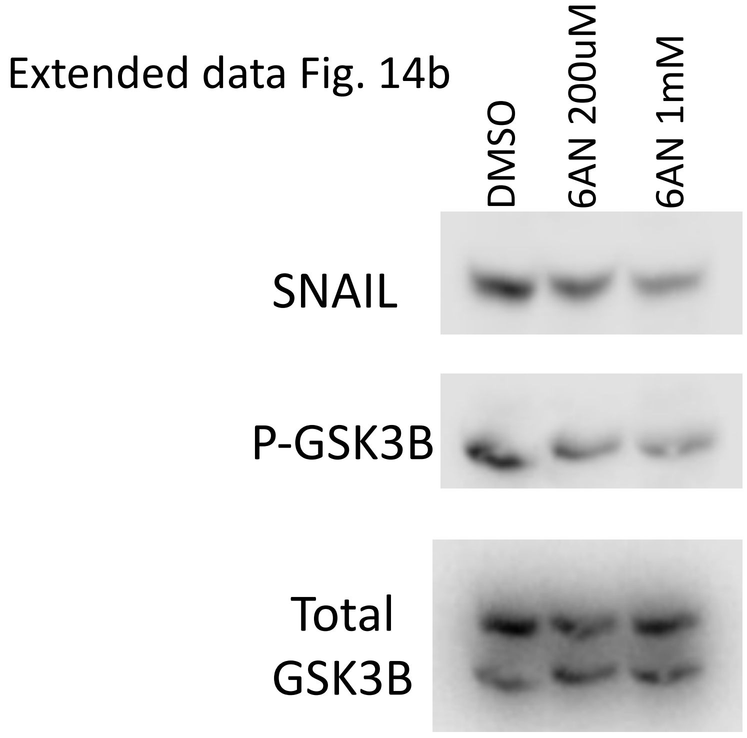

<u>Additional experiments used for quantifications</u>

Supplemenatry Fig. 15b

P-GSK3b

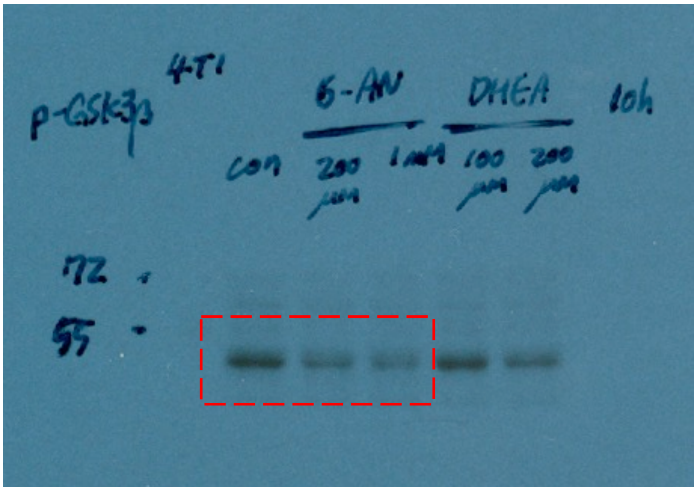

Total GSK3

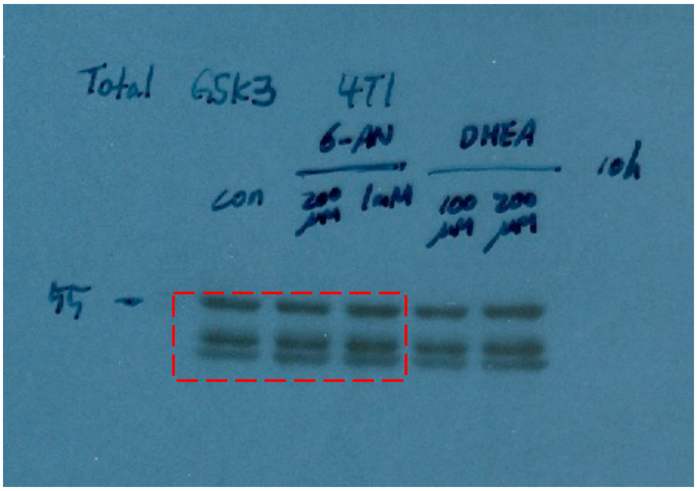

Snail

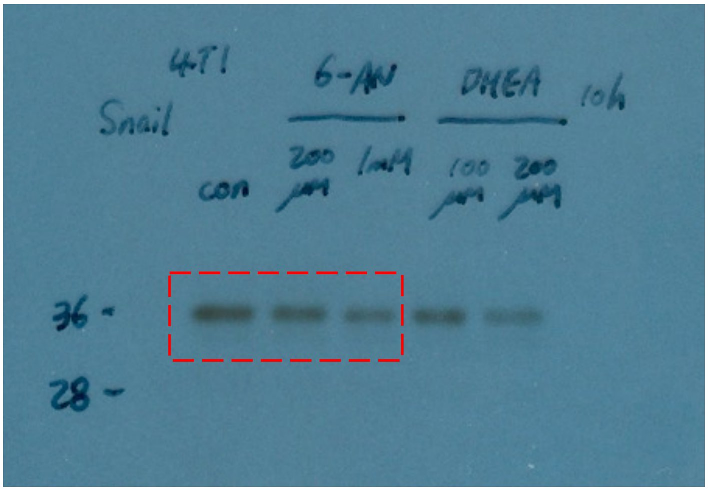

Supplementary Fig. 15c

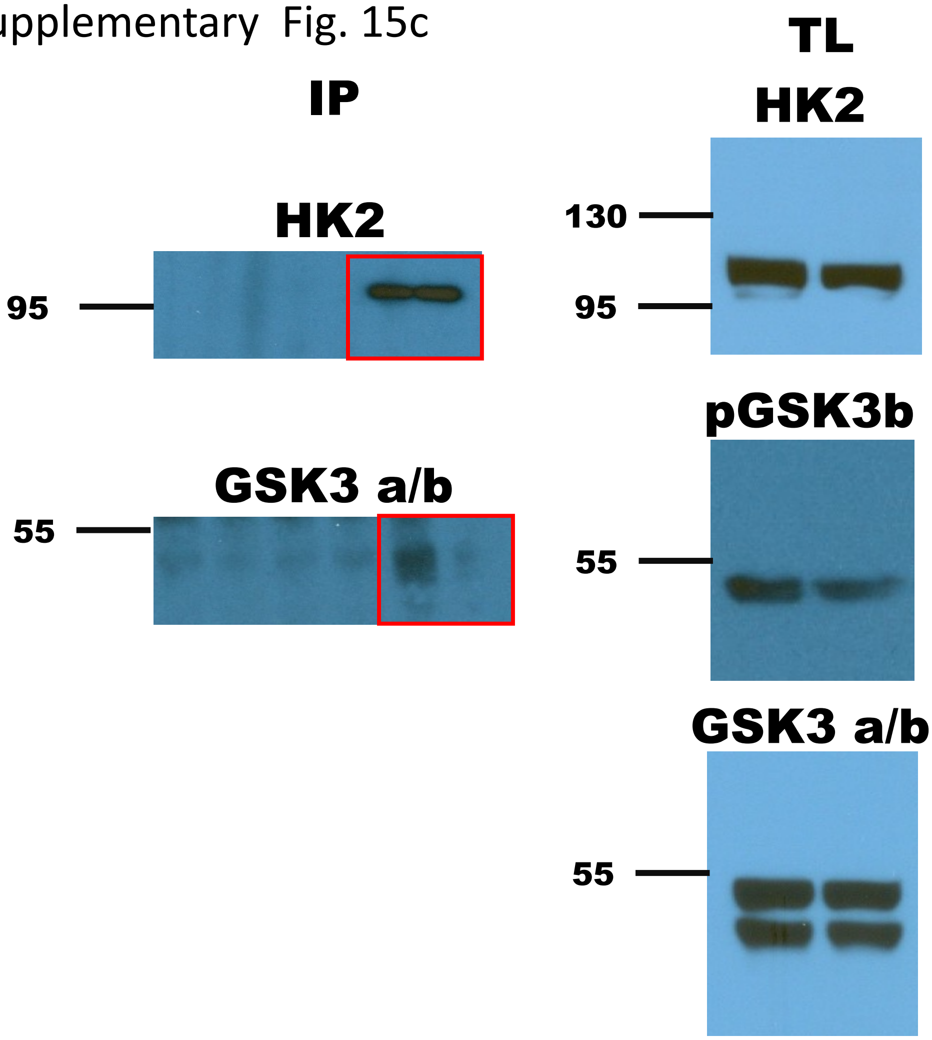

Supplement: Supplementary file 1 — Supplementary Information [file 41467_2022_28440_MOESM1_ESM.pdf]
