# Supplementary material for: A non-catalytic scaffolding activity of hexokinase 2 contributes to EMT and metastasis
Source: Nat Commun. 2022 Feb 16;13:899. doi: 10.1038/s41467-022-28440-3 (PMC8850586; doi:10.1038/s41467-022-28440-3)
Supplement: Supplementary file 2 — Description of Additional Supplementary Files [file 41467_2022_28440_MOESM2_ESM.pdf]

Description of Additional Supplementary Files

File Name: Supplementary Data 1
Description: Average Gene Expression In The Individual Clusters

File Name: Supplementary Data 2
Description: Genes expressed at the highest level in each cluster
