# Supplementary material for: A non-catalytic scaffolding activity of hexokinase 2 contributes to EMT and metastasis
Source: Nat Commun. 2022 Feb 16;13:899. doi: 10.1038/s41467-022-28440-3 (PMC8850586; doi:10.1038/s41467-022-28440-3)
Supplement: Supplementary file 5 — Reporting Summary [file 41467_2022_28440_MOESM5_ESM.pdf]

nature portfolio

Corresponding author(s): Nissim Hay

Last updated by author(s): Jan 2, 2022

Reporting Summary

Nature Portfolio wishes to improve the reproducibility of the work that we publish. This form provides structure for consistency and transparency in reporting. For further information on Nature Portfolio policies, see our Editorial Policies and the Editorial Policy Checklist.

Statistics

For all statistical analyses, confirm that the following items are present in the figure legend, table legend, main text, or Methods section.

n/a	Confirmed	
☐	☒	The exact sample size (n) for each experimental group/condition, given as a discrete number and unit of measurement
☐	☒	A statement on whether measurements were taken from distinct samples or whether the same sample was measured repeatedly
☐	☒	The statistical test(s) used AND whether they are one- or two-sided<br>*Only common tests should be described solely by name; describe more complex techniques in the Methods section.*
☒	☐	A description of all covariates tested
☒	☐	A description of any assumptions or corrections, such as tests of normality and adjustment for multiple comparisons
☐	☒	A full description of the statistical parameters including central tendency (e.g. means) or other basic estimates (e.g. regression coefficient) AND variation (e.g. standard deviation) or associated estimates of uncertainty (e.g. confidence intervals)
☐	☒	For null hypothesis testing, the test statistic (e.g. F, t, r) with confidence intervals, effect sizes, degrees of freedom and P value noted<br>*Give P values as exact values whenever suitable.*
☒	☐	For Bayesian analysis, information on the choice of priors and Markov chain Monte Carlo settings
☒	☐	For hierarchical and complex designs, identification of the appropriate level for tests and full reporting of outcomes
☒	☐	Estimates of effect sizes (e.g. Cohen's d, Pearson's r), indicating how they were calculated

Our web collection on statistics for biologists contains articles on many of the points above.

Software and code

Policy information about availability of computer code

Data collection	PRISM 6.0 , Excel.Statistics for each experiment were justified as appropriate, because the data does meet the assumptions of the test. Estimates of variation are based on individual tests and were within limits, and the variance between groups compared were similar.
Data analysis	Seurat package (version v2.2.1) using R (version 3.3.2)

For manuscripts utilizing custom algorithms or software that are central to the research but not yet described in published literature, software must be made available to editors and reviewers. We strongly encourage code deposition in a community repository (e.g. GitHub). See the Nature Portfolio guidelines for submitting code & software for further information.

Data

Policy information about availability of data

All manuscripts must include a data availability statement. This statement should provide the following information, where applicable:

- Accession codes, unique identifiers, or web links for publicly available datasets
- A description of any restrictions on data availability
- For clinical datasets or third party data, please ensure that the statement adheres to our policy

accession number GSE148445.

Field-specific reporting

Please select the one below that is the best fit for your research. If you are not sure, read the appropriate sections before making your selection.

☒ Life sciences ☐ Behavioural & social sciences ☐ Ecological, evolutionary & environmental sciences

For a reference copy of the document with all sections, see nature.com/documents/nr-reporting-summary-flat.pdf

Life sciences study design

All studies must disclose on these points even when the disclosure is negative.

Sample size	For animal studies, we performed power analyses using a web-based tool at www.biomath.info. Within group, variation is expected to be about 30% at the end-point and we are expecting a difference of at least 50% between 2 groups. Based on those 8 tumors/group will have sufficient power with a confidence level of 0.95.
Data exclusions	There is one example in the paper in which one mouse was excluded based on analysis by Prism (Fig. 9e). This figure shows that transient inducible expression of SNAIL rescues the ability of 4T1shHK2 cells to metastasize. All five mice showed the same trend. However, one mouse had more than 10-fold higher basal metastases when compared to the rest of the mice. The GraphPad Prism Software Version 8.0 recognized this mouse as an outlier. One possible explanation for the higher basal level of metastases in this mouse is a the "leakiness" of the inducible system.
Replication	WBs quantifications were done with at least two independent experiments.
Randomization	The only experiments that required randomization were the in-vivo studies, where by groups were assigned to provide the most consistency in tumor size across groups at the start of the experiment.
Blinding	Quantifications of lung metastases was done blindly. No other quantifications required blinding.

Reporting for specific materials, systems and methods

We require information from authors about some types of materials, experimental systems and methods used in many studies. Here, indicate whether each material, system or method listed is relevant to your study. If you are not sure if a list item applies to your research, read the appropriate section before selecting a response.

Materials & experimental systems

n/a	Involved in the study	
☐	☒	Antibodies
☐	☒	Eukaryotic cell lines
☒	☐	Palaeontology and archaeology
☐	☒	Animals and other organisms
☒	☐	Human research participants
☒	☐	Clinical data
☒	☐	Dual use research of concern

Methods

n/a	Involved in the study	
☒	☐	ChIP-seq
☒	☐	Flow cytometry
☒	☐	MRI-based neuroimaging

Antibodies

Antibodies used

All antibodies used herein are commercially available and have been validated by the companies that provide them.

Rabbit monoclonal Hexokinase II (C64G5) Cell signaling 2867
HXK II (C-14)
(Used for immunoprecipitation) Santa Cruz sc-6521
Mouse monoclonal E-cadherin (4A2) Cell signaling 14472
Rabbit monoclonal Vimentin (D12H3) Cell signaling 5741
Rabbit monoclonal Snail (C15D3) Cell signaling 3879
Mouse monoclonal B-actin (AC-15) Sigma-Aldrich A5441
Rabbit polyclonal Phopsho-GSK3b (Ser9) Cell signaling 9336
Mouse monoclonal Total GSK3b (4G-1E) Millipore 05-412
p-GSK3a/b antibody Cell signaling 9331
GSK3a/b Biosource 44-610
AMPK antibody Cell signaling 2532
p-ACC antibody Cell signaling 3661
Mcl-1 Santa Cruze Sc-819

HA-Tag (262) monoclonal antibody Cell signaling 2362
Myc-Tag (9B11) mouse monoclonal antibody Cell signaling 2276
Myc Tag Monoclonal Antibody, HRP Invitrogen R951-25
PRKAR1a (D5405) Rabbit mAb Cell signaling 5675
Anti-Myc Tag Monoclonal Antibody, HRP Invitrogen R951-25
Anti-cPKA antibody Cell Signaling 4782
PP2A C subunit Antibody Cell signaling 2492
NRF2(D1Z9C)
Rabbit mAb Cell Signaling 12721
Akt (pan) (11E7) Rabbit mAb Cell signaling 4685
p-Akt (ser473) (193H12) Rabbit mAb Cell signaling 4085
Mouse monoclonal O-GlcNAc (CTD110.6) Biolegend 38004
Mouse monoclonal a-Tubulin Sigma-Aldrich T9026
b-Actin (13E5) Rabbit mAb Cell signaling 4970
G6PD Bethyl A300-404A
6PGD Gene Tex GTX101703
Anti-GST (HRP) Abcam ab58626
Anti-p-CREB Cell signaling 9198
Anti-CREB Cell signaling 9197
Anti-p-VASP Cell signaling 84519
Anti-VASP Cell signaling 3132
Anti-p-p90RSK Cell signaling 8753
Anti-p90RSK Cell signaling 9355
Anti-p-p70S6K1 Cell signaling
9234
Anti-p70S6K1 Cell signaling 2708

Validation

All antibodies used herein are commercially available and have been validated by the companies that provide them.

Eukaryotic cell lines

Policy information about cell lines

Cell line source(s)

ATCC or established in the laboratory.

Authentication

All cell lines were received from ATCC and are NOT on the latest version (Vs. 8.0) of ICLAC list of misidentified cell lines. All lines used were tested for mycoplasma contamination and confirmed negative of infection via PCR through the UIC core.

Mycoplasma contamination

Cell lines used were tested for mycoplasma contamination and confirmed negative of infection via PCR through the UIC core.

Commonly misidentified lines
(See ICLAC register)

Not relevant

Animals and other organisms

Policy information about studies involving animals; ARRIVE guidelines recommended for reporting animal research

Laboratory animals

C57BL/6, Balb/cj, and NOD.Cg-Prkdscid female mice.

Wild animals

None

Field-collected samples

Not relevant

Ethics oversight

All animal work was conducted according to the ethical regulations set forth by the federal government, as reviewed and approved through the Office of Animal Care and Institutional Biosafety (OACIB) within the Office of the Vice Chancellor for Research.

Note that full information on the approval of the study protocol must also be provided in the manuscript.
